# Histidine-specific bioconjugation *via* visible-light-promoted thioacetal activation†

**DOI:** 10.1039/d2sc02353a

**Published:** 2022-06-27

**Authors:** Chuan Wan, Yuena Wang, Chenshan Lian, Qi Chang, Yuhao An, Jiean Chen, Jinming Sun, Zhanfeng Hou, Dongyan Yang, Xiaochun Guo, Feng Yin, Rui Wang, Zigang Li

**Affiliations:** State Key Laboratory of Chemical Oncogenomics, School of Chemical Biology and Biotechnology, Peking University Shenzhen Graduate School Shenzhen 518055 P. R. China lizg.sz@pku.edu.cn; Pingshan Translational Medicine Center, Shenzhen Bay Laboratory Shenzhen 518118 P. R. China yinfeng@szbl.ac.cn wangrui@szbl.ac.cn lizg@szbl.ac.cn; College of Chemistry and Chemical Engineering, Zhongkai University of Agriculture and Engineering Guangzhou 510225 P. R. China

## Abstract

Histidine (His, H) undergoes various post-translational modifications (PTMs) and plays multiple roles in protein interactions and enzyme catalyzed reactions. However, compared with other amino acids such as Lys or Cys, His modification is much less explored. Herein we describe a novel visible-light-driven thioacetal activation reaction which enables facile modification on histidine residues. An efficient addition to histidine imidazole N3 under biocompatible conditions was achieved with an electrophilic thionium intermediate. This method allows chemo-selective modification on peptides and proteins with good conversions and efficient histidine-proteome profiling with cell lysates. 78 histidine containing proteins were for the first time found with significant enrichment, most functioning in metal accumulation in brain related diseases. This facile His modification method greatly expands the chemo-selective toolbox for histidine-targeted protein conjugation and helps to reveal histidine's role in protein functions.

## Abstract 2

Functionalization of histidine residues in proteins *via* visible-light-promoted thioacetal activation is reported. ∼2000 proteins with reactive and exposed histidine residues from the MCF7 cell line are characterized using ABPP by this method.

## Graphical abstract

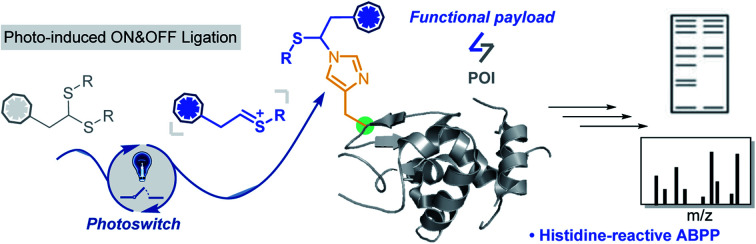

## Introduction

Post-translational modifications (PTMs) of proteins enable a marked increase in protein functional diversity^1^ for manipulation of protein structure and function.^2^ Various chemical tools have been developed to assess modified sites of proteins, exploring a large quantity of important information about protein interactions and/or enzymatic mechanisms in drug development, molecular biology and medicine.^3–6^ However, most labeling strategies are limited to the functionalization of nucleophilic residues, namely cysteine, lysine and tyrosine.^7–13^ Continuous efforts have been invested to target less-explored amino acids, including hydroxyl (threonine/serine),^14^ carboxyl (aspartic acid/glutamic acid),^15^ tryptophan,^16–21^ and methionine.^22–24^

The His residue has an electron-deficient heteroaromatic imidazole side chain and a low abundance of ∼2.2% (ref. 25) in proteins. It plays many important roles in protein functions including as a hydrogen bond donor/acceptor, and in proton shuttling, metal binding coordination (Scheme S1a†),^26–30^ metal-directed covalent modification (Scheme S1b†)^31^ and nucleophilic catalysis.^32–36^ Histidine phosphorylation has been extensively studied using neutral loss fragmentation^37^ and has garnered increasing interest in recent years;^38^ however, there is still no robust modification method specifically targeting the histidine residue.

**Fig. 1:**
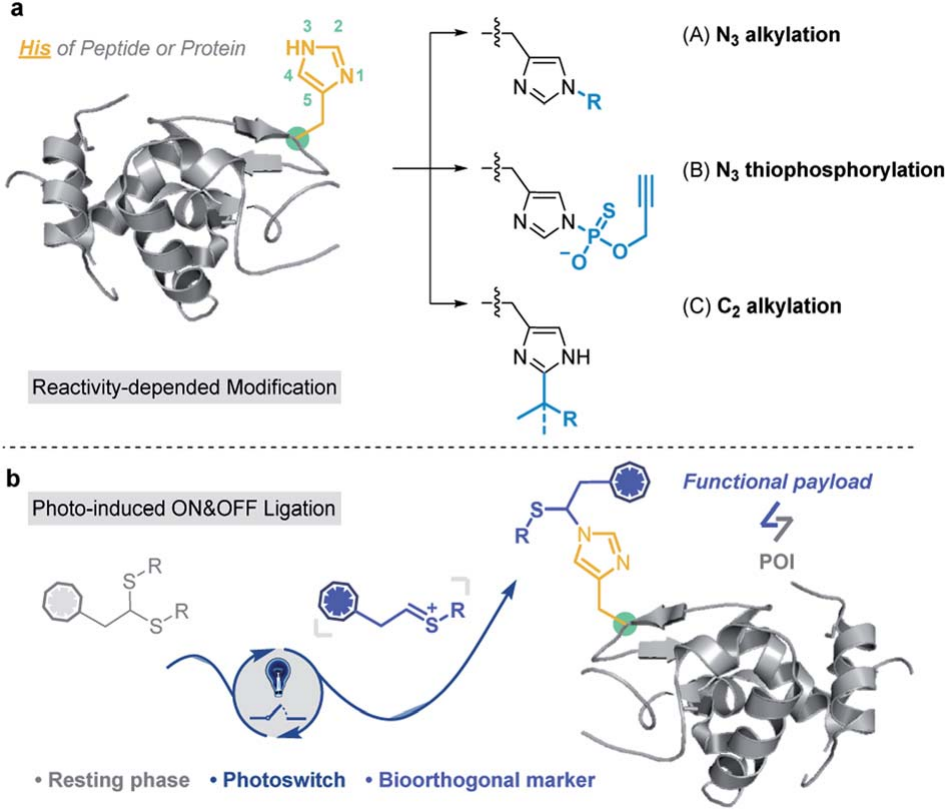
Histidine-specific bioconjugation: (a) typical methods for His-specific modification of peptides and proteins; (b) visible-light-promoted His-specific bioconjugation.

Based on the nucleophilicity of the NH ring, Hamachi *et al.* reported an affinity-based labeling strategy to modify protein His residues *via* epoxide ring opening (Fig. 1a and Scheme S2a†).^39^ A direct modification of His-tags by thiophosphorylation under weakly alkaline conditions (pH 8.5) (Fig. 1a and Scheme S2b†) was reported by Chang *et al.*^34^ In addition to the modification on the N^3^ position of His residues, Wang and Chen *et al.* developed a selective C–H alkylation on the C^2^ position of His in peptides and/or proteins *via* a visible-light-promoted approach (Fig. 1a and Scheme S2c†) with the requirement of a strong acid (trifluoroacetic acid, TFA) and organic solvent (2,2,2-trifluoroethanol, TFE).^40^ Besides, sequence-dependent strategies were demonstrated for PEGylation of protein His residues (Scheme S2d†).^41,42^ Recently, Nakamura *et al.* utilized a nucleophilic small molecule (1-methyl-4-arylurazole) to selectively label histidine under singlet oxygen (^1^O_2_) conditions generated with a ruthenium catalyst under white LED light.^36^ However, a biocompatible and selective modification of His on proteins still remains a challenge for chemo-proteomic study.

Organic sulfur(iv) molecules, such as sulfonium, are generally electrophilic and can undergo classical nucleophilic substitution reactions as a suitable leaving group in S_N_Ar reactions.^43–45^ For example, the methyltransferase-catalyzed nucleophilic methylation reaction between *S*-adenosylmethionine (SAM) and the histidine residue of proteins is an important PTM besides arginine and lysine methylations.^46,47^ On the other hand, thionium is an important intermediate state of Pummerer-type reactions, and is a highly active substrate in nucleophilic S_E_Ar reactions.^48–53^ Recently, our group developed various sulfonium-based chemistries for bioorthogonal applications with excellent biocompatibility.^54–56^ Thus, we considered the application of electrophilic thionium intermediates for protein modification. Herein, we demonstrated a specifically biocompatible method for His-specific modification on proteins *via* visible-light-promoted nucleophilic substitution. After reaction condition screening and optimization, an optimal condition for His modification was achieved in a biocompatible manner. ∼2000 reactive and exposed histidine-containing proteins were characterized from the MCF7 cell line using activity-based protein profiling (ABPP) (Fig. 1b).

## Results and discussion

### Design and condition optimization

Lewis acid catalysed Pummerer-type S_E_Ar reactions are well documented for aromatic compound modifications (Scheme S3†).^51,52,57^ However, the reaction conditions, such as organic solvents, Lewis acids and heating/cooling are generally bio-incompatible. The visible-light triggered cleavage of the C–S bond was also reported as an efficient pathway to provide electrophilic cationic intermediates.^53,58^ Thus, we checked the possibility of thioacetal 1a as a precursor of thionium for the Pummerer-type S_E_Ar reaction under visible light in the absence of Lewis acids. To develop a more bio-compatible condition, the reaction between Boc-His-OMe 2 and thioacetal 1a in MeCN/20% H_2_O (MeCN used in condition screening to avoid solubility issues) was performed under irradiation with blue LEDs (10 W, 450 nm). But this reaction gave very limited product. Then Lewis acids, transition metals and organic photocatalysts (see Fig. S1†) were examined in weakly polar MeCN/H_2_O solvent to study their effects. Rose Bengal (RB) was found to be the most efficient catalyst (10 mol% loading) (Table S1†, entries 1–13) with excellent yield (84%, Table 1, entry 1). The kinetic study was conducted at 4-fold excess of 1a with RB as catalyst, and the reaction reached equilibrium at 1 hour (Fig. S2†). Other conditions were further tested, including dark, 50 °C heating, the absence of catalyst, lower catalyst loading (Table 1, entries 2–5) or non-blue LED light irradiation (Table S1†, entries 14–17). The results indicated that the reaction is initiated by light and favours blue light. The use of iridium and MesAcrClO_4_ catalyst gave lower isolated yields (39% and 66%, Table 1, entries 6 and 7). Prolonging the reaction time (to 4 h) gave very limited improvement (85%, Table 1, entry 8). The addition of AcOH and (NH_4_)_2_CO_3_ had a negligible effect on the reaction (83% and 80%, Table 1, entries 9 and 10), suggesting that the reaction is robust and tolerates bio-relevant pHs. Notably, due to the newly introduced chiral center on the imidazole ring, the products are mixtures of a pair of epimers. Also, the reacted position was further confirmed to be N3 in the imidazole ring by 2D NMR analysis (see detailed data and discussion in the ESI†).

**Table 1:** Optimization of the photocatalyzed reaction of Boc-His-OMe 2 and thioacetal 1a

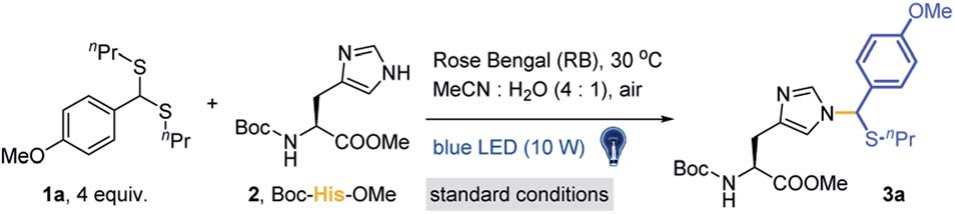		
Entry	Change from standard conditions^a^	Yield^b^ (%)
1	None	84
2	Dark	Trace^c^
3	50 °C in the dark	Trace^c^
4	Catalyst is absent	<10
5	5% catalyst loading	68
6	[Ir(ppy)_2_(dtbpy)]PF_6_ instead of RB	39
7	MesAcrClO_4_ instead of RB	66
8	4 h instead of 1 h	85
9	Addition of AcOH (2 equiv.)	83
10	Addition of (NH_4_)_2_CO_3_ (2 equiv.)	80
11	Addition of TEMPO (1 equiv.)	Trace^c^

a Standard conditions: thioacetal 1a (20 mM), Boc-His-OMe 2 (5 mM) and 10 mol% Rose Bengal (RB) in MeCN/H_2_O (4/1) under irradiation with a blue LED (10 W) at 30 °C under air for 1 hour.

b Isolated yield obtained by column chromatography.

c Determined by LC-MS. TEMPO = 2,2,6,6-tetramethylpiperidine-1-oxyl.

The chemo-selectivity of this reaction was then checked and no reaction was detected for Phe, Tyr, Trp, Ser, Lys, Arg, Glu and Gln (Fig. S3 and S4†). Due to the potential oxidative conditions, the reaction of Met was checked, and about 62% oxidative conversion (sulfoxide) was observed. Taking the low abundance of Met (∼2%)^59^ in the human proteome and the highly reductive cellular circumstances into consideration, Met oxidation is not likely to have a significant effect in protein profiling. It was discovered that few proteins changed as a result of oxidative damage in accordance with former reports.^60^ Notably, the indole side chain of Trp reacted well with thioacetal 1a to give the S_E_Ar product in TFE under irradiation with blue light (450 nm); however, no reaction was detected in aqueous media, which was also confirmed in our following profiling study (Scheme S4 and Fig. S4†). The thiol side chain of Cys rapidly reacts with 1a under acidic conditions (both Lewis acid and Brønsted acid), but only gives a trace amount of substitution product under optimized conditions (Fig. S5†). In addition, the stability of product 3a was checked under acidic conditions, including pH = 4 to 7 PBS buffer and HCOOH or TFA solvents. The results indicated partial hydrolysis of the product in weakly acidic PBS buffer after 24 hours (Fig. S6a†) and almost complete hydrolysis to 2 and 4-methoxybenzaldehyde in strong Brønsted acid-H_2_O solvent (Fig. S6b†).

**Fig. 2:**
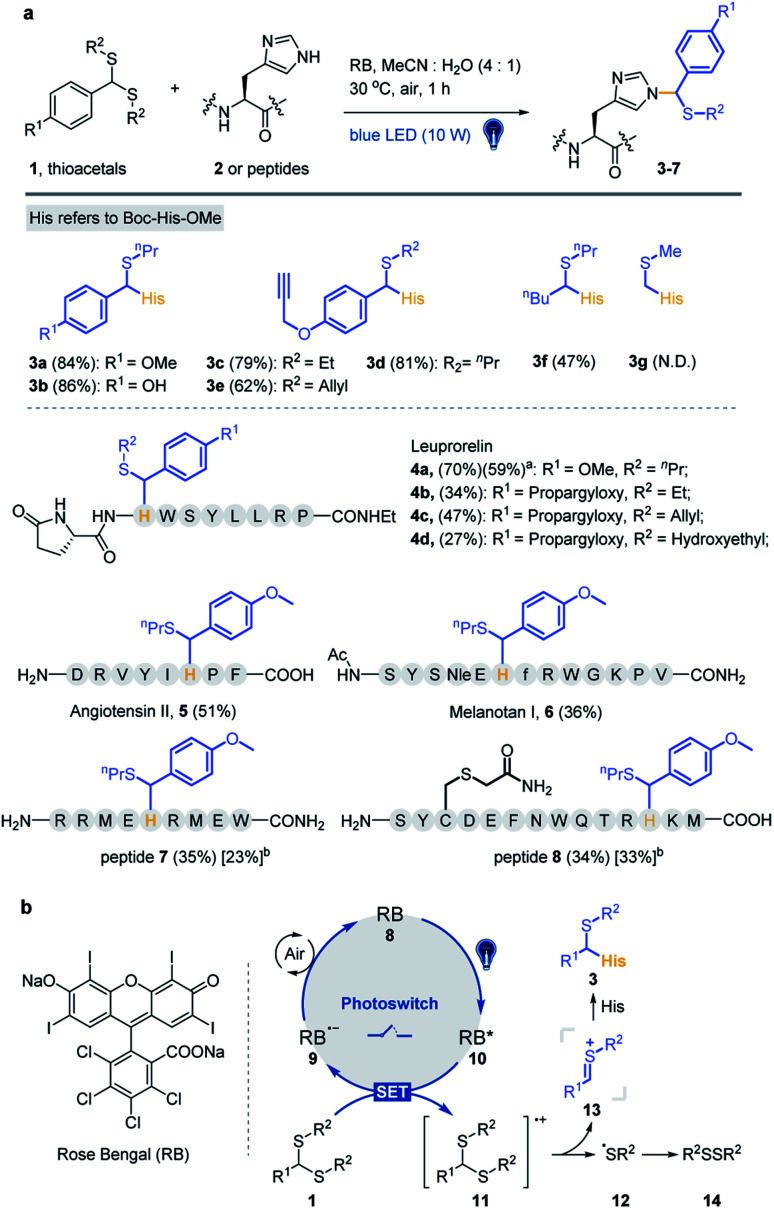
Visible-light-promoted Pummerer-type reaction between thioacetal and the side chain of His. (a) Reaction between thioacetals 1 and Boc-His-OMe/peptides. Standard conditions: thioacetal (20 mM), Boc-His-OMe 2 (5 mM) or peptide (0.5–1 mM) and 10 mol% RB in MeCN/H_2_O (4/1, pH 7.4) under irradiation with a blue LED (10 W, 450 nm) at 30 °C under air for 1 h. The % yields of peptide products were determined from reverse-phase HPLC-MS with an internal standard (dibenzyl sulfoxide). ^a^Isolated yield. ^b^The yields in square parentheses refer to thioacetal-adducted products with Met oxidation. (b) Proposed mechanism.

Various substrates and peptides were prepared and reacted under the optimized conditions to further clarify the reaction between thioacetal and the His side chain. As shown in Fig. 2a, the substrates derived from hydroxy and propargyloxy benzaldehyde were observed to have similar yields (86% for 3b and 81% for 3d), and the thioacetals of ethanethiol and prop-2-ene-1-thiol provided slightly decreased yields (79% for 3c and 62% for 3e). The reaction of pentanal-propane-1-thiol acetal only gave a moderate yield (47% for 3f), and no product was detected with formaldehyde thioacetal (3g). Three bioactive peptides, leuprorelin, angiotensin II and melanotan I, were applied for the further study of chemo-selectivity of this reaction. Four thioacetal substrates were reacted with peptide 4, and good yields were observed for 4a (70%). Likewise, the reaction of ethanethiol, prop-2-ene-1-thiol and hydroxyethyl derived thioacetals provided relatively lower yields (34% for 4b, 47% for 4c and 27% for 4d). The standard reaction was conducted between 1a and peptide 5 (51% yield) and peptide 6 (a Lys containing 13 AA peptide). Even though slightly lower yield was observed (36%) for peptide 6, the MS/MS analysis gave further evidence for the chemo-selectivity of histidine modification (see the ESI† for additional data). Also, the Met containing peptides 7 and 8 were designed for the study of influence of the oxidative conditions. For the double-Met containing peptide 7, 35% yield of thioacetal adducted products and 23% yield of products with both oxidized Met and modified His were observed under the standard conditions. Furthermore, peptide 8, which contains all of the 14 reactive AA residues, was designed for the chemo-selectivity study. With the protection of acetamide on Cys, 34% target product and 33% Met-oxidation thioacetal-adducted product were observed (see the ESI† for additional data). As a result, the reaction exhibited good efficiency and chemo-selectivity, and the desired reaction is more likely to occur than oxidative side reactions. In addition, dithiol, mercaptoethanol and mercaptoethylamine derived acetals, thioketals and α-carbamoylsulfides were examined and no desired products were detected under optimized conditions (Fig. S7†).

To clarify the reaction pathway, 2,2,6,6-tetramethylpiperidine-1-oxyl (TEMPO) was added under optimized conditions, and the reaction was largely inhibited (Table 1, entry 11), suggesting that a radical mechanism is involved.^53,61,62^ The Stern–Volmer fluorescence quenching experiments suggest an energy transfer event between the excited-state RB and the thioacetal 1a in a dose-dependent manner (Fig. S8a and S8b†). A reductive quenching mechanism is proposed in Fig. 2b. The reaction is initiated by irradiating RB 8 to its excited state with 10 W blue light and the strong single-electron oxidant 9 undergoes single-electron-transfer (SET) with thioacetal 1 to generate RB radical anion 10 and sulfur radical cation 11. Subsequent loss of the sulfur radical from 11 furnishes thionium 13 and thiyl radical 12 which dimerizes to yield disulfide 14 (detected by GC-MS in Fig. S8c†). The transient intermediate 13 then undergoes nucleophilic addition with the 3-NH of His imidazole to afford the desired product 3. Meanwhile, the photocatalyst RB is regenerated by the oxidation of 9 by air.

### Selective labeling in model protein and cell lysates

**Fig. 3:**
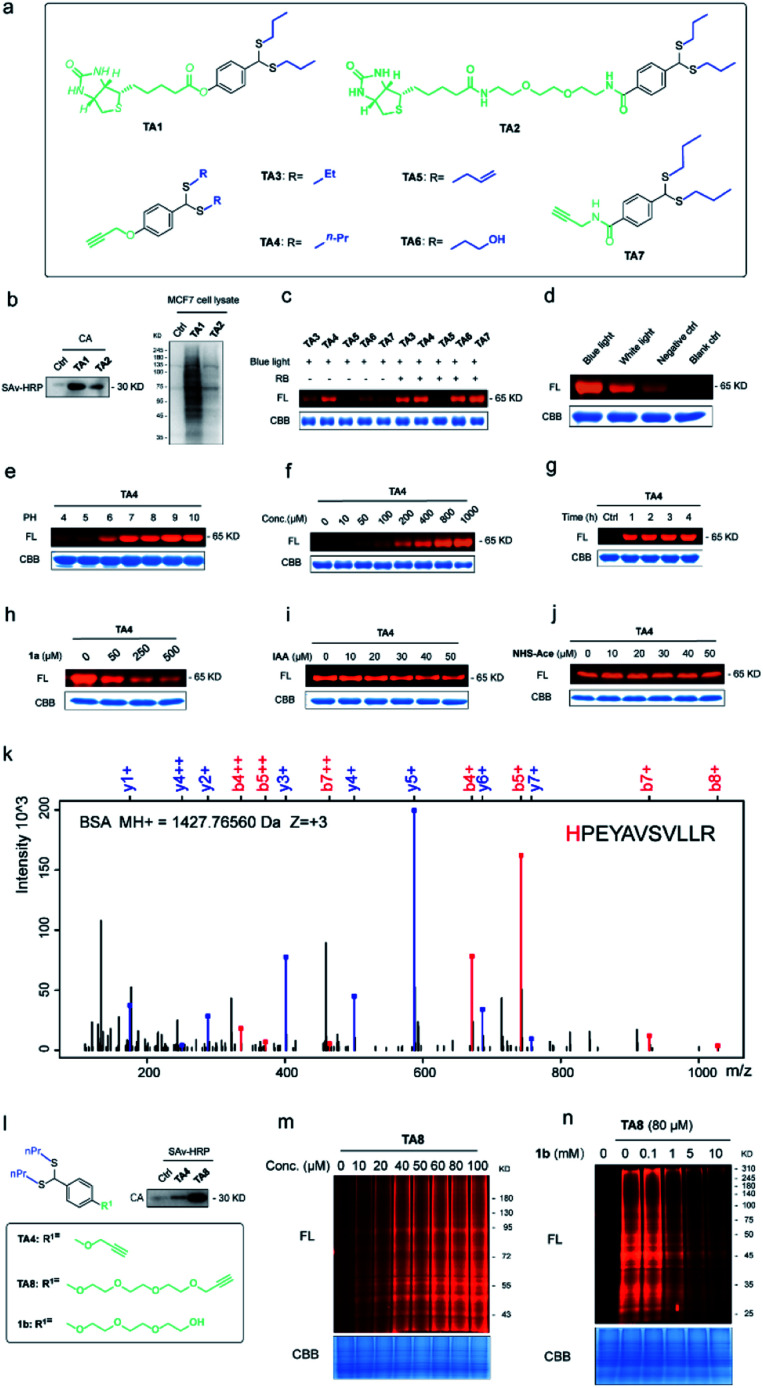
Selective histidine labeling. (a) Structures of thioacetal probes. (b) Labeling of TA1 and TA2 with CA (CA/TA1 and TA2 10/200 μM, 5% RB, pH 7.4, 37 °C for 2 h under blue light (10 W, 450 nm)) and MCF7 cell lysates (40 μg). (c) Labeling of BSA with the probes TA3–7 (BSA/TA3–7 10/200 μM, pH 7.4, 37 °C for 2 h under blue light). FL = in-gel fluorescence scanning. CBB = Coomassie gel. (d) BSA (10 μM) incubated with TA4 (200 μM) with or without light. (e) BSA (10 μM) incubated with TA4 (200 μM) in phosphate buffer with different pHs. (f) Dose-dependent labeling of BSA (10 μM) with TA4 for 2 h. (g) Time-dependent labeling of BSA (10 μM) with TA4 (200 μM). (h) Competitive labeling of BSA (10 μM) with TA4 (200 μM) in the presence or absence of competitor 1a. (i) Labeling of BSA (10 μM) with TA4 (200 μM) in the presence or absence of IAA. (j) Labeling of BSA (10 μM) with TA4 (200 μM) in the presence or absence of NHS-Ace. (k) Analysis of the amino acid specificity of TA4 with IAA-pretreated BSA. (l) TA8 did better labeling with CA (BSA/TA4 and TA8 10/200 μM, 1% RB, pH 7.4, 37 °C for 2 h under blue light). (m) Dose-dependent labeling of MCF7 cell lysates with TA8. (n) Competitive labeling of MCF7 cell lysates with TA8 in the presence or absence of competitor 1b.

To expand the chemo-selective reaction of the thioacetal group on histidine residues, biotin-modified thioacetal probes (TA1 and TA2 in Fig. 3a) were synthesized and incubated with model protein carbonic anhydrase (CA, containing histidine residues but no cysteine residue) followed by the western blot. Two clearly visible bands confirmed the histidine labeling on CA (Fig. 3b). They also confirmed histidine labeling in the complex context of MCF7 cell lysates but TA2 showed a reduced labeling efficiency (Fig. 3b). Then a series of alkyne-tagged probes (TA3–7, Fig. 3a) were prepared and incubated with model protein BSA in PBS (pH 7.4) for 2 h at 37 °C with 5% RB under blue light. Upon CuAAC “click” chemistry^61^ with rhodamine-azide (TAMRA-N_3_), the reaction mixtures were subjected to SDS-PAGE and analyzed by in-gel fluorescence scanning. The strongest fluorescence was observed from TA4 in PBS buffers with or without RB (Fig. 3c). Blue light was also found to be the most efficient among lights from different sources (Fig. 3d) and TA4 showed stronger labeling under weakly alkaline conditions (Fig. 3e). Further kinetics and stoichiometry study indicated that 20 equiv. of thioacetal probe with 2 h reaction time was sufficient for labeling (Fig. 3f–g). The fluorescence intensity of the labeled BSA band was further inhibited with increased competitor 1a (Fig. 3h), revealing that the labeling was thioacetal-dependent. Pretreatment with commercial cysteine-reactive reagent IAA (iodoacetamide) decreased the fluorescence intensity (Fig. 3i), but there was no obvious change observed from treatment with lysine-specific reagent NHS-Ace (Fig. 3j). ESI-TOF MS analysis indicated quantitative 1a labeled myoglobin (MB, containing histidine residues but no cysteine residue, Fig. S9a†). Then the labeled peptides on BSA were analyzed by LC-MS/MS (Fig. 3k and Table S3†), verifying that TA4 selectively labeled histidine in BSA when IAA was applied to block cysteine.

To increase the water-solubility of TA4, we synthesized TA8 (Fig. 3l) to label proteins followed by CuAAC “click” chemistry with biotin-dadps-azide (DADPS biotin-N_3_). It's found that TA8 did better labeling with CA protein (Fig. 3l). Therefore, we chose TA8 to evaluate the proteome reactivity profiles in breast cancer cell line MCF7. We for the first time confirmed the histidine labeling of TA8 with CA by LC-MS/MS (Fig. S9b and Table S2†). Concentration-dependent labeling profiles showed that TA8 gave visible bands at 20 μM and reached saturation at 80 μM *in vitro* (Fig. 3m). Pretreatment with 1b showed a weaker band throughout the whole lane (Fig. 3n), which was consistent with that of competitive labeling with BSA (Fig. 3h). Consistent with the above results, IAA pretreatment slightly decreased the fluorescence intensity (Fig. S9c†) and NHS-Ace pretreatment didn't affect the fluorescent signal (Fig. S9d†).

### Proteome-wide profiling in human cells

**Fig. 4:**
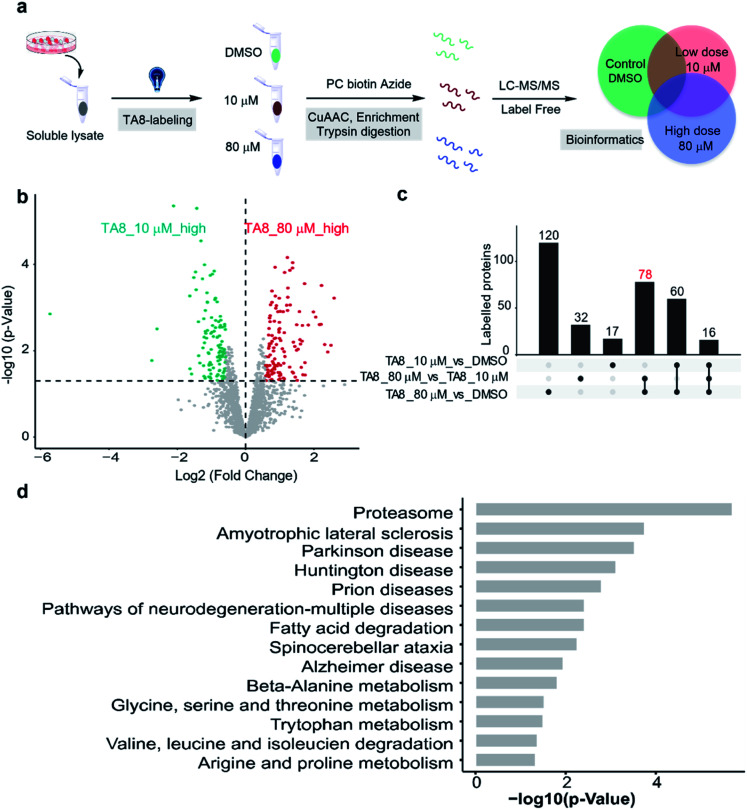
Proteome-wide quantification of reactive and exposed histidine-containing proteomes. (a) General protocol for reactive and exposed histidine-containing proteome profiling by label-free TOP-ABPP. Cellular lysates are labeled with the thioacetal-group probe (TA8) at different concentrations and DMSO control. Labeled samples are conjugated to the photo-cleavable biotin tag (green, red, and blue for DMSO, and 10 and 80 μM TA8 probe treatment groups, respectively) by CuAAC click chemistry and incubated, and TA8 labeled proteins are enriched by neutravidin-conjugated beads and digested with trypsin to yield labeled proteins for LC-MS/MS analysis. (b) Volcano plot of differentially enriched proteins under dose dependent TA8 probe groups (*n* = 3). (c) Venn diagram of highly enriched proteins in each dose-treatment group. (d) KEGG pathway analysis of highly reactive and exposed proteins in 80 μM TA8 proteome. Fold change ≥ 1.5, false discovery rate (FDR) ≤ 0.01, *q*-value ≤ 0.05. Proteins with significantly enriched proteome are shown in the ESI.†

Furthermore, we expanded the chemo-selective probes in activity-based protein profiling (ABPP) to profile reactive and exposed histidine-containing proteomes. The thioacetal group was intended to serve as a reactive electrophile; the azide-tagged version was a latent affinity handle for conjugation by “click” chemistry to biotin tags for protein enrichment^6^ (Fig. 4a). It's reasonable that the thioacetal labeled product was acid-labile; PC biotin-N_3_ is more available for acid-labile thioacetal modification, using 365 nm photo-irradiation to release the biotin tag. Subsequently, we selected PC biotin-N_3_ modified TA8 to identify reactive and exposed histidine-containing proteomes by LC-MS/MS combined with label-free quantification (LFQ) methodology. Using parallel tandem orthogonal TOP-ABPP^63^ (*n* = 3 for three groups) in MCF7 cell lysates, and dose-dependent treatment with low-dose TA8 (10 μM), high-dose TA8 (80 μM) and DMSO-control without TA8, we identified ∼1800 proteins (Fig. S10a†). Here, we used PCA (principal component analysis) to reduce the dimensionality of our datasets with a number of different variables in three experimental groups (Fig. S10b†). Comparison of TA8 (80 μM) *versus*TA8 (10 μM) (Fig. 4b) and TA8 (80 μM or 10 μM) *versus* DMSO (Fig. S10c†) indicated that 78 proteins were significantly enriched in both TA8 groups (80 μM/10 μM and 80 μM/DMSO) (Fig. 4c and Table S4–S6†). Furthermore, we sought to characterize these highly enriched proteins using KEGG pathways. Notably, in KEGG analysis, most of the enriched proteins involved the nervous-system pathways, including Alzheimer's disease and Parkinson's disease (succinate dehydrogenase complex flavoprotein subunit A, SDHA and 26S proteasome ubiquitin receptor, ADRM1) associated with metal accumulation in the brain^64^ (Fig. 4d and Table S7†). The significantly highly enriched proteasome here possibly comes from mammalian proteins of interest exhibiting histidine phosphorylation, including P-selectin, annexin I and the 20S proteasome, identified from emerging data.^65,66^ Moreover, as active histidine residues are commonly found in enzyme active sites and metal-binding sites,^67,68^ Gene Ontology (GO)^69^ terms analysis for 78 highly enriched proteins described their classes of biological process (BP), cellular component (CC) and molecular function (MF). It was indicated that reactive and exposed histidine-containing proteins were mainly involved in metabolic processes and it would be worth further studying their function, making this the first report of reactive and exposed histidine-containing proteomes to date.

## Conclusions

In summary, we have developed a biocompatible and selective approach for labeling histidine residues with thioacetal as a thionium precursor. Coupled with recent advances in photocatalysis, our work for the first time provides a selective and biocompatible chemical approach for histidine labeling in proteins *via* visible-light-driven nucleophilic substitution of the imidazole ring. We are optimistic that this method will provide a powerful tool to label histidine residues in native biological systems. Rapid labeling in living systems is currently in progress.

## Data availability

Primary data for the organic synthesis, in-gel fluorescence characterization, proteomic analysis, and bioinformatics analysis are provided in the ESI.†

## Author contributions

Research was conceived by all authors. Experiments were designed and performed by C. W., Y. W., C. L., R. W. and Z. L. Chemical synthesis was conducted by C. W., J. C., J. S. and Z. H. Biochemical assays and mass spectrometry experiments were performed by Y. W., C. L., Q. C., D. Y. and X. G. Bioinformatics analysis was performed by R. W. and Y. A. Cellular studies were conducted by Y. W., C. L. and F. Y. The manuscript was written and proofread by all authors.

## Conflicts of interest

There are no conflicts to declare.

## Supplementary Material

SC-013-D2SC02353A-s001

SC-013-D2SC02353A-s002
