# Supplementary material for: Histidine-specific bioconjugation via visible-light-promoted thioacetal activation
Source: Chem Sci. 2022 Jun 27;13(28):8289–96. doi: 10.1039/d2sc02353a (PMC9297702; doi:10.1039/d2sc02353a)
Supplement: SC-013-D2SC02353A-s002 [file SC-013-D2SC02353A-s002.pdf]

Electronic Supplementary Material (ESI) for Chemical Science.
This journal is © The Royal Society of Chemistry 2022

Supplementary Information

Histidine-Specific bioconjugation via visible-light-promoted thioacetal activation

Chuan Wan,[†a] Yuena Wang,[†a] Chenshan Lian,[†b] Qi Chang,[b] Yuhao An,[b] Jiean Chen,[b] Jinming Sun,[a] Zhanfeng Hou,[b] Dongyan Yang,[c] Xiaochun Guo,[a] Feng Yin,[*b] Rui Wang,[*b] and Zigang Li[*a, b]

[a.] *State Key Laboratory of Chemical Oncogenomics, School of Chemical Biology and Biotechnology, Peking University Shenzhen Graduate School, Shenzhen, 518055, P. R. China.*
[b.] *Pingshan translational medicine center, Shenzhen Bay Laboratory, Shenzhen, 518118, P. R. China.*
[c.] *College of Chemistry and Chemical Engineering, Zhongkai University of Agriculture and Engineering, Guangzhou, 510225, P. R. China.*
† These authors contributed equally to this work
* Address correspondence to yinfeng@szbl.ac.cn, wangrui@szbl.ac.cn, lizg.sz@pku.edu.cn, lizg@szbl.ac.cn.

Table of Contents

1. Supplementary figures and discussions 3
2. Supplementary material and methods for biochemistry 17
    2.1 General procedure 17
    2.2 Chemicals and Reagents 17
    2.3 Cell Culture 17
    2.4 Gel fluorescence analysis of probe-labelled proteins 17
    2.5 Histidine-specific profiling using mass spectrometry 18
    2.6 LC-MS/MS Analysis 18
    2.7 Data Processing 18
3. Chemical procedures and compound characterizations 19
    3.1 General procedure A and characterization for the reaction between Boc-His-OMe and thioacetal 1 19
    3.2 General procedure B for the synthesis of thioacetals and thioketals 23
    3.3 Synthesis of TA8 and TA9 27
    3.4 Synthesis of TA1 and TA10 28
    3.5 Synthesis of TA2 and TA11 29
    3.6 Synthesis of tert-butyl ((ethylthio)(4-methoxyphenyl)methyl)carbamate S8 32
    3.7 Synthesis of intermediates 32
4. Supplementary material and methods for the reactions of peptide 35
    4.1 General information 35
    4.2 General procedure C for the reaction between peptide and thioacetal 35
5. References 58
6. NMR spectra 59

1. Supplementary figures and discussions

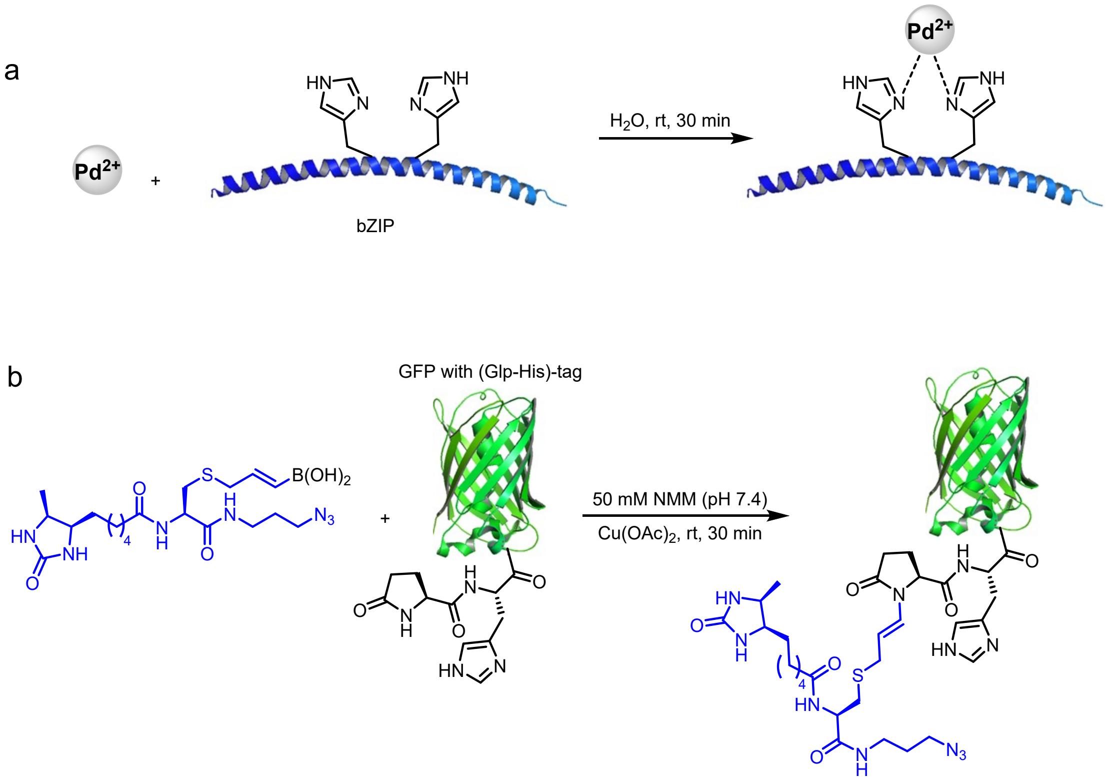

Scheme S1. Examples of metal-approaches for His modification on protein. (a) Intracellular reactions promoted by bis(histidine) miniproteins stapled using Palladium(II) complexes[1]. (b) Histidine-directed arylation/alkenylation of backbone N−H bonds mediated by Copper(II)[2].

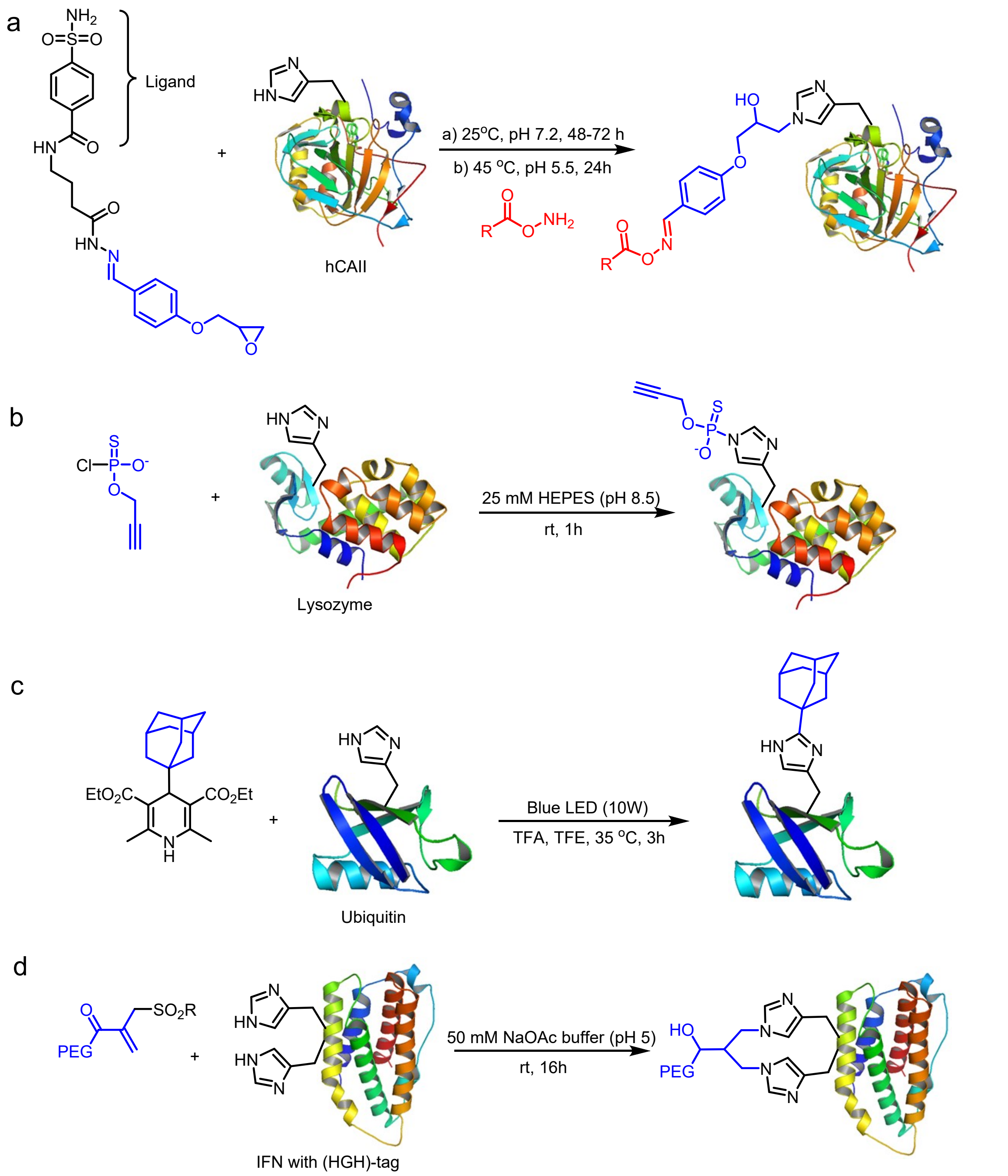

Scheme S2. Examples for His modification on protein. (a) Histidine-selective labelling at the proximity of the active site of enzyme surface[3]. (b) Histidine modification using thiophosphorodichloridate reagents that mimic post-translational histidine phosphorylation[4]. (c) peptide modification via radical-mediated chemoselective C−H alkylation of histidine using C4-alkyl-1,4-dihydropyridine (DHP) reagents under visible-light-promoted conditions[5]. (d) Site-selective protein conjugation at histidine using a bis-alkylation reagent, PEG(10kDa)-mono-sulfone[6].

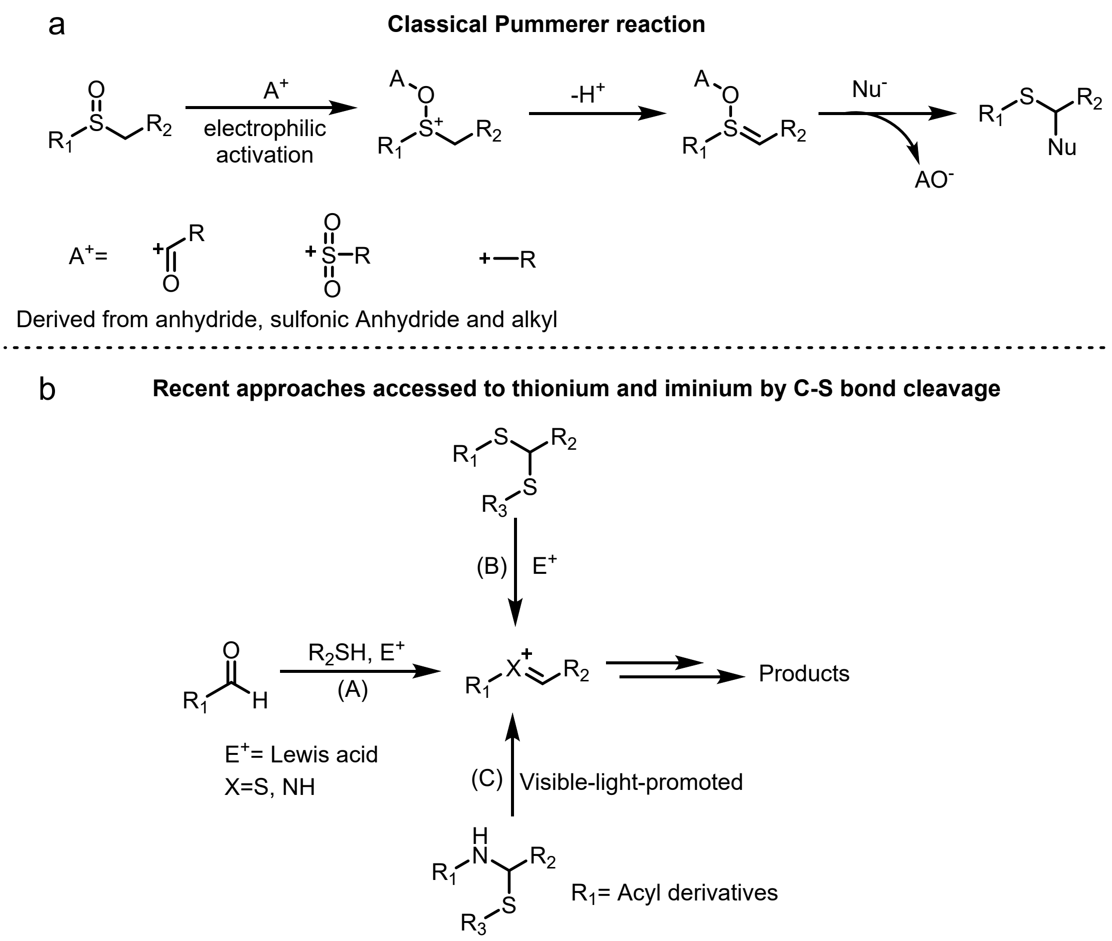

Scheme S3. Reactions accessed to electrophilic oniums. (a) Classical Pummerer reaction. (b) Recent approaches accessed to thionium and iminium by C-S bond cleavage: (A) Connective Pummerer-type reaction by the condensation of an aldehyde and a thiol; (B) ionization of thioketals and thioacetals; (C) Visible-light-promoted formation of iminiums.

Pummerer reaction is a well-studied organic transformation[7] and generally requires stoichiometric amounts of strong electrophilic activators including acid anhydride, trimethyloxonium, dimethylthiosulfonium fluoroborate (DMTSF), and TMSCl (**Scheme S3a**). Recently, thioacetals and intermediates of aldehyde-thiol condensation were well demonstrated as thionium precursor to undergo connective Pummerer-type reaction by previous literatures, where polar organic solvents, such as TFE, and catalytical Lewis acids, such as BF_3 OEt_2, $Cu(OTf)_2$ and phosphate, were required (**Scheme S3b**).[8] Therefore, we initialed our investigation by the reaction between thioacetal and aromatic amino acids to screen a biocompatible method for Pummerer-type modification of proteins. All of the aromatic amino acids (His, Trp, Tyr and Phenylalanine, Phe) were reacted with thioacetal **1a** under a polar condition (TFE solvent) with irradiation of Blue LED (10 W). The reaction of derivatives of Trp and His gave products with 89% and 71% yield (**Scheme S4a** and **Scheme S4b**), and evidences of NMR spectra validated their alkylation positions are C_2 for Trp and N_3 for His, respectively. In contrast, derivatives of Phe didn't observe any product under this condition, and trace of product of Tyr and **1a** was detected in a

more polar solvent 1,1,1,3,3,3-hexafluoroisopropanol (HFIP) (**Scheme S4**). These results proved the feasibility of the modification of aromatic amino acid by Pummerer-type reaction and encouraged us to develop more biocompatible conditions. In order to ensure the dissolution of thioacetal at a 20 mM concentration, MeCN/H_2O mixed solvent was investigated. In MeCN/20% H_2O solvent, the reaction between Boc-His-OMe and **1a** exhibited a very inefficient manner. Hence, we then shifted our focus to photoredox-catalysts.

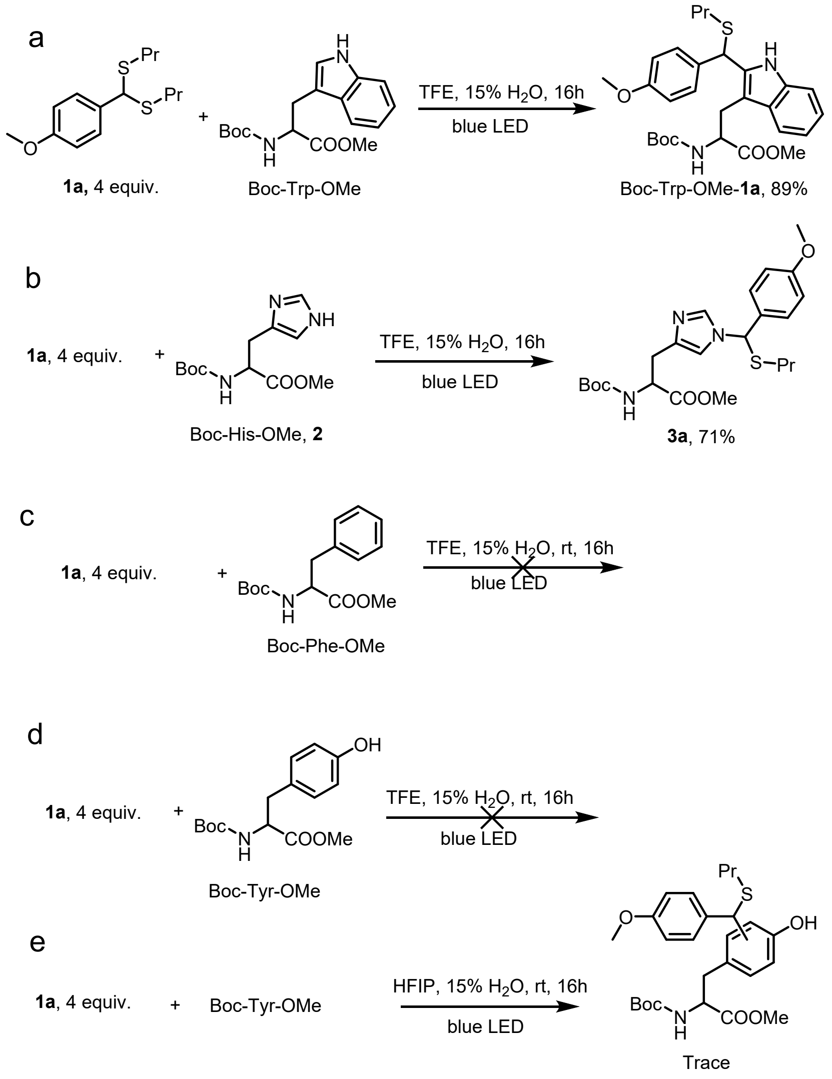

Scheme S4. Visible-light-promoted Pummerer-type reaction between thioacetal and derivatives of aromatic amino acids: (a) S_EAr reaction of Boc-Trp-OMe with TFE solvent; (b) Nucleophilic reaction of Boc-His-OMe **2** with TFE solvent; SEAr reaction of (c) Boc-Phe-OMe and (d) Boc-Tyr-OMe with TFE solvent; (e) SEAr reaction of Boc-Tyr-OMe with HFIP solvent.

Table S1. Optimization of conditions[a]

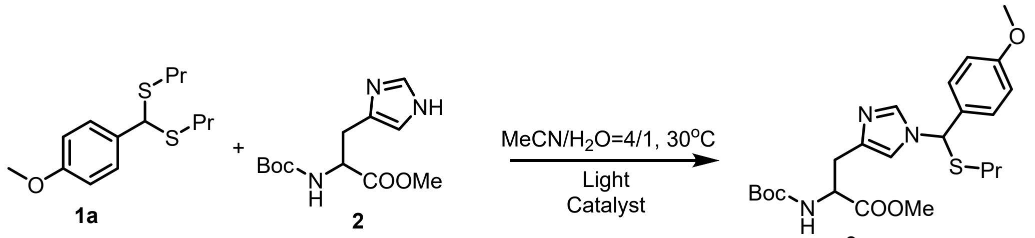

Entry	Catalyst	Light	Conversion (%)[b]
1	-	blue LED	<10
2	-	blue LED (12 h)	36
3	$Cu(OTf)_2$	blue LED	18
4	$Cu(MeCN)_4BF_4$	blue LED	33
5	$[Ir(ppy)_2(dtbbpy)](PF_6)$	blue LED	62 (39)[c]
6	$Ru(bpz)_3(PF_6)_2$	blue LED	10
7	Phenanthrene	blue LED	47
8	3,6-Di(pyridin-2-yl)-1,2,4,5-tetrazine	blue LED	44
9	$MesAcrClO_4$	blue LED	79 (66)[c]
10	1,2,4,5-Tetracyanobenzene	blue LED	52
11	Rhodamine B	blue LED	32
12	Riboflavin tetrabutyrate	blue LED	85
13	Rose Bengal	blue LED	92 (84)[c]
14	Rose Bengal	dark	trace
15	Rose Bengal	white LED	67
16	Rose Bengal	green LED	62
17	Rose Bengal	red LED	48

[a] Conditions: 20 mM thioacetal **1a** (4 equiv.), 5 mM Boc-His-OMe (1 equiv.) and 10 mol% catalyst in $MeCN/H_2O$(4/1) under irradiation of LED light (10 W) or dark at 30 °C under air for 1 hour. [b] Conversions were determined by HPLC. [c] Isolated yields in the parentheses.

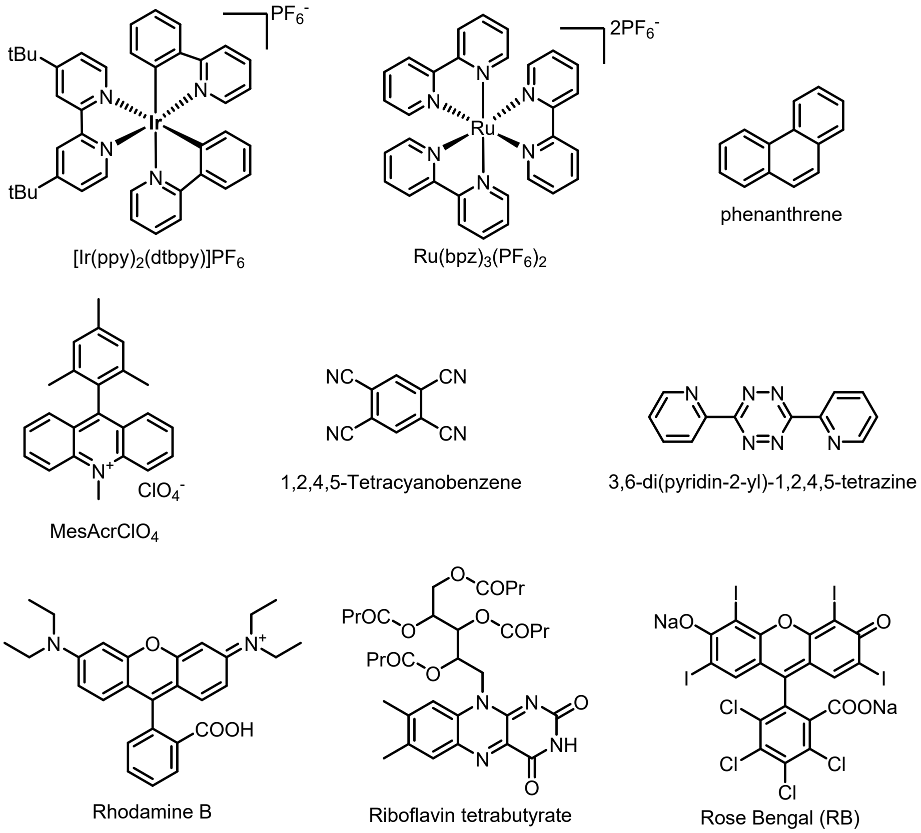

Figure S1. Chemical structures of photocatalysts used in this work.

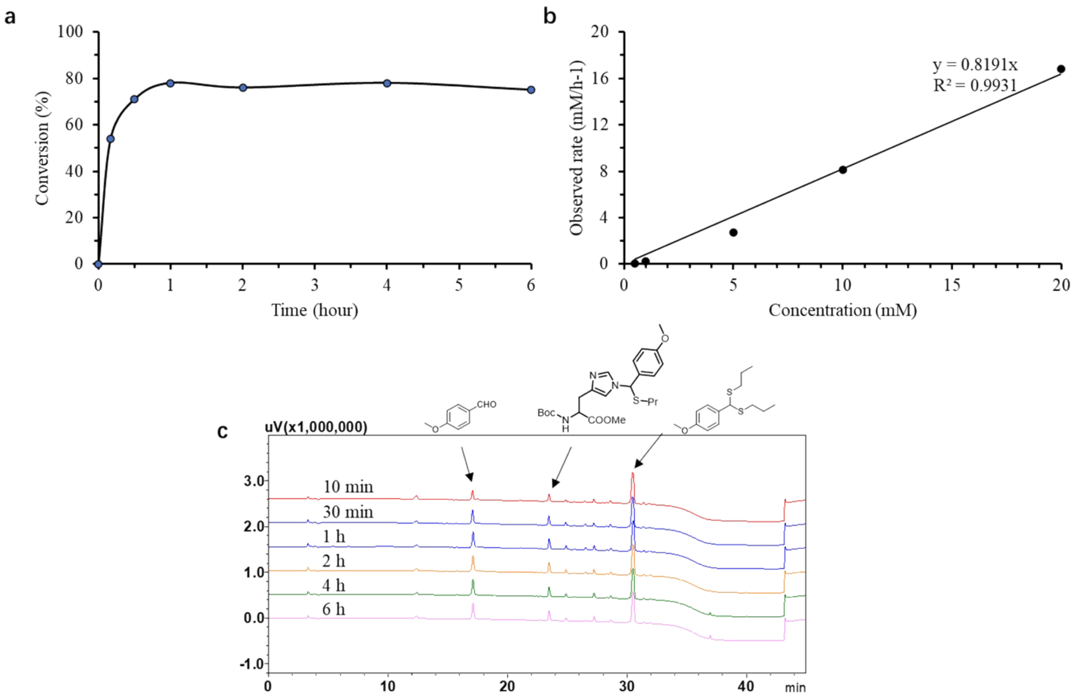

Figure S2. Characterization of the reaction kinetics between thioacetal **1** and Boc-His-OMe: (a) Estimation of the yield versus time plot by LC-MS. (b) Determination of the second-order rate constant for the reaction. The observed rates calculated from different concentrations of **1** and Boc-His-OMe were plotted against the concentration of **1** to obtain the rate constant k from the slope of the plot. (c) The LC traces of the reaction.

Conditions: thioacetal **1a** (4 equiv., 20 mM, 10m M, 5 mM, 1 mM or 0.5 mM), Boc-His-OMe (1 equiv., 5 mM, 2.5 mM, 1.25 mM, 0.25 mM or 0.125 mM) and 10 mol% Rose Bengal (RB) in MeCN/H_2O(4/1) under irradiation of blue LED (10 W) at 30 ^{o}C under air for 10 min, 30 min, 1 h, 2 h, 4 h and 6 h.

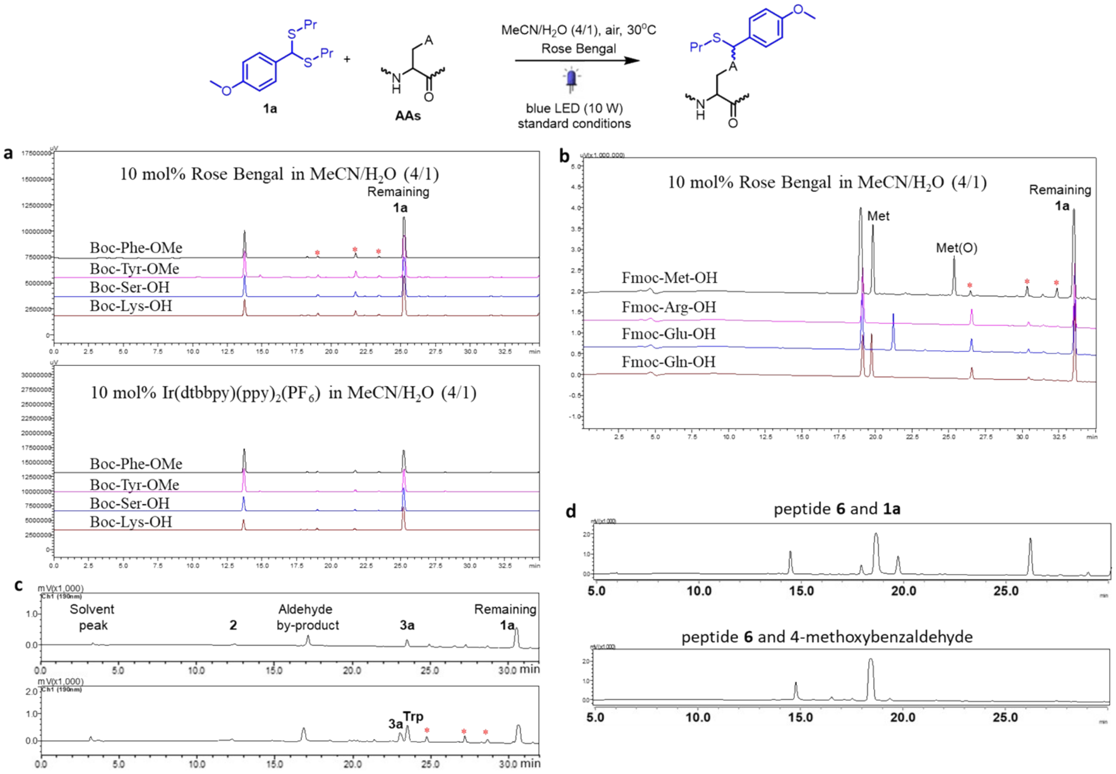

Figure S3. (a) LC-MS analysis of the reaction between various amino acids and thioacetal **1a** under optimized conditions. The red asterisk indicates trace by-products from RB and thioacetal. (b) LC-MS analysis of the reaction between additional Fmoc-protected amino acids and thioacetal **1a** under optimized conditions. (c) The chemo-selectivity between aromatic and nucleophilic AAs by LC-MS analysis *via* a one-pot reaction. Top trace: the standard reaction of **1a** and **2**; bottom trace: a mixed reaction between **2**, Boc-Trp-OMe, Boc-Phe-OMe, Boc-Tyr-OMe, Boc-Ser-OH, Boc-Lys-OH and **1a** under optimized conditions. Aldehyde by-product refers to 4-methoxybenzaldehyde produced by hydrolysis of substrate **1a**. (d) The comparison between the standard reaction of peptide **6** with **1a** and 4-methoxybenzaldehyde.

Conditions: 20 mM thioacetal **1a** (4 equiv.), 5 mM amino acid (1 equiv.) and 10 mol% catalyst in MeCN/H_2O(4/1) under irradiation of blue LED (10 W) at 30 ^{o}C under air for 1h.

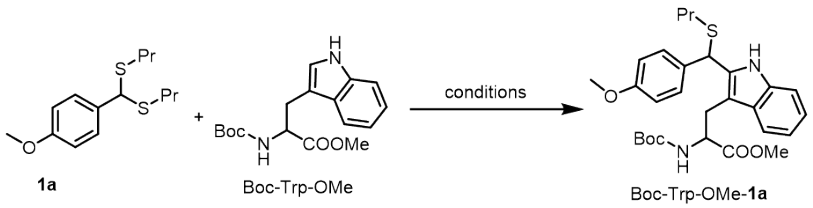

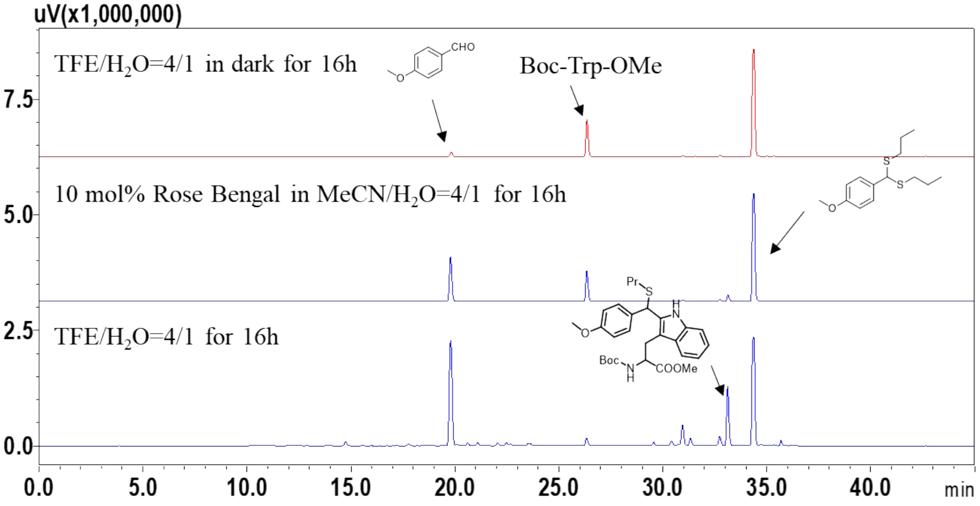

Figure S4. LC-MS analysis of the reaction between Boc-Trp-OMe and thioacetal **1a**.
Conditions: 20 mM thioacetal **1a** (4 equiv.), 5 mM Boc-Trp-OMe (1 equiv.) and 10 mol% RB in specified solvent under irradiation of blue LED (10 W) at 30 ^{o}C under air for 16 h.

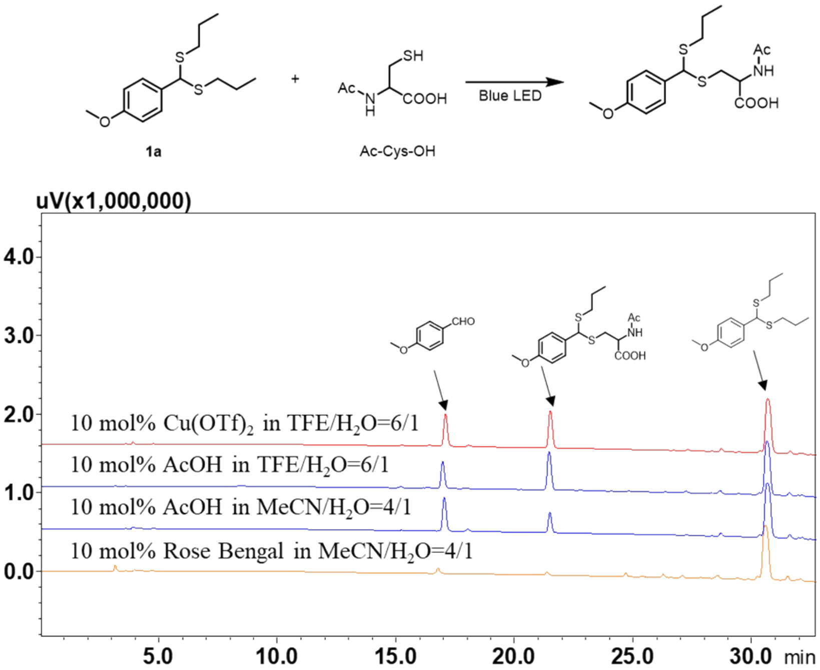

Figure S5. LC-MS analysis of the reaction between Ac-Cys-OH and thioacetal **1a**.
Conditions: 20 mM thioacetal **1a** (4 equiv.), 5 mM Ac-Cys-OH (1 equiv.) and 10 mol% catalyst or 5 mM Ac-Cys-OH (1 equiv.) in MeCN/H_2O(4/1) under irradiation of blue LED (10 W) at 30 ^oC under air for 1h.

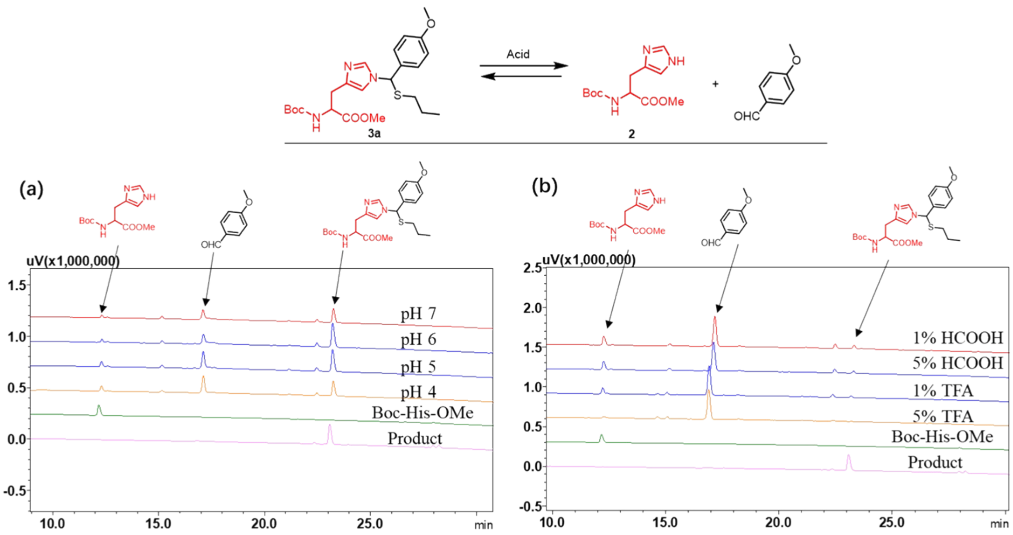

Figure S6. Hydrolysis of Boc-His-OMe product.
Conditions: 5 mM **1a** (4 equiv.) in specified solvent at 37 ˚C shaker for 24 h. (a) In PBS buffer. (b) In aqueous solution with Brønsted acid.

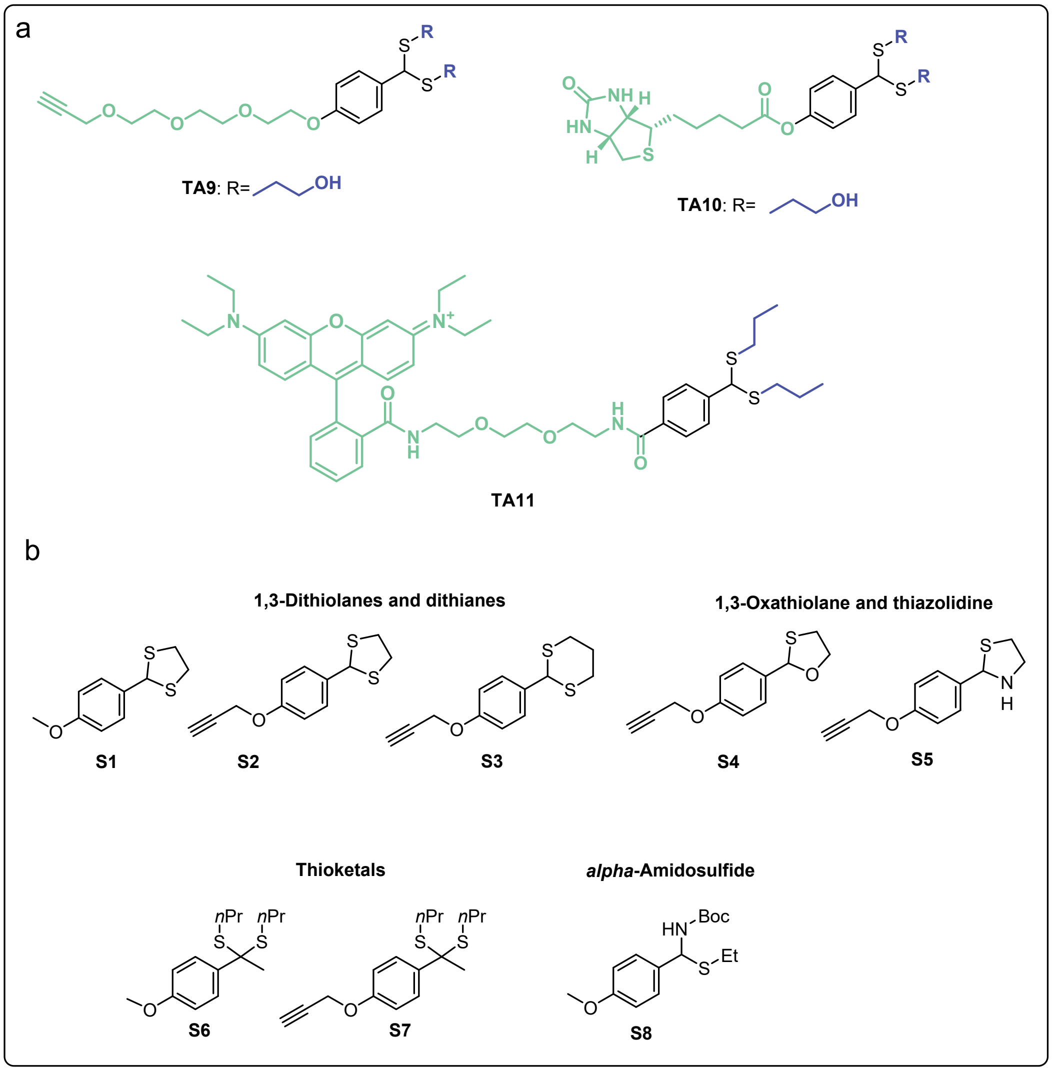

Figure S7. (a) Other Thioacetal probes used in this work; (b) Chemical structures of unreactive thioacetals and thioketals.

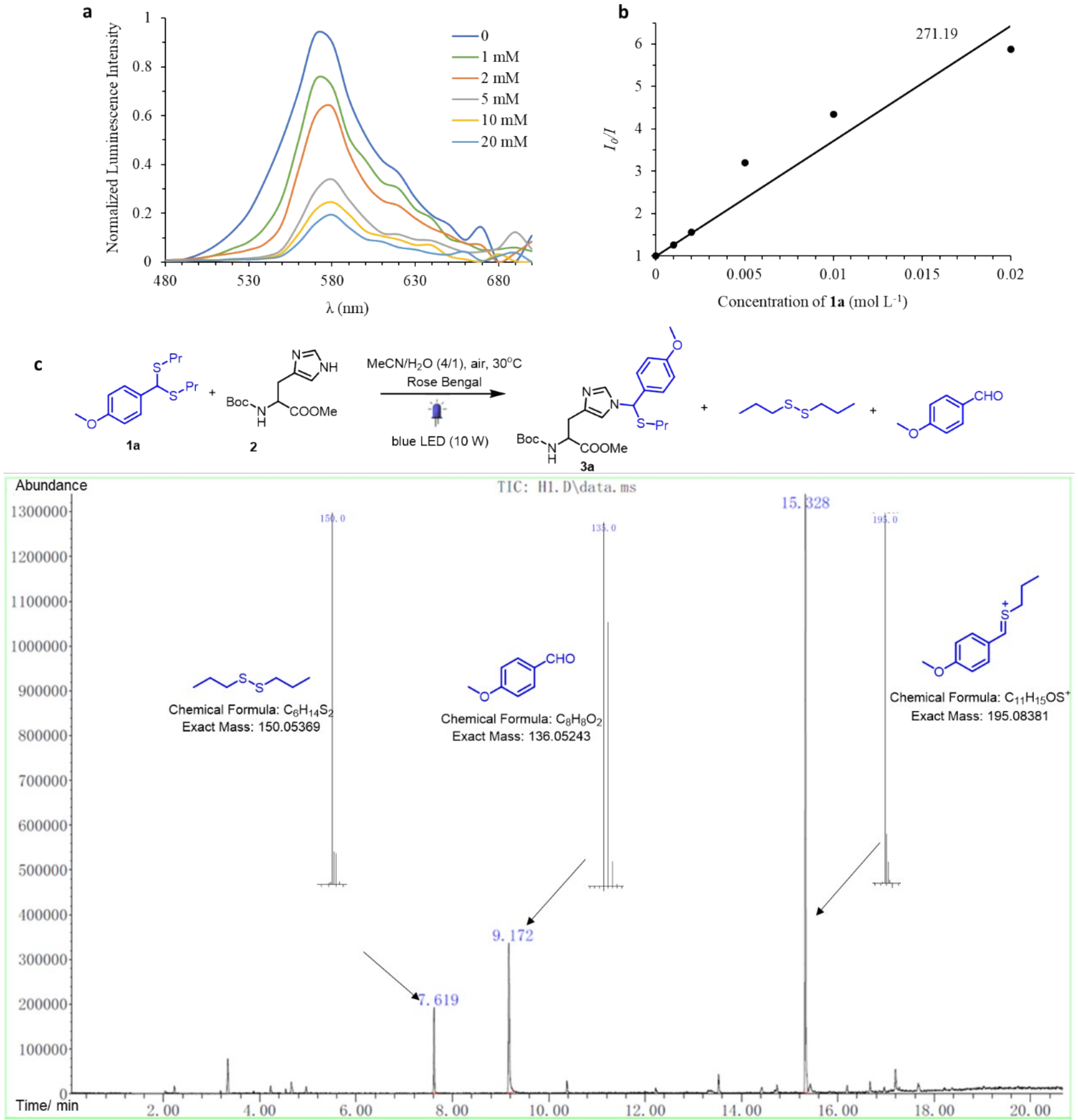

Figure S8. (**a**) Stern–Volmer quenching studies between RB (10 µM) and thioacetal **1a** (0, 1, 2, 5, 10, 20 mM, respectively). (**b**) Estimation of the I_0/I versus concentration of **1a** plot by luminescence spectra. I_0: the luminescence emission intensity of photocatalyst RB. I: the luminescence emission intensity of photocatalyst RB with specific concentration of **1a**. (**c**) GC-MS analysis for the study of mechanism. Conditions: 10 mM thioacetal **1a** (4 equiv.), 2.5 mM Boc-His-OMe (1 equiv.) and 10 mol% Rose Bengal (RB) in MeCN/H_2O(4/1) under irradiation of blue LED (10 W) at 30 ˚C under air for 1 h.

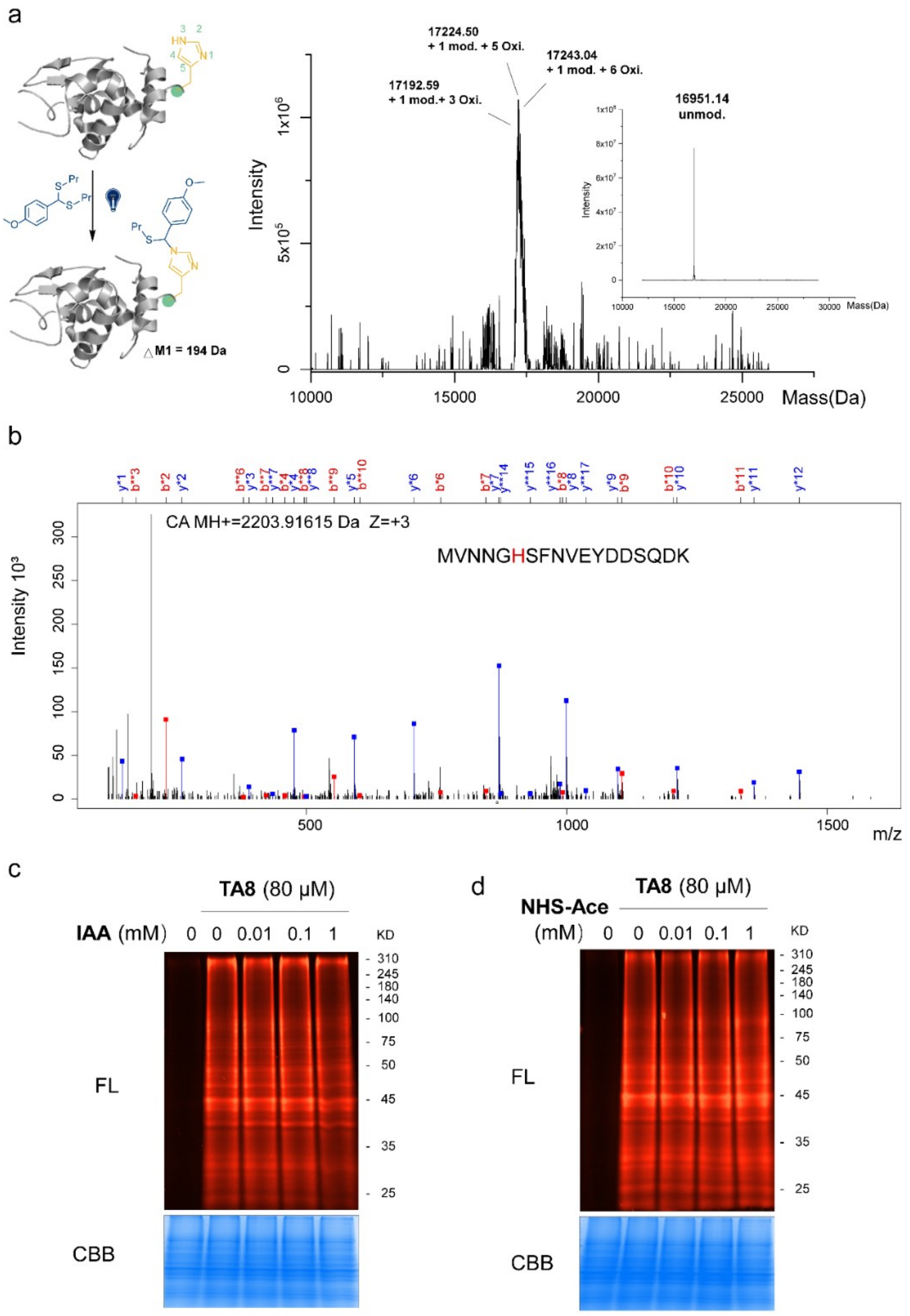

Figure S9. Selective Histidine labeling. (a) ESI-TOF Spectrum of unmodified MB and **1a** modified MB (MB/**1a** 100 μM /2 mM, 5% Rose, pH 7.4, 37 ˚C for 0.5 h under blue light). (b) Labeling of MCF7 cell lysates with **TA8** in the presence or absence of **IAA**. (c) Labeling of MCF7 cell lysates with **TA8** in the presence or absence of **NHS-Ace**.

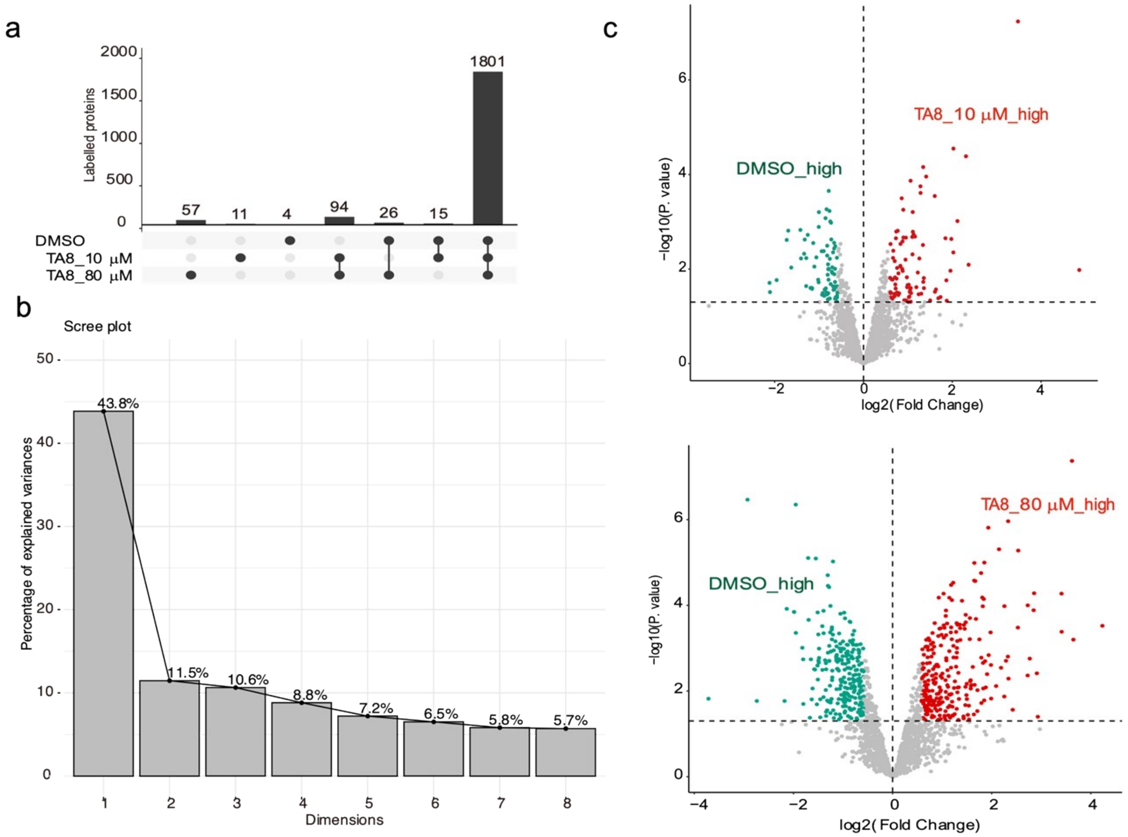

Figure S10. Histidine containing proteome quantification from Label-free TOP-ABPP (a) venny (b) PCA and (c) volcano analysis of **TA8** labelled proteome in three experimental groups.

2. Supplementary material and methods for biochemistry

2.1 General procedure

All chemical reagents are commercially available without purification. The reactions were monitored by TLC (silica gel-G). Nuclear Magnetic Resonance (NMR) spectra were recorded on Bruker 300 MHz, 400 MHz or 500 MHz spectrometer using trimethylsilane (TMS) as internal standard under ambient temperature (20 °C). High-Resolution Mass Spectrometry (HRMS) were measured on Orbitrap Exploris 480.

2.2 Chemicals and Reagents

All chemicals were purchased from Sigma-Aldrich, unless otherwise stated. Leuproprelin (peptide **4**) was purchased from Sangon Biotech; Angiotensin II (peptide **5**) and Melanotan I (peptide **6**) were purchased from Wuxi Asiapeptide Biotechnology Co. Ltd.. The procedures for the preparation of peptide **7** and **8** were illustrated in Section 4.1. Bovine Serum Albumin (BSA) was purchased from Sangon Biotech. Myoglobin (MB) and Carbonic Anhydrase (CA) were purchased from Sigma-Aldrich. TAMRA-azide (named as "TAMRA-N_3" in this study), Biotin-dadps-azide (named as "DADPS biotin-N_3" in this study) and PC biotin-PEG_3-azide (named as "PC biotin-N_3" in this study) was purchased from BIOCONE (Chengdu, CHINA). Sequencing-grade trypsin and NeutrAvidin™ agarose were purchased from Thermo Scientific. All solutions were made with ultrapure Milli-Q water (Millipore, Bedford, MA). BSA, MB and CA were dissolved in PBS buffer, pH 7.4. The peptide and probes were dissolved in DMSO to a stock concentration of 100 mM.

2.3 Cell Culture

MCF-7 cells were maintained at 37 ˚C in a humidified atmosphere containing 5% carbon dioxide using Dulbecco's modified Eagle's medium (DMEM) supplemented with 2 mM insulin and 10% fetal bovine serum (FBS). We separated cells once they grew to 80-90% confluence in a 1:2 ratio. After 12~14 hours, subculture would achieve to approximately 80% confluence on 100 mm polystyrene tissue culture plates. The cells should be in log phase growth and healthy. On ice, we discard old medium and wash cells twice with ice-cold PBS. Pre-cooled microcentrifuge tube resuspended pellet in 1 ml cold DPBS buffer. Cells were sonicated until pellet is disturbed then centrifuged at x12,000 rpm 30 min. The final supernatant protein was transferred to a new tube and quantified at 562 nm using BCA (Thermo Scientific).

2.4 Gel fluorescence analysis of probe-labelled proteins

For probe labelling assay, BSA or proteins extracted from MCF7 cells were incubated with probes at indicated concentration at 37 ˚C for 2 h with blue light. A freshly pre-mixed click chemistry reaction cocktail was then added (50 µM TAMRA-N_3, 100 µM TBTA, 1 mM TCEP, and 1 mM CuSO4) to the mixture above for another 1 h at RT. After reaction, the labelled proteins were precipitated by pre-chilled acetone (-20 ˚C) to remove excessive reagents. Precipitated proteins were subsequently collected by centrifugation (13000 rpm x 10 min at 4 ˚C) and dissolved in PBS containing 1.2% SDS and then boiled using 4× SDS-PAGE loading buffer at 95 ˚C for 10 min. The samples were analyzed by 12% SDS-PAGE gels (polyacrylamide gel electrophoresis) and imaged by Bio-Rad ChemiDoc MP. Then the gels were then stained with Coomassie staining (CBB) and

scanned. For competition assay, proteins were treated with IAA or sulfo-NHS acetate at indicated concentration as above processes.

2.5 Histidine-specific profiling using mass spectrometry

We had 3 groups of proteins named as "DMSO", "10 μM **TA8**" and "80 μM **TA-8**". Then biotin was ligated to the probe-labelled proteins in as groups with click chemistry reaction. After reaction, the labelled proteins were precipitated by pre-chilled acetone (-20 ˚C) to remove excessive reagents. Precipitated proteins were subsequently collected by centrifugation (13000 rpm x 10 min at 4 ˚C) and dissolved in PBS containing 1.2% SDS then diluted to 0.2% SDS. Upon incubated with NeutrAvidin™ agarose beads for 3 h at 29 ˚C to capture the biotin-labelled proteins, the beads were washed with cold PBS (twice) and cold water (twice). The captured proteins on beads were reduced by DTT, treated by IAA, and digested at 37 ˚C overnight by trypsin into peptides. The supernatant was collected by centrifugation (1,000 g, 1 min) and combined with 2 X 50 μl water after washing beads, stored at -80 ˚C then acidified to a final concentration of 2% (v/v) formic acid and desalted for LC-MS/MS analysis.

2.6 LC-MS/MS Analysis

After dry in the speed VAC, obtained samples were loaded onto a Thermo analytical column (75 μm i.d. × 25 cm) C18 column with an Easy-nLC 1200 chromatography pump coupled with Orbitrap Exploris^{TM}480. For each analysis, we reconstituted peptides in 15 μl 0.1% FA and loaded 4 μl onto the column for running. Peptides in each running were separated on a 120 min (5-40% ACN) gradient. Parameters are as follows in Full MS/ data dependent -MS2 TopN mode: mass analyzer over m/z range of 350−1500 with a mass resolution of 60000 (at m/z=200) in a data-dependent mode, 1.6 m/z isolation window. 20 most intense ions are selected for MS/MS analysis at a resolution of 15000 using collision mode of HCD. Because of different fragmentation, it is divided into the following types of modifications: TA2(△M=218.07654), TA2-2(△M=179.05306), TA2-3(△M=144.05751), TA6-5(△M=105.03404), TA2-PEG2-1(△M=350.15518), TA2-PEG2-2(△M=179.05306), TA2-PEG2-3(△M=276.13616).

2.7 Data Processing

Spectral data was searched against the Uniprot/Swiss-prot protein database using PD 2.4 and filtered to 1% FDR (false discovery rate) at the protein level. Default parameters used following exceptions: a minimum of 1 unique peptide was required for quantitation; peptide matching between runs was included and peptides containing oxidation (O), N-terminal acetylation (protein N-term), and carbamidomethyl (C) as variable modifications; only tryptic peptides with two missed cleavage sites were allowed; fragment mass tolerance was set to 0.02 Da for MS/MS fragment ions; mini and max peptide lengths were 6 and 144. GO enrichment was performed for cellular components of labelled proteins, and Uniprot accession numbers of identified reactive-histidine-containing proteins were subjected to LIMMA R package analysis.

3. Chemical procedures and compound characterizations

3.1 General procedure A and characterization for the reaction between Boc-His-OMe and thioacetal 1

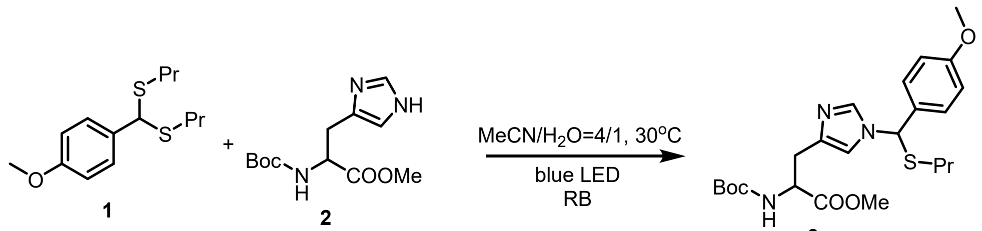

To a colorless transparent glass vial charged with an appropriate magnetic stir bar was added thioacetal **1** (108 mg, 0.4 mmol, 4 equiv.), Boc-His-OMe **2** (26.9 mg, 0.1 mmol) and Rose Bengal (10.2 mg, 0.01 mmol 10 mol%), and 20 mL of MeCN/H_2O=4/1 was added as solvent. The vial was then sealed and placed on a magnetic stirrer about 2 cm away from a 10 W blue LED lamp for 1h. After reaction completion, the organic solvents were removed on a rotary evaporator, 10 mL of H_2O was added and the crud mixture was extracted with CH_2Cl_2 (3×10 mL). The organic phase was washed with saturated NaCl solution (2×5 mL) and dried over anhydrous Na_2SO_4. The crud product was purified by flash column chromatography using eluent solution Hexane/EtOAc (4:1). **3a** (yellow oil, yield 84%): ^{1}H NMR (300 MHz, Chloroform-*d*) δ 7.73 – 7.61 (m, 1H), 7.21 – 7.09 (m, 2H), 6.96 (s, 1H), 6.86 – 6.80 (m, 2H), 6.13 (d, *J* = 1.6 Hz, 1H), 5.87 (t, *J* = 9.5 Hz, 1H), 4.53 (dt, *J* = 8.4, 4.8 Hz, 1H), 3.76 (s, 3H), 3.62 (s, 3H), 3.04 (td, *J* = 15.7, 15.3, 9.1 Hz, 2H), 2.50 – 2.43 (m, 1H), 2.34 (ddd, *J* = 12.6, 7.4, 2.1 Hz, 1H), 1.58 (ddt, *J* = 14.2, 7.3, 3.7 Hz, 2H), 1.40 (s, 9H), 0.95 (t, *J* = 7.4 Hz, 3H). ^{13}C NMR (75 MHz, Chloroform-*d*) δ 172.49, 159.78, 155.53, 136.53, 138.10, 129.97, 127.65, 115.86, 114.14, 79.60, 64.01, 55.30, 53.63, 52.06, 33.92, 30.49, 28.30, 22.17, 13.41. HRMS: (ESI): $[M+H]^+$ Calcd. m/z 464.22137, found m/z 464.22250, Error: 2.45ppm.

Characterization of the product 3a

In order to determine the reacted nitrogen atoms, we further performed three 2D NMR experiments (^{1}H-^{1}H COSY, ^{1}H-^{13}C HSQC and ^{1}H-^{13}C HMBC) for the product **3a**. The corresponding full spectra of **3a** have been added in the Section 6. NMR spectra. Based on the analysis of 2D spectra and ^{1}H, ^{13}C NMR spectra, the detailed assignments of ^{1}H and ^{13}C NMR signal have been summarized in the following table. To simplify the discussion, we numbered all carbon atoms in product **3a**, and different colors were used to distinguish the three main parts of the structure, and the atom 4 is an important position for the determination of the structure.

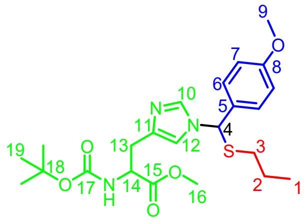

No.	^{1}H NMR chemical shift	^{13}C NMR chemical shift

1	0.9 (CH3)	13.4
2	1.6 (CH2)	22.2
3	2.3-2.5 (CH2)	33.9
4	6.1 (CH)	64.0
5	-	130.0
6	7.1 (Ar-CH)	127.7
7	6.8 (Ar-CH)	114.1
8	-	159.8
9	3.6 (CH3)	52.1
10	7.7 (Imidazole-CH)	136.5
11	-	138.1
12	6.9 (Imidazole-CH)	115.8
13	3.0 (CH2)	30.5
14	4.5 (CH)	53.6
15	-	172.5
16	3.8 (CH3)	55.3
17	-	155.5
18	-	79.6
19	1.4 (tCH3)	28.3

After the confirmation of the ^{1}H and ^{13}C NMR signal, the signal of the ^{1}H nuclear and remotely coupled ^{13}C nuclear from the ^{1}H-^{13}C HMBC (^{1}H detected heteronuclear multiple bond correlation) spectrum is important for the determination of the reacted position on imidazole. Two possible product structures and the ^{1}H-^{13}C HMBC signal of the atom 4 (^{1}H δ 6.1, ^{13}C δ 64.0) are shown in the following figure. As a remotely coupling NMR experiment, two different coupling signals would provide for the two possible structures. For the N3 reacted position, the atom 4 would couple with atom 10 (^{1}H δ 7.7, ^{13}C δ 136.5) and 12 (^{1}H δ 6.9, ^{13}C δ 115.8). For the N1 reacted position, the atom 4 would couple with atom 10 and 11 (^{13}C δ 138.1). Thus, we can determine its structure by the key signal from atom 11 or 12. Apparently, the coupling signal on {6.14, 115.77} ($^{1}H4 \rightarrow ^{13}C12$), {6.14, 136.70} ($^{1}H4 \rightarrow ^{13}C10$), {7.67, 64.01} ($^{1}H10 \rightarrow ^{13}C4$) and {6.97, 64.01} ($^{1}H12 \rightarrow ^{13}C4$) were found in the following HMBC spectrum. As a result, we confirmed that the reacted position on imidazole for this reaction is the N3 position.

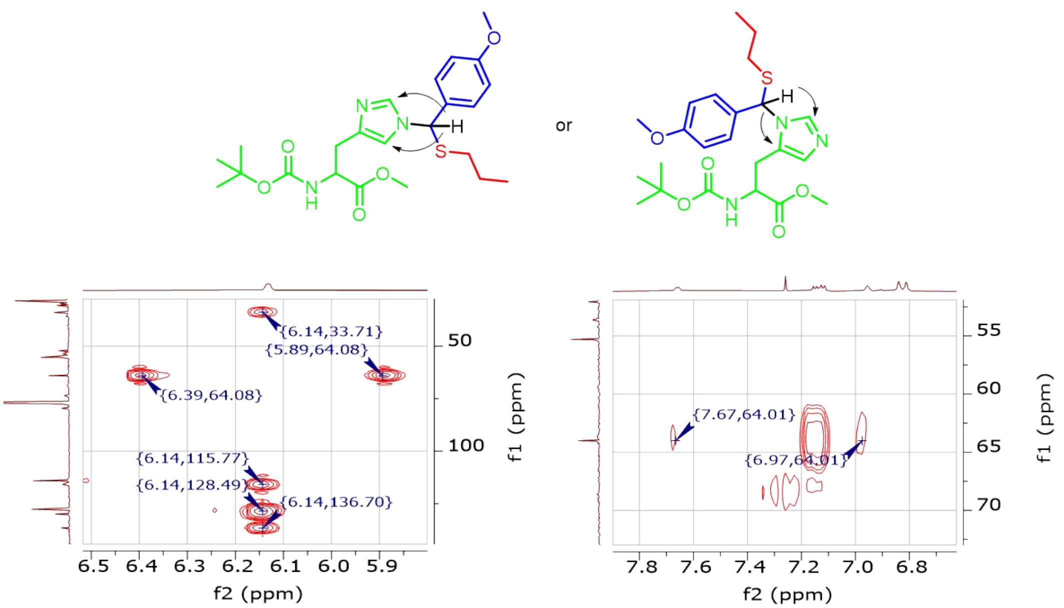

Synthesis of **3b**:

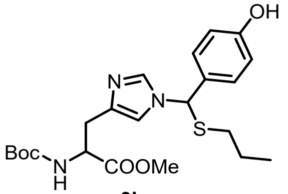

3b

(yellow oil, yield 86%) ^{1}H NMR (400 MHz, Chloroform-*d*) δ 7.70 (dd, *J* = 13.0, 1.1 Hz, 1H), 7.10 – 6.96 (m, 3H), 6.85 – 6.74 (m, 2H), 6.12 (d, *J* = 3.4 Hz, 1H), 5.71 (t, *J* = 8.1 Hz, 1H), 4.54 (q, *J* = 5.3 Hz, 1H), 3.61 (d, *J* = 7.5 Hz, 3H), 3.15 – 2.98 (m, 2H), 2.53 – 2.32 (m, 2H), 1.58 (dt, *J* = 14.3, 7.3 Hz, 2H), 1.41 (s, 9H), 0.98 (t, *J* = 7.3 Hz, 3H).^{13}C NMR (101 MHz, Chloroform-*d*) δ 172.49, 157.74, 155.72, 136.46, 128.77, 127.84, 116.51, 116.06, 80.05, 64.60, 53.75, 52.34, 34.10, 30.64, 28.42, 22.33, 13.56. HRMS: (ESI): $[M+H]^+$ Calcd. m/z 450.20572, found m/z 450.20605, Error: 0.75 ppm.

Synthesis of **3c**:

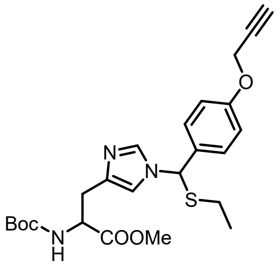

3c

(yellow oil, yield 79%) ^{1}H NMR (500 MHz, Chloroform-*d*) δ 7.67 (d, *J* = 3.9 Hz, 1H), 7.22 – 7.11 (m, 2H), 6.97 (s, 1H), 6.94 – 6.89 (m, 2H), 6.17 (d, *J* = 2.8 Hz, 1H), 5.87 (dd, *J* = 19.5, 8.4 Hz, 1H), 4.66 (d, *J* = 2.4 Hz, 2H), 4.55 (dt, *J* = 8.7, 5.0 Hz, 1H), 3.63 (s, 3H), 3.12 – 2.97 (m, 2H), 2.55 – 2.36 (m,

3H), 1.42 (d, J = 2.0 Hz, 9H), 1.24 – 1.23 (m, 3H). ^{13}C NMR (126 MHz, Chloroform-d) δ 157.79, 136.57, 130.89, 127.75, 115.87, 115.20, 79.66, 78.17, 75.86, 63.75, 55.87, 52.12, 30.59, 28.39, 26.04, 14.03. HRMS: (ESI): $[M+H]^+$ Calcd. m/z 474.20572, found m/z 474.20609, Error: 0.77 ppm.

Synthesis of **3d**:

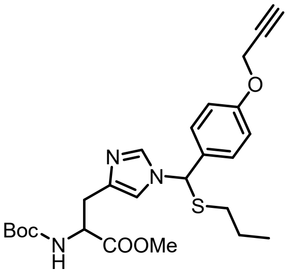

3d

(yellow oil, yield 81%) ^{1}H NMR (500 MHz, Chloroform-d) δ 7.66 (d, J = 6.9 Hz, 1H), 7.14 (dd, J = 8.6, 5.1 Hz, 2H), 6.95 (s, 1H), 6.90 (dd, J = 8.7, 1.7 Hz, 2H), 6.13 (d, J = 3.2 Hz, 1H), 5.86 (dd, J = 19.3, 8.3 Hz, 1H), 4.64 (d, J = 2.4 Hz, 2H), 4.53 (dt, J = 8.8, 5.2 Hz, 1H), 3.61 (s, 3H), 3.08 – 2.96 (m, 2H), 2.54 – 2.27 (m, 3H), 1.55 (dtt, J = 21.8, 11.5, 6.0 Hz, 2H), 1.39 (s, 9H), 0.94 (t, J = 7.3 Hz, 3H).^{13}C NMR (126 MHz, Chloroform-d) δ 172.47, 157.76, 155.58, 136.58, 136.57, 130.98, 127.72, 115.91, 115.17, 79.64, 78.17, 75.89, 64.03, 55.84, 53.70, 52.11, 33.97, 30.54, 28.37, 22.22, 13.46.HRMS: (ESI): $[M+H]^+$ Calcd. m/z 488.22137, found m/z 488.22284, Error: 3.01 ppm.

Synthesis of **3e**:

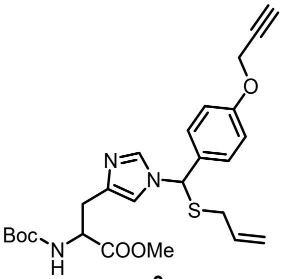

3e

(yellow oil, yield 62%) ^{1}H NMR (500 MHz, Chloroform-d) δ 7.72 – 7.59 (m, 1H), 7.19 – 7.10 (m, 2H), 6.98 – 6.89 (m, 3H), 6.22 – 6.06 (m, 1H), 5.93 – 5.83 (m, 1H), 5.82 – 5.73 (m, 1H), 5.18 (d, J = 10.1 Hz, 1H), 5.12 – 5.03 (m, 2H), 4.66 (t, J = 2.4 Hz, 2H), 4.59 – 4.52 (m, 1H), 3.63 (t, J = 3.4 Hz, 3H), 3.10 (dd, J = 11.3, 7.1 Hz, 2H), 2.61 – 2.53 (m, 2H), 2.51 – 2.49 (m, 1H), 1.42 (s, 9H). ^{13}C NMR (126 MHz, Chloroform-d) δ 155.60, 136.62, 134.26, 130.75, 127.77, 117.18, 115.84, 115.21, 79.66, 78.15, 75.89, 64.06, 55.86, 53.71, 52.14, 34.76, 30.69, 28.40. HRMS: (ESI): $[M+H]^+$ Calcd. m/z 486.20572, found m/z 486.20593, Error: 0.44ppm.

Synthesis of **3f**:

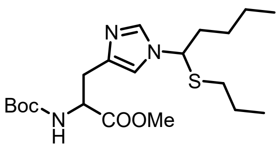

3f

(yellow oil, yield 47%) ^{1}H NMR (500 MHz, Chloroform-*d*) δ 7.59 – 7.46 (m, 1H), 6.91 – 6.79 (m, 1H), 4.85 (ddd, *J* = 73.0, 8.7, 6.1 Hz, 1H), 4.55 (s, 1H), 3.68 (d, *J* = 10.2 Hz, 3H), 3.13 – 2.95 (m, 2H), 2.12 – 1.97 (m, 2H), 1.78 – 1.61 (m, 4H), 1.43 (s, 9H), 1.28 (q, *J* = 10.8, 7.8 Hz, 4H), 0.98 – 0.92 (m, 3H), 0.89 – 0.85 (m, 3H). ^{13}C NMR (101 MHz, Chloroform-*d*) δ 114.79, 114.50, 79.82, 69.20, 53.69, 52.27, 36.49, 29.82, 28.62, 28.47, 28.17, 22.35, 22.12, 13.90, 13.53. HRMS: (ESI): $[M+H]^+$ Calcd. m/z 414.24210, found m/z 414.24228, Error: 0.42ppm.

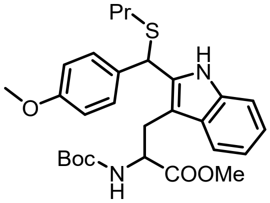

Boc-Trp-OMe-**1a**

(yellow oil, yield 89%) ^{1}H NMR (300 MHz, Chloroform-*d*) δ 8.93 (d, *J* = 34.6 Hz, 1H), 7.56 (d, *J* = 7.5 Hz, 1H), 7.50 – 7.30 (m, 3H), 7.22 – 7.05 (m, 2H), 6.94 – 6.76 (m, 2H), 5.54 (d, *J* = 2.2 Hz, 1H), 5.24 (dd, *J* = 11.7, 8.1 Hz, 1H), 4.66 (q, *J* = 6.0 Hz, 1H), 3.75 (s, 3H), 3.58 (s, 3H), 3.30 (dt, *J* = 13.7, 6.4 Hz, 2H), 2.42 (th, *J* = 20.1, 7.3, 6.4 Hz, 2H), 1.63 (dq, *J* = 14.3, 6.7, 4.8 Hz, 2H), 1.44 (d, *J* = 8.8 Hz, 9H), 0.99 (td, *J* = 7.3, 2.2 Hz, 3H). ^{13}C NMR (75 MHz, $CDCl_3$) δ 172.92, 158.93, 155.31, 135.56, 135.37, 131.62, 129.25, 128.45, 122.09, 119.66, 118.72, 114.18, 111.16, 107.63, 79.80, 55.20, 54.25, 52.27, 44.72, 34.43, 28.29, 27.34, 22.57, 13.58.

3.2 General procedure B for the synthesis of thioacetals and thioketals

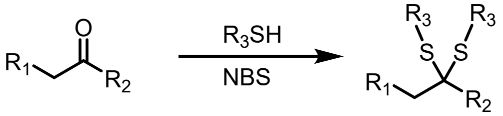

To a round-bottomed flask was charged with mercaptan (4 equiv.) and aldehydes/ketones (1 equiv.), and CH_2Cl_2 was added to dissolve the starting materials. A catalytic amount of N-Bromosuccinimide (NBS, 5 mol%) was added to the mixture. The solution was allowed to stir for 2 h at room temperature. After reaction completion, the organic phase was washed with saturated NaCl solution for two times and dried over anhydrous Na_2SO_4, and concentrated on a rotary evaporator. The crud product was purified by flash column chromatography using eluent solution Hexane/EtOAc or CH_2Cl_2/MeOH.

Synthesis of **1** followed General Procedure B using 4-methoxybenzaldehyde and propane-1-thiol as substrates:

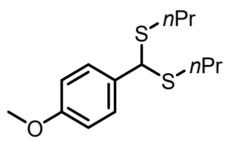

1a

(colorless oil, yield 95%) ^{1}H NMR (300 MHz, Chloroform-*d*) δ 7.47 – 7.30 (m, 2H), 6.96 – 6.78 (m, 2H), 4.87 (s, 1H), 3.79 (s, 3H), 2.52 (qt, *J* = 12.5, 7.3 Hz, 4H), 1.60 (dt, *J* = 14.6, 7.3 Hz, 4H), 0.96 (t, *J* = 7.3 Hz, 6H). ^{13}C NMR (75 MHz, $CDCl_3$) δ 159.05, 132.61, 128.83, 113.78, 55.24, 52.47, 34.29, 22.57, 13.55.

Synthesis of **TA3** followed General Procedure B using 4-(prop-2-yn-1-yloxy)benzaldehyde **S15** and ethanethiol as substrates:

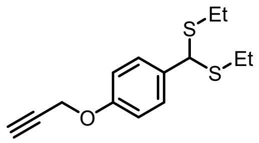

TA3

(light yellow oil, yield 93%) ^{1}H NMR (300 MHz, Chloroform-*d*) δ 7.47 – 7.30 (m, 2H), 7.02 – 6.82 (m, 2H), 4.90 (s, 1H), 4.66 (d, *J* = 2.3 Hz, 2H), 2.71 – 2.37 (m, 5H), 1.20 (t, *J* = 7.4 Hz, 6H). ^{13}C NMR (75 MHz, $CDCl_3$) δ 157.05, 133.43, 128.85, 114.78, 78.49, 75.67, 55.84, 51.75, 26.20, 14.30.

Synthesis of **TA4** followed General Procedure B using **S15** and propane-1-thiol as substrates:

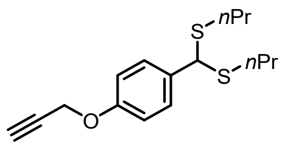

TA4

(light yellow oil, yield 91%) ^{1}H NMR (300 MHz, Chloroform-*d*) δ 7.35 (d, *J* = 8.7 Hz, 2H), 6.93 – 6.83 (m, 2H), 4.84 (s, 1H), 4.61 (d, *J* = 2.4 Hz, 2H), 2.60 – 2.36 (m, 5H), 1.53 (h, *J* = 7.1 Hz, 4H), 0.91 (t, *J* = 7.3 Hz, 6H). ^{13}C NMR (75 MHz, $CDCl_3$) δ 157.01, 133.59, 128.86, 114.72, 78.58, 75.76, 55.81, 52.48, 34.25, 22.55, 13.58.

Synthesis of **TA5** followed General Procedure B using **S15** and prop-2-ene-1-thiol as substrates:

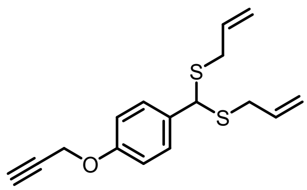

TA5

(light yellow oil, yield 82%) ^{1}H NMR (300 MHz, Chloroform-*d*) δ 7.42 – 7.30 (m, 2H), 6.99 – 6.85 (m, 2H), 5.85 – 5.72 (m, 2H), 5.13 – 5.06 (m, 4H), 4.74 (s, 1H), 4.67 (d, *J* = 2.4 Hz, 2H), 3.25 (dd, *J* = 13.7, 7.1 Hz, 2H), 3.03 (dd, *J* = 13.7, 7.2 Hz, 2H), 2.53 (dt, *J* = 4.5, 2.2 Hz, 1H). ^{13}C NMR (75 MHz, $CDCl_3$) δ 157.13, 133.81, 132.77, 129.23, 117.57, 114.85, 78.46, 75.68, 55.84, 49.68, 35.24.

Synthesis of **TA6** followed General Procedure B using **S15** and 2-mercaptoethan-1-ol as substrates:

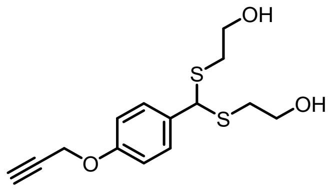

TA6

(white powder, yield 90%) ^{1}H NMR (300 MHz, Chloroform-*d*) δ 7.34 (d, *J* = 8.7 Hz, 2H), 6.89 (d, *J* = 8.7 Hz, 2H), 5.03 (s, 1H), 4.63 (d, *J* = 2.3 Hz, 2H), 3.66 (t, *J* = 6.0 Hz, 4H), 3.43 (s, 2H), 2.82 – 2.69 (m, 2H), 2.67 – 2.56 (m, 2H), 2.54 (t, *J* = 2.3 Hz, 1H). ^{13}C NMR (75 MHz, $CDCl_3$) δ 157.23, 132.92, 128.93, 114.97, 78.43, 75.98, 61.34, 55.88, 52.45, 35.10.

Synthesis of **TA7** followed General Procedure B using 4-formyl-N-(prop-2-yn-1-yl)benzamide and propane-1-thiol as substrates. The preparation of 4-formyl-N-(prop-2-yn-1-yl)benzamide was followed the same procedure as described in previous literature.[9]

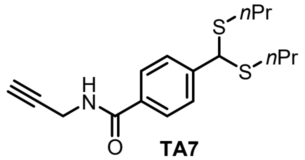

TA7

(yellow oil, yield 88%) ^{1}H NMR (300 MHz, Chloroform-*d*) δ 7.75 (d, *J* = 8.3 Hz, 2H), 7.46 (d, *J* = 8.2 Hz, 2H), 6.91 (t, *J* = 4.8 Hz, 1H), 4.84 (s, 1H), 4.19 (dd, *J* = 5.3, 2.5 Hz, 2H), 2.55 – 2.33 (m, 4H), 2.23 (t, *J* = 2.5 Hz, 1H), 1.53 (h, *J* = 7.2 Hz, 4H), 0.90 (t, *J* = 7.4 Hz, 6H). ^{13}C NMR (75 MHz, $CDCl_3$) δ 166.84, 144.64, 133.06, 127.91, 127.43, 79.59, 71.65, 52.57, 34.28, 29.67, 22.46, 13.47.

Synthesis of **S1** followed General Procedure B using 4-methoxybenzaldehyde and ethane-1,2-dithiol as substrates.

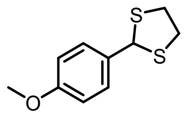

S1

(white powder, yield 94%) ^{1}H NMR (300 MHz, Chloroform-*d*) δ 7.56 – 7.35 (m, 2H), 6.97 – 6.73 (m, 2H), 5.63 (s, 1H), 3.79 (s, 3H), 3.50 (ddd, *J* = 7.0, 5.9, 3.9 Hz, 2H), 3.45 – 3.25 (m, 2H). ^{13}C NMR (75 MHz, $CDCl_3$) δ 159.36, 131.79, 129.15, 113.85, 56.04, 55.33, 40.22.

Synthesis of **S2** followed General Procedure B using **S15** and ethane-1,2-dithiol as substrates.

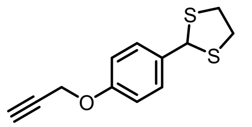

S2

(white powder, yield 92%) ^{1}H NMR (300 MHz, Chloroform-*d*) δ 7.52 – 7.41 (m, 2H), 6.97 – 6.86 (m, 2H), 5.62 (s, 1H), 4.65 (d, *J* = 2.4 Hz, 2H), 3.50 – 3.36 (m, 2H), 3.36 – 3.21 (m, 2H), 2.57 (t, *J* = 2.4 Hz, 1H). ^{13}C NMR (75 MHz, $CDCl_3$) δ 157.26, 133.03, 129.27, 114.84, 78.70, 75.96, 55.96, 55.88, 40.31.

Synthesis of **S3** followed General Procedure B using **S15** and propane-1,3-dithiol as substrates.

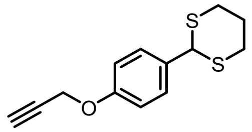

S3

(white powder, yield 90%) ^{1}H NMR (300 MHz, Chloroform-*d*) δ 7.40 (d, *J* = 8.6 Hz, 2H), 6.93 (d, *J* = 8.7 Hz, 2H), 5.13 (s, 1H), 4.67 (d, *J* = 2.3 Hz, 2H), 3.17 – 2.82 (m, 4H), 2.52 (t, *J* = 2.2 Hz, 1H), 2.26 – 2.09 (m, 1H), 2.01 – 1.81 (m, 1H). ^{13}C NMR (75 MHz, $CDCl_3$) δ 157.50, 132.22, 128.96, 115.02, 78.39, 75.64, 55.80, 50.66, 32.14, 25.04.

Synthesis of **S4** followed General Procedure B using **S15** and dropwise addition of 1 equivalent of 2-mercaptoethan-1-ol as substrates.

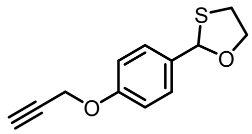

S4

(yellow oil, yield 49%) ^{1}H NMR (300 MHz, Chloroform-*d*) δ 7.48 – 7.36 (m, 2H), 7.02 – 6.91 (m, 2H), 6.00 (s, 1H), 4.51 (ddd, *J* = 9.2, 6.4, 2.9 Hz, 1H), 3.91 (td, *J* = 9.0, 6.2 Hz, 1H), 3.34 – 3.09 (m, 2H), 2.53 (t, *J* = 2.4 Hz, 1H). ^{13}C NMR (75 MHz, $CDCl_3$) δ 157.80, 132.00, 128.25, 114.77, 86.85, 78.43, 75.69, 71.79, 55.81, 34.08.

Synthesis of **S5** was followed General Procedure B using **S15** and dropwise addition of 1 equivalent of 2-aminoethane-1-thiol as substrates.

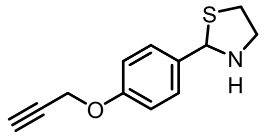

S5

(white powder, yield 69%) ^{1}H NMR (300 MHz, Chloroform-*d*) δ 7.50 – 7.38 (m, 2H), 7.01 – 6.88 (m, 2H), 5.50 (s, 1H), 4.67 (d, *J* = 2.4 Hz, 2H), 3.70 – 3.55 (m, 1H), 3.20 – 2.99 (m, 3H), 2.51 (t, *J* = 2.4 Hz, 1H). ^{13}C NMR (75 MHz, $CDCl_3$) δ 157.36, 132.86, 128.52, 114.84, 78.43, 75.63, 72.92, 55.82, 52.75, 36.53.

Synthesis of **S6** was followed General Procedure B using 1-(4-methoxyphenyl)ethan-1-one and propane-1-thiol as substrates.

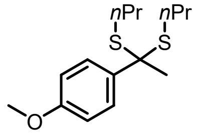

S6

(light yellow oil, yield 63%) ^{1}H NMR (300 MHz, Chloroform-*d*) δ 7.74 – 7.55 (m, 2H), 6.96 – 6.80 (m, 2H), 3.81 (s, 3H), 2.49 (t, *J* = 7.4 Hz, 4H), 2.02 (s, 3H), 1.54 (h, *J* = 7.4 Hz, 4H), 0.95 (t, *J* = 7.4 Hz, 6H). ^{13}C NMR (75 MHz, $CDCl_3$) δ 158.47, 136.02, 128.24, 113.28, 59.81, 55.21, 32.90, 30.33, 22.37, 13.84.

Synthesis of **S7** was followed General Procedure B using 1-(4-(prop-2-yn-1-yloxy)phenyl)ethan-1-one **S16** and propane-1-thiol as substrates.

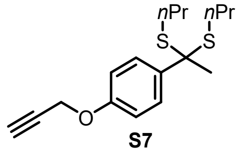

S7

(light yellow oil, yield 61%) ^{1}H NMR (300 MHz, Chloroform-*d*) δ 7.78 – 7.49 (m, 2H), 7.05 – 6.79 (m, 2H), 4.67 (d, *J* = 2.4 Hz, 2H), 2.46 (t, *J* = 7.4 Hz, 4H), 2.18 (s, 1H), 1.98 (s, 3H), 1.51 (q, *J* = 7.3 Hz, 4H), 0.93 (t, *J* = 7.4 Hz, 6H). ^{13}C NMR (75 MHz, $CDCl_3$) δ 156.46, 136.99, 128.26, 114.21, 78.49, 75.57, 59.77, 55.78, 32.88, 30.29, 22.33, 13.83.

3.3 Synthesis of TA8 and TA9

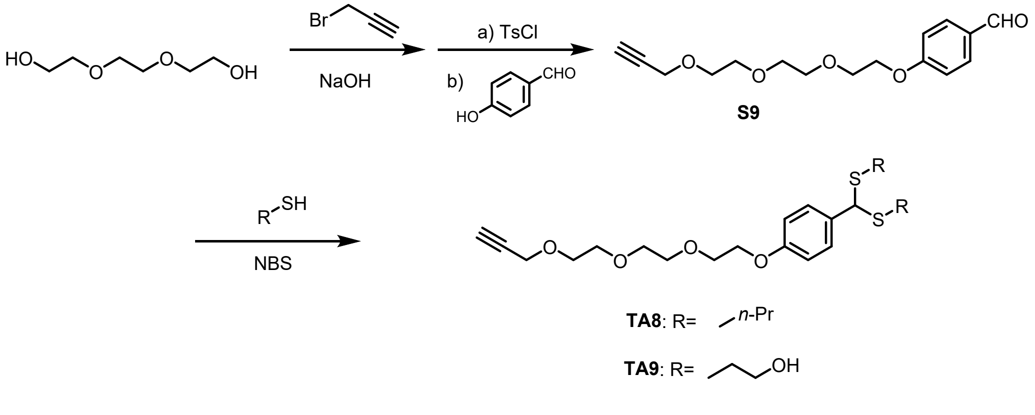

To a round-bottomed flask was added 2,2'-(ethane-1,2-diylbis(oxy))bis(ethan-1-ol) (1.5 g, 10 mmol) dissolved in 50 mL THF, and cooled in an ice bath. NaOH (0.8 g, 20 mmol) was added in one portion, and 3-bromoprop-1-yne (1.18 g, 10 mmol) was added dropwise after 1h. The solution was allowed to warm to room temperature and stirred for 16 h. After reaction completion, the solvent was removed, and then 20 mL H_2O was added. The aqueous solution was neutralized with 1 M dilute HCl, and extracted with CH_2Cl_2 (3×20 mL). The organic phase was washed with saturated NaCl solution (2×10 mL) and dried over anhydrous Na_2SO_4. The crud product was purified by flash column chromatography using eluent solution Hexane/EtOAc (1:1). The purified intermediate was used directly for next step. The intermediate 2-(2-(2-(prop-2-yn-1-yloxy)ethoxy)ethoxy)ethan-1-ol was dissolved with 4-methylbenzenesulfonyl chloride (TsCl, 1.9 g, 10 mmol) and Et_3N (1.01g , 10 mmol) in 50 mL CH_2Cl_2. The solution was allowed to stir for 2 h. 4-hydroxybenzaldehyde (1.22 g, 10 mmol) and addition Et_3N (1.01g , 10 mmol) were added in one portion, and the reaction was continued for 16 h. After reaction completion, the solvent was removed, and then 50 mL H_2O was added. The crud mixture was extracted with CH_2Cl_2 (3×20 mL). The organic phase was washed with saturated NaCl solution (2×10 mL) and dried over anhydrous Na_2SO_4. The crud product was purified by flash column chromatography using eluent solution Hexane/EtOAc (2:1).

S9 (colorless oil, yield 64% for two steps)**:** ^{1}H NMR (500 MHz, Chloroform-*d*) δ 9.83 (s, 1H), 7.83 – 7.73 (m, 2H), 7.04 – 6.92 (m, 2H), 4.17 (dd, *J* = 5.4, 4.3 Hz, 2H), 4.15 (d, *J* = 2.4 Hz, 2H), 3.84 (dd, *J* = 5.4, 4.1 Hz, 2H), 3.71 – 3.67 (m, 2H), 3.64 (tdd, *J* = 4.3, 2.7, 0.9 Hz, 6H), 2.40 (t, *J* = 2.4 Hz, 1H). ^{13}C NMR (126 MHz, Chloroform-*d*) δ 190.82, 163.87, 131.96, 130.05, 114.91, 79.66, 74.64, 70.89, 70.66, 70.47, 69.48, 69.11, 67.79, 58.41.

The synthesis of **TA8** and **TA9** were followed General Procedure B using **S9** and propane-1-thiol/2-mercaptoethan-1-ol as substrates.

TA8 (colorless oil, yield 91%)**:** ^{1}H NMR (500 MHz, Chloroform-*d*) δ 7.38 – 7.29 (m, 2H), 6.91 – 6.78 (m, 2H), 4.81 (s, 1H), 4.17 (d, *J* = 2.5 Hz, 2H), 4.11 – 4.07 (m, 2H), 3.84 – 3.80 (m, 2H), 3.70 (ddt, *J* = 6.5, 3.6, 2.0 Hz, 2H), 3.67 – 3.65 (m, 6H), 2.54 – 2.41 (m, 4H), 2.40 (d, *J* = 2.4 Hz, 1H), 1.54 (pd, *J* = 7.7, 1.0 Hz, 4H), 0.91 (t, *J* = 7.3 Hz, 6H). ^{13}C NMR (126 MHz, Chloroform-*d*) δ 158.31, 132.85, 128.85, 114.53, 79.69, 74.59, 70.85, 70.69, 70.48, 69.74, 69.13, 67.47, 58.43, 52.51, 34.33, 22.60, 13.58.

TA9 (colorless oil, yield 83%)**:** ^{1}H NMR (400 MHz, Chloroform-*d*) δ 7.34 (d, *J* = 8.4 Hz, 2H), 6.85 (d, *J* = 8.4 Hz, 2H), 5.04 (s, 1H), 4.22 – 4.13 (m, 2H), 4.08 (d, *J* = 4.4 Hz, 2H), 3.88 – 3.76 (m, 2H), 3.67 (q, *J* = 5.4 Hz, 10H), 3.19 (t, *J* = 38.8 Hz, 2H), 2.78 (dq, *J* = 12.0, 6.8, 5.7 Hz, 2H), 2.63 (dt, *J* = 13.1, 5.8 Hz, 2H), 2.46 (s, 1H). ^{13}C NMR (101 MHz, Chloroform-*d*) δ 158.52, 132.26, 128.94, 114.72, 79.67, 74.84, 70.75, 70.60, 70.39, 69.67, 69.08, 67.47, 61.38, 58.40, 52.54, 35.21.

3.4 Synthesis of TA1 and TA10

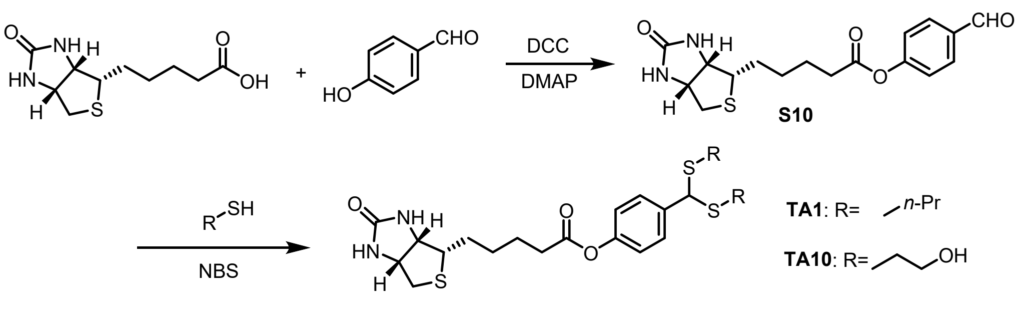

4-Dimethylaminopyridine (DMAP, 48 mg, 0.4 mmol) was added to D-Biotin solution (488 mg, 2 mmol) in 100 mL CH_2Cl_2, followed by dicyclohexylcarbodiimide (DCC, 496 mg, 2.4 mmol). After stirring for l h, a solution of 4-hydroxybenzaldehyde (268 mg, 2.2 mmol) in CH_2Cl_2 (20 mL) was added and stirred at room temperature for 16 h. The crystals were filtered through celite and washed on the filter with CH_2Cl_2 (100 mL). The combined solution was washed successively with saturated Na_2CO_3(aq) and brine, then dried over Na_2SO_4. The solvent was then removed in vacuum to afford the crude product, which was purified by silica gel chromatography with CH_2Cl_2/CH_3OH (40:1) to furnish the product.

S10 (white solid, yield 72%): ^{1}H NMR (300 MHz, DMSO-d_6) δ 9.94 (s, 1H), 8.14 – 7.83 (m, 2H), 7.32 (d, *J* = 8.5 Hz, 2H), 4.32 (dd, *J* = 7.7, 4.6 Hz, 1H), 4.17 – 4.13 (m, 1H), 3.17 – 3.06 (m, 1H), 2.80 (dd, *J* = 12.5, 4.9 Hz, 1H), 2.64 – 2.54 (m, 3H), 1.69 – 1.58 (m, 3H), 1.52 – 1.36 (m, 3H).

^{13}C NMR (75 MHz, DMSO) δ 192.66, 171.98, 163.50, 155.50, 134.19, 131.57, 123.18, 61.59, 59.75, 55.75, 40.22, 33.68, 28.34, 24.60.

The synthesis of **TA1** and **TA10** were followed General Procedure B using **S10** and propane-1-thiol/2-mercaptoethan-1-ol as substrates.

TA1 (white solid, yield 86%): ^{1}H NMR (300 MHz, Methanol-d_4) δ 7.52 – 7.42 (m, 2H), 7.10 – 7.01 (m, 2H), 4.98 (s, 1H), 4.48 (dd, *J* = 7.8, 4.3 Hz, 1H), 4.30 (dd, *J* = 7.9, 4.5 Hz, 1H), 3.27 – 3.15 (m, 1H), 3.03 – 2.76 (m, 2H), 2.64 – 2.43 (m, 6H), 1.77 (ddd, *J* = 15.3, 7.4, 4.6 Hz, 3H), 1.70 – 1.52 (m, 7H), 0.94 (t, *J* = 7.4 Hz, 6H).

^{13}C NMR (75 MHz, MeOD) δ 180.18, 172.35, 164.68, 150.12, 138.71, 128.56, 121.31, 61.98, 60.21, 55.57, 51.91, 39.72, 33.93, 33.42, 29.21, 28.30, 24.46, 22.23, 12.46.

TA10 (white solid, yield 81%): ^{1}H NMR (300 MHz, Methanol-d_4) δ 7.51 (d, *J* = 8.6 Hz, 2H), 7.08 (d, *J* = 8.6 Hz, 2H), 4.45 (dd, *J* = 7.7, 4.8 Hz, 1H), 4.27 (dd, *J* = 7.8, 4.4 Hz, 1H), 3.77 – 3.57 (m, 5H), 3.20 (dt, *J* = 9.5, 5.3 Hz, 1H), 2.90 (dd, *J* = 12.8, 4.8 Hz, 1H), 2.70 (ddt, *J* = 35.0, 14.5, 7.3 Hz, 7H), 1.81 – 1.45 (m, 6H).

^{13}C NMR (75 MHz, MeOD) δ 172.45, 164.67, 150.26, 138.45, 128.74, 121.55, 62.01, 61.07, 60.23, 55.63, 52.36, 39.86, 34.37, 33.53, 28.37, 24.52.

3.5 Synthesis of TA2 and TA11

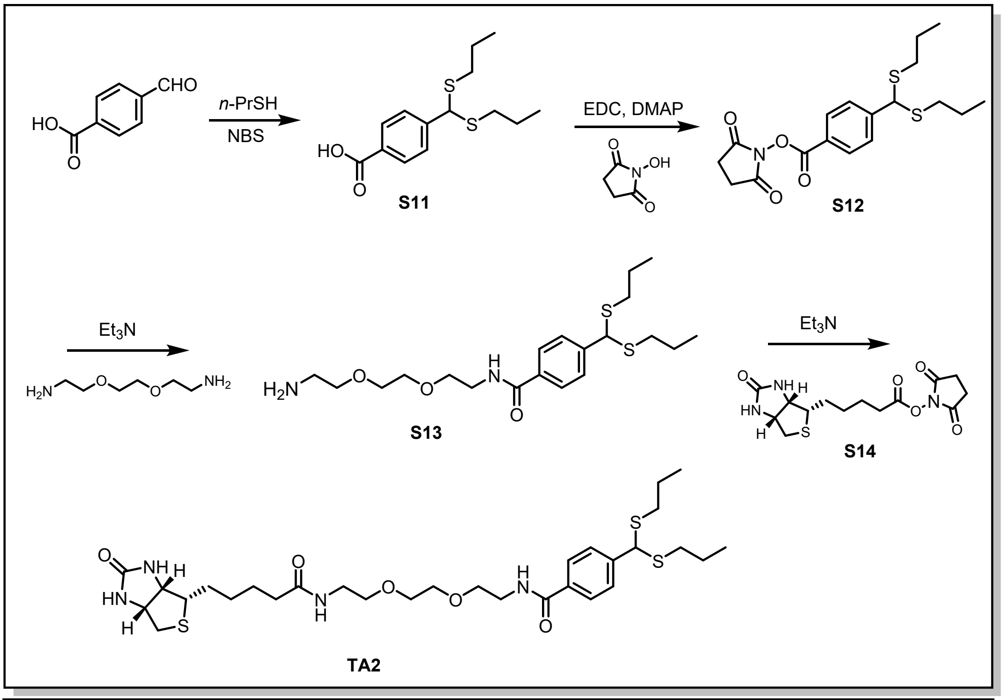

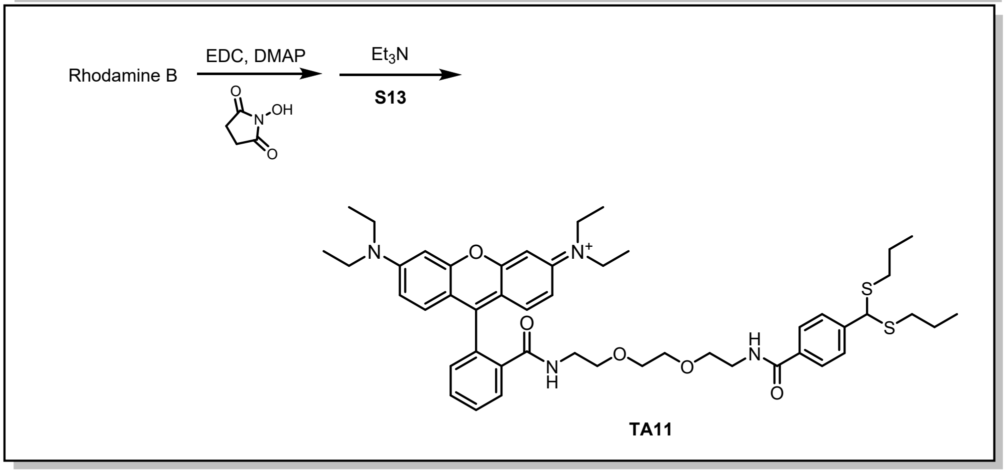

The synthesis of **S11** was followed General Procedure B using 4-formylbenzoic acid and propane-1-thiol as substrates.

S11 (white solid, yield 89%): ^{1}H NMR (400 MHz, Chloroform-*d*) δ 8.10 (d, *J* = 8.3 Hz, 2H), 7.57 (d, *J* = 8.3 Hz, 2H), 4.92 (s, 1H), 2.71 – 2.40 (m, 4H), 1.60 (h, *J* = 7.2 Hz, 4H), 0.97 (t, *J* = 7.3 Hz, 6H). ^{13}C NMR (101 MHz, Chloroform-*d*) δ 171.83, 147.12, 130.66, 128.77, 128.01, 52.90, 34.46, 22.64, 13.62.

DMAP (0.12 g, 1 mmol) was added to **S11** (1.42 g, 5 mmol) in 150 mL CH_2Cl_2, followed by 1-(3-Dimethylaminopropyl)-3-ethylcarbodiimide hydrochloride (EDC, 1.92 g, 10 mmol). After stirring for 10 min, 1-hydroxypyrrolidine-2,5-dione (NHS, 0.67 g, 5.5 mmol) was added and stirred at room temperature for 16 h. The solution was washed successively with brine for 3 times, then dried over Na_2SO_4. The solvent was then removed in vacuum to afford the crude product, which was recrystal by Hexane/EtOAc to purify the product.

S12 (white solid, yield 82%): ^{1}H NMR (400 MHz, Chloroform-*d*) δ 8.01 (d, *J* = 8.4 Hz, 2H), 7.51 (d, *J* = 8.3 Hz, 2H), 4.84 (s, 1H), 2.78 (s, 4H), 2.52 – 2.32 (m, 4H), 1.45 (hept, *J* = 6.8 Hz, 4H), 0.84 (t, *J* = 7.4 Hz, 6H). ^{13}C NMR (101 MHz, Chloroform-*d*) δ 169.59, 161.57, 148.53, 130.87, 128.40, 124.38, 52.57, 34.35, 25.79, 22.55, 13.54.

To a round-bottomed flask was added 2,2'-(ethane-1,2-diylbis(oxy))bis(ethan-1-amine) (1.78 g, 12 mmol) and Et_3N (0.41 g, 4 mmol) dissolved in 100 mL MeCN. **S12** (1.53 g, 4 mmol) in 20 mL MeCN solution was added dropwise. The solution was allowed to stir for 16 h. After reaction completion, the solvent was removed, and then 50 mL H_2O was added. The aqueous solution was extracted with CH_2Cl_2 (3×50 mL). The organic phase was washed with saturated NaCl solution (2×50 mL) and dried over anhydrous Na_2SO_4. The crud product was purified by flash column chromatography using eluent solution CH_2Cl_2/MeOH (30:1).

S13 (colorless oil, yield 61%): ^{1}H NMR (500 MHz, Chloroform-*d*) δ 7.76 (d, *J* = 8.2 Hz, 2H), 7.45 (d, *J* = 8.2 Hz, 2H), 7.18 (s, 1H), 4.84 (s, 1H), 3.68 – 3.53 (m, 8H), 3.46 (t, *J* = 5.1 Hz, 2H), 2.80 (t, *J* = 5.0 Hz, 2H), 2.57 – 2.38 (m, 4H), 2.17 (s, 2H), 1.59 – 1.46 (m, 4H), 0.90 (t, *J* = 7.3 Hz, 6H). ^{13}C NMR (126 MHz, Chloroform-*d*) δ 167.17, 144.24, 133.99, 127.83, 127.43, 72.85, 70.27, 70.10, 69.89, 52.67, 41.49, 39.80, 34.34, 22.52, 13.52.

Synthesis of NHS-Biotin **S14** was followed the same procedure of as described by previous literature.[10]

S14 (white solid, yield 91%): ^{1}H NMR (300 MHz, DMSO-d_6) δ 6.42 (s, 1H), 6.36 (s, 1H), 4.34 – 4.24 (m, 1H), 4.19 – 4.07 (m, 1H), 3.13 – 3.05 (m, 1H), 2.89 – 2.80 (m, 1H), 2.79 (s, 4H), 2.70 (d, *J* = 3.1 Hz, 1H), 2.65 (t, *J* = 7.4 Hz, 2H), 1.73 – 1.44 (m, 4H), 1.44 – 1.30 (m, 2H). ^{13}C NMR (75 MHz, DMSO) δ 170.73, 169.39, 163.15, 61.43, 59.61, 55.67, 30.42, 28.26, 28.02, 25.87, 25.65, 24.74.

To a round-bottomed flask was added **S13** (0.41 g, 1 mmol), NHS-Biotin **S14** (0.34 g, 1 mmol) and Et_3N (0.1 g, 1 mmol) dissolved in 50 mL MeCN. The solution was allowed to stir for 16 h. After reaction completion, the solvent was removed, and then 50 mL H_2O was added. The aqueous solution was extracted with CH_2Cl_2 (3×50 mL). The organic phase was washed with saturated NaCl solution (2×50 mL) and dried over anhydrous Na_2SO_4. The crud product was purified by flash column chromatography using eluent solution CH_2Cl_2/MeOH (40:1).

TA2 (colorless oil, yield 89%): ^{1}H NMR (500 MHz, Chloroform-*d*) δ 7.77 (d, *J* = 8.2 Hz, 2H), 7.45 (d, *J* = 8.4 Hz, 2H), 7.31 (t, *J* = 4.8 Hz, 1H), 6.81 (t, *J* = 5.1 Hz, 1H), 6.72 (s, 1H), 5.99 (s, 1H), 4.84 (s, 1H), 4.47 – 4.38 (m, 1H), 4.28 – 4.18 (m, 1H), 3.68 – 3.53 (m, 8H), 3.49 (dt, *J* = 10.9, 5.3 Hz, 2H), 3.36 (dtt, *J* = 14.3, 8.8, 4.1 Hz, 2H), 3.07 (q, *J* = 7.3 Hz, 1H), 2.82 (dd, *J* = 12.8, 4.7 Hz, 1H), 2.72 – 2.61 (m, 2H), 2.55 – 2.40 (m, 4H), 2.15 (t, *J* = 7.6 Hz, 2H), 1.72 – 1.48 (m, 8H), 1.36 (p, *J* = 7.3 Hz, 2H), 0.91 (t, *J* = 7.3 Hz, 6H). ^{13}C NMR (126 MHz, Chloroform-*d*) δ 173.63, 167.27, 164.47, 144.37, 133.82, 127.86, 127.47, 70.17, 70.05, 69.89, 61.87, 60.31, 55.73, 52.72, 40.48, 39.89, 39.12, 35.95, 34.38, 28.29, 28.08, 25.64, 25.49, 22.54, 13.54. HRMS: (ESI): $[M+H]^+$ Calcd. m/z 641.28596, found m/z 641.28638, Error: 0.65 ppm.

DMAP (0.048 g, 0.4 mmol) was added to Rhodamine B (0.96 g, 2 mmol) in 150 mL CH_2Cl_2, followed by 1-(3-Dimethylaminopropyl)-3-ethylcarbodiimide hydrochloride (EDC, 0.77 g, 4 mmol). After

stirring for 10 min, 1-hydroxypyrrolidine-2,5-dione (NHS, 0.27 g, 2.2 mmol) was added and stirred at room temperature for 16 h. The solution was washed successively with brine for 3 times, then dried over Na_2SO_4. The solvent was then removed in vacuum to afford the crude product, which was directly used for next step.

To a round-bottomed flask was added **S13** (0.41 g, 1 mmol), the crude intermediate NHS-Rhodamine B and Et_3N (0.1 g, 1 mmol) dissolved in 50 mL MeCN. The solution was allowed to stir for 16 h. After reaction completion, the solvent was removed, and then 50 mL H_2O was added. The aqueous solution was extracted with CH_2Cl_2 (3×50 mL). The organic phase was washed with saturated NaCl solution (2×50 mL) and dried over anhydrous Na_2SO_4. The crud product was purified by flash column chromatography using eluent solution CH_2Cl_2/MeOH (40:1).

TA11 (red oil, yield 51% for two steps): ^{1}H NMR (500 MHz, Chloroform-*d*) δ 7.88 – 7.83 (m, 1H), 7.79 (d, *J* = 8.4 Hz, 2H), 7.45 (d, *J* = 8.4 Hz, 2H), 7.42 – 7.38 (m, 2H), 7.23 (t, *J* = 4.9 Hz, 1H), 7.06 – 7.01 (m, 1H), 6.49 – 6.39 (m, 4H), 6.29 (dd, *J* = 8.9, 2.4 Hz, 2H), 4.84 (s, 1H), 3.57 (p, *J* = 4.7 Hz, 4H), 3.46 (dd, *J* = 5.5, 3.5 Hz, 2H), 3.37 – 3.27 (m, 12H), 3.13 (t, *J* = 7.0 Hz, 2H), 2.52 – 2.42 (m, 4H), 1.55 – 1.50 (m, 4H), 1.12 (t, *J* = 7.1 Hz, 12H), 0.92 – 0.89 (m, 6H). ^{13}C NMR (126 MHz, Chloroform-*d*) δ 168.44, 167.11, 153.61, 153.18, 148.05, 144.07, 133.98, 132.59, 130.83, 128.99, 128.20, 127.93, 127.86, 127.51, 127.35, 123.80, 122.83, 108.85, 98.96, 70.33, 69.99, 67.93, 64.85, 52.70, 45.08, 39.91, 39.44, 34.31, 22.54, 13.53, 12.44. HRMS: (ESI): [M]+ Calcd. m/z 839.42344, found m/z 839.42407, Error: 0.75 ppm.

3.6 Synthesis of tert-butyl ((ethylthio)(4-methoxyphenyl)methyl)carbamate S8

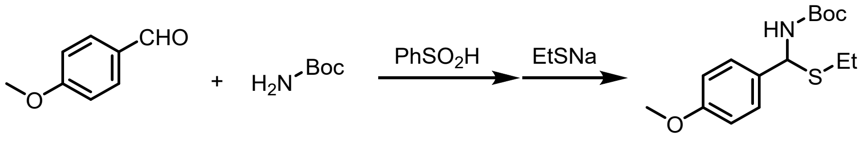

S8

Synthesis of **S8** was followed the same procedure as described by previous literature.[11]
S8 (white solid, yield 62%) ^{1}H NMR (500 MHz, Chloroform-*d*) δ 7.30 (d, *J* = 8.4 Hz, 2H), 6.83 (d, *J* = 8.7 Hz, 2H), 5.95 (d, *J* = 9.5 Hz, 1H), 5.26 (s, 1H), 3.75 (s, 3H), 2.77 – 2.47 (m, 2H), 1.42 (s, 9H), 1.29 (t, *J* = 7.3 Hz, 3H). ^{13}C NMR (126 MHz, Chloroform-*d*) δ 159.27, 154.69, 132.15, 127.61, 114.00, 80.03, 57.63, 55.30, 28.34, 25.57, 14.81.

3.7 Synthesis of intermediates

Synthesis of intermediate 4-(prop-2-yn-1-yloxy)benzaldehyde **S15**

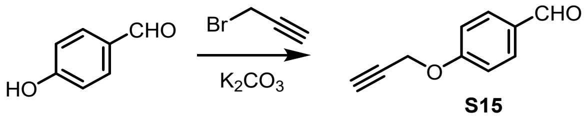

S15

4-hydroxybenzaldehyde (12.2g, 100 mmol) and 3-bromoprop-1-yne (11.8g, 100 mmol) were dissolved in 300 mL EtOH. Anhydrous K_2CO_3 (13.8g, 100 mmol) was added in one portion. The mixture was allowed to heated to reflux in oil bath for 6 h. After reaction completion, the solvent

was removed, and then 500 mL H_2O was added. The aqueous solution was extracted with EtOAc (3×100 mL). The organic phase was washed with 0.1 M HCl (2×100 mL) and saturated NaCl solution (2×100 mL) and dried over anhydrous Na_2SO_4. The solvent was then removed in vacuum to afford the crude product, which was recrystal by Hexane/EtOAc to purify the product.

S15 (white solid, yield 87%): ^{1}H NMR (300 MHz, Chloroform-*d*) δ 9.84 (s, 1H), 7.95 – 7.67 (m, 2H), 7.16 – 6.94 (m, 2H), 4.73 (d, *J* = 2.4 Hz, 2H), 2.57 (t, *J* = 2.4 Hz, 1H). ^{13}C NMR (75 MHz, $CDCl_3$) δ 190.85, 162.34, 131.87, 130.48, 115.14, 77.55, 76.44, 55.91.

Synthesis of intermediate 1-(4-(prop-2-yn-1-yloxy)phenyl)ethan-1-one **S16**

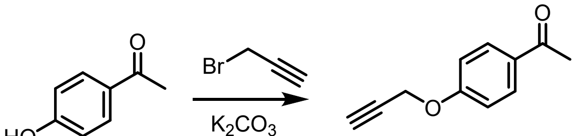

S16

The preparation of **S16** was followed the same procedure of the synthesis of **S15** using 1-(4-hydroxyphenyl)ethan-1-one and 3-bromoprop-1-yne as substrates.

S16 (white solid, yield 88%): ^{1}H NMR (300 MHz, Chloroform-*d*) δ 8.00 – 7.82 (m, 2H), 7.06 – 6.90 (m, 2H), 4.73 (d, *J* = 2.4 Hz, 2H), 2.55 (t, *J* = 2.4 Hz, 1H), 2.53 (s, 3H). ^{13}C NMR (75 MHz, $CDCl_3$) δ 196.80, 161.25, 130.95, 130.49, 114.53, 77.73, 76.19, 55.80, 26.34.

Synthesis of thioacetal 4-(bis(propylthio)methyl)phenol **S17**

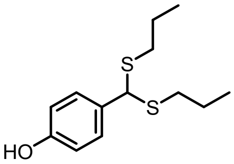

S17

Synthesis of **S17** was followed General Procedure B using 4-hydroxybenzaldehyde and propane-1-thiol as substrates. (colorless oil, yield 93%) ^{1}H NMR (400 MHz, Chloroform-*d*) δ 7.36 – 7.24 (m, 2H), 6.84 – 6.74 (m, 2H), 4.86 (s, 1H), 2.61 – 2.42 (m, 4H), 1.57 (h, *J* = 7.2 Hz, 4H), 0.94 (t, *J* = 7.3 Hz, 6H). ^{13}C NMR (101 MHz, Chloroform-*d*) δ 155.41, 132.58, 129.19, 115.61, 52.63, 34.48, 22.68, 13.68.

Synthesis of **S17** was followed General Procedure B using pentanal and propane-1-thiol as substrates.

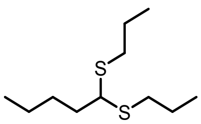

S18

(colorless oil, yield 94%) ^{1}H NMR (400 MHz, Chloroform-*d*) δ 3.73 (t, *J* = 7.0 Hz, 1H), 2.70 – 2.49 (m, 4H), 1.82 – 1.74 (m, 2H), 1.61 (qd, *J* = 7.4, 3.2 Hz, 4H), 1.55 – 1.46 (m, 2H), 1.35 – 1.29 (m, 2H),

1.00 (t, *J* = 7.3 Hz, 6H), 0.91 (t, *J* = 7.3 Hz, 3H). ^{13}C NMR (101 MHz, Chloroform-*d*) δ 52.07, 36.04, 32.23, 29.81, 22.94, 22.40, 14.07, 13.78.

4. Supplementary material and methods for the reactions of peptide

4.1 General information

All chemical reagents are commercially available without purification. **High-Resolution Mass Spectrometry (HRMS) and MS/MS Spectrometry** were measured on a Q_Exactive_Focus.

General procedure for SPPS: Peptide **7** and **8** were synthesized on Rink Amide MBHA resin (peptide **7**) or Fmoc-Wang resin (peptide **8**) by Fmoc solid-phase synthesis (SPPS). Rink-amide resin was pre-swelled with DCM for 30min, filtered, the Fmoc (9-fluorenylmethyloxycarbonyl) group was removed with 50% (vol/vol) morpholine for 30min*2; the resin was sequentially washed with DCM and DMF for three times. Fmoc-protected amino acids (2.0 equiv.) and HATU (2.0 equiv.) were dissolved in DMF, followed by DIPEA (3.0 equiv.). The mixture was pre-activated for 1min and added to the resin for 1 h with N_2 bubbling, repeated once. The resin was washed sequentially with DCM, DMF for three times, then dried under a stream of nitrogen for next step. For cleavage of resin, the final resin was treated with TFA/TIS/water (95:2.5:2.5) at room temperature for 3 h and concentrated under a stream of nitrogen. The crude peptides were precipitated and washed with cold hexane/diethyl ether (1:2, v/v) at 4°C, redissolved in 50% acetonitrile in water. Crude peptides were purified by preparative HPLC.

Acetamidation of peptide 8: A solution of un-protected peptide **8** (5.0 mg, 2 mM, PBS buffer 7.4 with 50% MeCN) was added iodoacetamide (IAM, 20 mM), and the resulted mixture was shaken in an incubator shaker (37 ^{o}C) for 1 hours. The crude reaction mixture was directly purified by preparative HPLC after filtration. 4.0 mg (78%) white powder was obtained as target product.

4.2 General procedure C for the reaction between peptide and thioacetal

A 10 mM MeCN/H_2O (1/1) stock solution of RB and a 200 mM MeCN stock solution of thioacetal was made up. These stock solutions were stored at room temperature away from light. To a 2 mL vial was added 100 uL solution of specific peptide (1 mM, in MeCN/H_2O=4/1) solvent and 2 µL RB (10 mM), 10 µL specific thioacetal (200 mM) stock solution. The vial was then caped and equipped with magnetic bar. The reaction was set in a reaction chamber equipped with magnetic stirrer, 450 nm LED lamp (10 W) and exhaust fan to maintain the reaction temperature at about 30 ^{o}C for 1 hour, as shown in the following figure. The resulting solution was then analyzed with an internal standard (dibenzyl sulfoxide, 0.2 mM) via HPLC-MS after filtration. The distillates of target products were collected and further analyzed by HRMS and MS/MS spectrometry. Desired distillates of preparative LC were identified by MS and lyophilized to obtain target products, and analyzed by NMR. Liquid nitrogen was used to quickly freeze distillates and lyophilize them as quickly as possible using a lyophilizer.

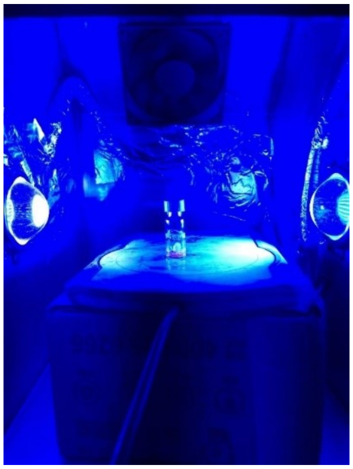

LC-MS yields were estimated by UV absorption at 220 nm of the peak corresponding to the thioacetal adducted product versus the internal standard (dibenzyl sulfoxide): % yield = (A_p/A_{st}) × *k*. A_p is the peak area of thioacetal-adducted products; A_{st} is the peak area of the internal standard; *k* is the quantity coefficient between specific peptide and standard. The quantity coefficients were measured by the correlation of a gradient concentration. The isolated yield of **4a** was obtained by a 10 mg level reaction, and isolated by a preparative LC. Note that the lyophilization of desired distillates need to be quick due to the potential hydrolyzation of thioacetal adducted products in weak acidic solution.

General method for LC/MS analysis

Analytical LC-MS were performed on a Shimadzu LC-MS 8030 system equipped with Kromasil 100-5-C18 column (4.6 × 250 mm, 5 μm; room temperature). Water (containing 0.1% TFA, A phase) and pure CH_3CN (B phase) were used as solvents in linear gradient mixtures at a flow rate of 1 mL·min^{-1}.

General method for preparative LC

Preparative LC were performed on a Shimadzu LC-6AD system equipped with Shimadzu Shim-pack GIST C18 column (20 × 250 mm, 5 μm; room temperature). Water (containing 0.1% TFA) and pure CH_3CN were used as solvents in linear gradient mixtures at a flow rate of 8 mL·min^{-1}. Due to the potential hydrolyzation of thioacetal adducted products in weak acidic solution (**Figure S6**), we tried a mobile phase of pure water to separate the products, and found that 0.1% TFA in water is essential. Thus, we tried quick freezing samples using liquid nitrogen and lyophilizing.

General method for MS/MS analysis

The fragmentation of modally modified peptides was investigated in positive electrospray ionization mode, loaded onto a Thermo Q Exactive Focus Orbitrap LC-MS/MS system. The protonated molecule was generated by spraying a 0.5 ng/μl solution in 20:80 water:methanol + 0.1% formic acid (FA) with a flow rate of 0.28 mL/min. Parameters are as follows in Full MS/ data dependent-MS2 TopN mode: mass analyzer over m/z range of 145−2175 with a mass resolution of 70,000 (at m/z=200) in a data-dependent mode. MS/MS spectra were obtained using collision energy values at 25% normalized activation energy with a HCD (High Energy Collision Dissociation) mode.

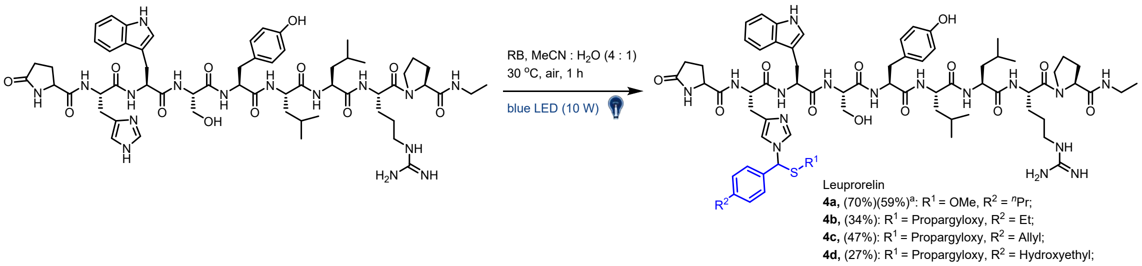

The reaction was followed General Procedure C using peptide **4** (100 uL 1 mM solution, sequence: NH-Pyr**H**WSYLLR-NHEt) and thioacetal **1a**, **1c**, **1d** and **1e**. A stock solution of peptide **4** (1mM) was made up by 2 mL solvent (MeCN/H_2O = 4/1) and 2.4 mg peptide **4**.

Before the reaction, a quantity relationship of matter between peptide **4** and the internal standard (dibenzyl sulfoxide) was established by a gradient concentration. The volume of the reaction mixture was adjusted to 500 uL with MeCN/H_2O (1/1), and then 2 uL dibenzyl sulfoxide stock solution (50 mM in MeCN/H_2O = 1/1) was added. The resulted solution was filtrated and analyzed with LC-MS.

A 10 mg level reaction between **1a** and peptide **4** was followed the similar procedure. 10.0 mg of peptide **4** was dissolved in the solvent (5 mL MeCN/H_2O = 4/1) in a 20 mL vial. 45 mg thioacetal **1a** and 1.1 mg RB was added to the mixture. The vial was then caped and equipped with magnetic bar. The reaction was set in a reaction chamber equipped with magnetic stirrer, 450 nm LED lamp (10 W) and exhaust fan to maintain the reaction temperature at about 30 °C for 1 hour. The resulting solution was then purified via a preparative LC after filtration. (10-80% B phase over 20 min, 8 mL·min^{-1} flow rate, 00.1% TFA, λ = 220 nm, Shimadzu Shim-pack GIST C18 20 × 250 mm, 5 µm column). Desired distillates of preparative LC were identified by MS and lyophilized to obtain target products, and analyzed by NMR. Liquid nitrogen was used to quickly freeze distillates and lyophilize them as quickly as possible using a lyophilizer. 6.8 mg (59%) white powder was obtained as target product.

Characterization data of **4a**

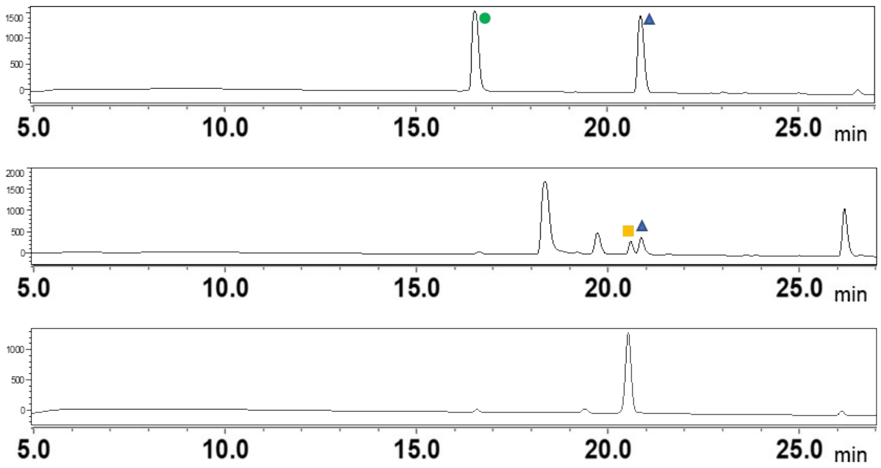

HPLC traces of the quantity relationship of matter, crud reaction and purified **4a**. (10-80% B phase over 25 min, 1 mL·min^{-1} flow rate, 0.1% TFA, λ = 220 nm, Kromasil 100-5-C18 4.6 × 250 mm, 5 µm column). The green circle, blue triangle and orange square refer to the starting peptide, internal standard and target product.

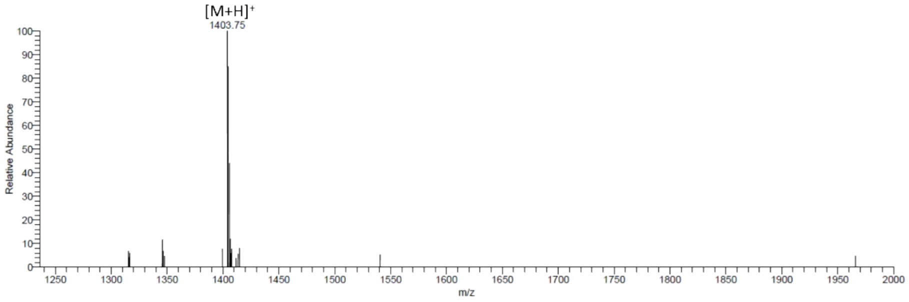

ESI Mass spectrum of purified product **4a**. Calculated Mass $[M+H]^+$: 1403.75; Mass Found (ESI+) $[M+H]^+$: 1403.75.

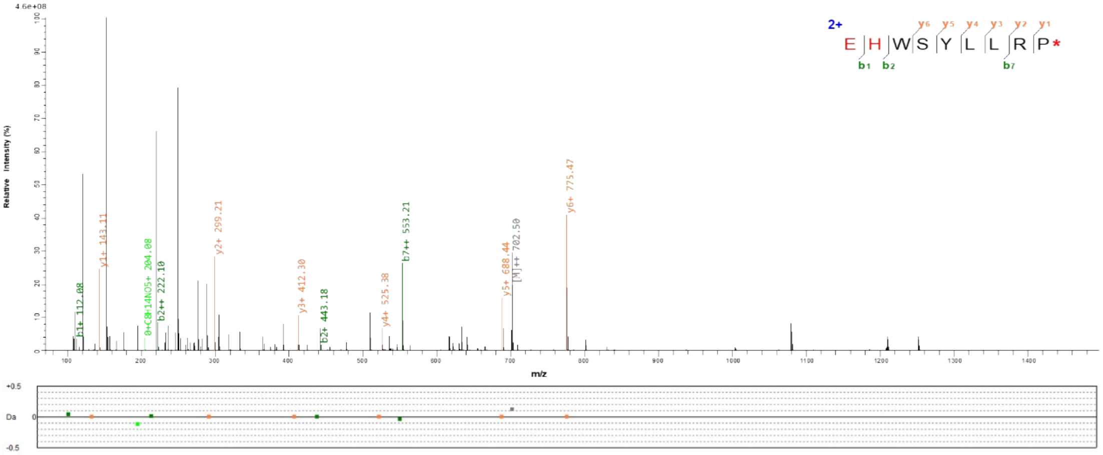

#	E(1/*)	H(2/8)	W(3/7)	S(4/6)	Y(5/5)	L(6/4)	L(7/3)	R(8/2)	P(*/1)
b+	112.0393	443.1748	629.2541	716.2861	879.3494	992.4335	1105.5175	1261.6186	*
b++	56.5233	222.0910	315.1307	358.6467	440.1783	496.7204	553.2624	631.3130	*
y+	*	1292.6972	961.5618	775.4825	688.4504	525.3871	412.3031	299.2190	143.1179
y++	*	646.8523	481.2845	388.2449	344.7289	263.1972	206.6552	150.1131	72.0626

MS/MS analysis of **4a**

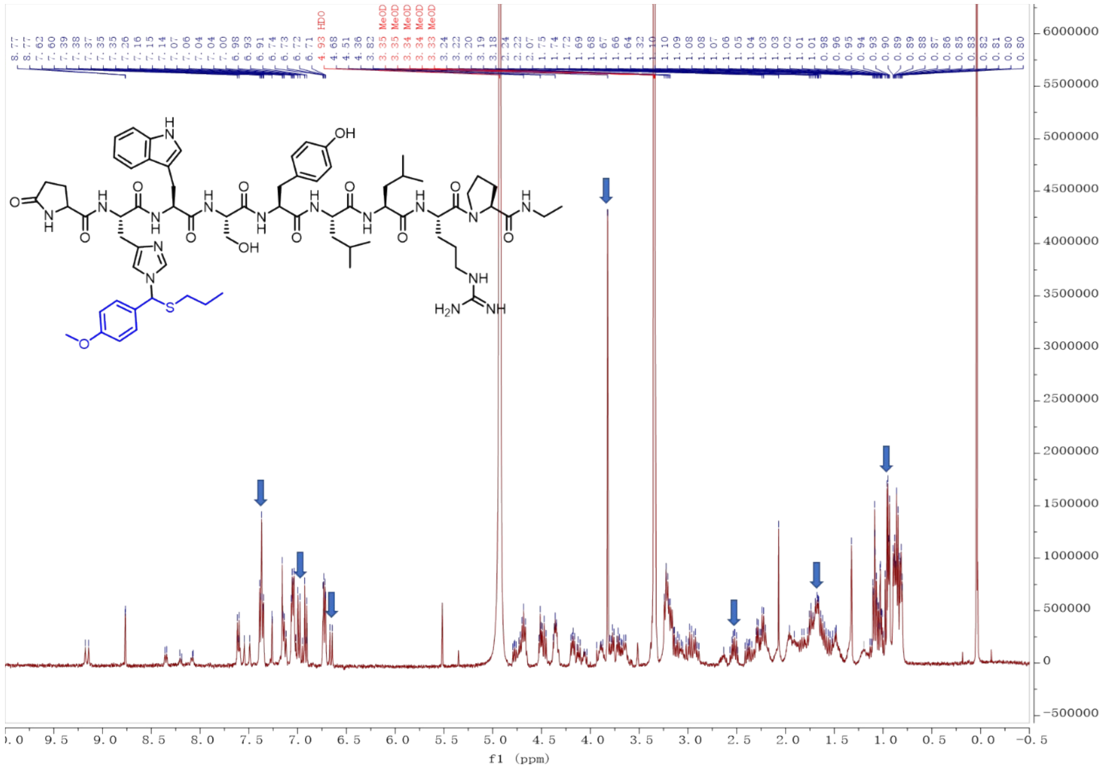

^{1}H NMR (400 MHz, MeOD) analysis of **4a**. Blue arrows point to signals of adducted thioacetal.

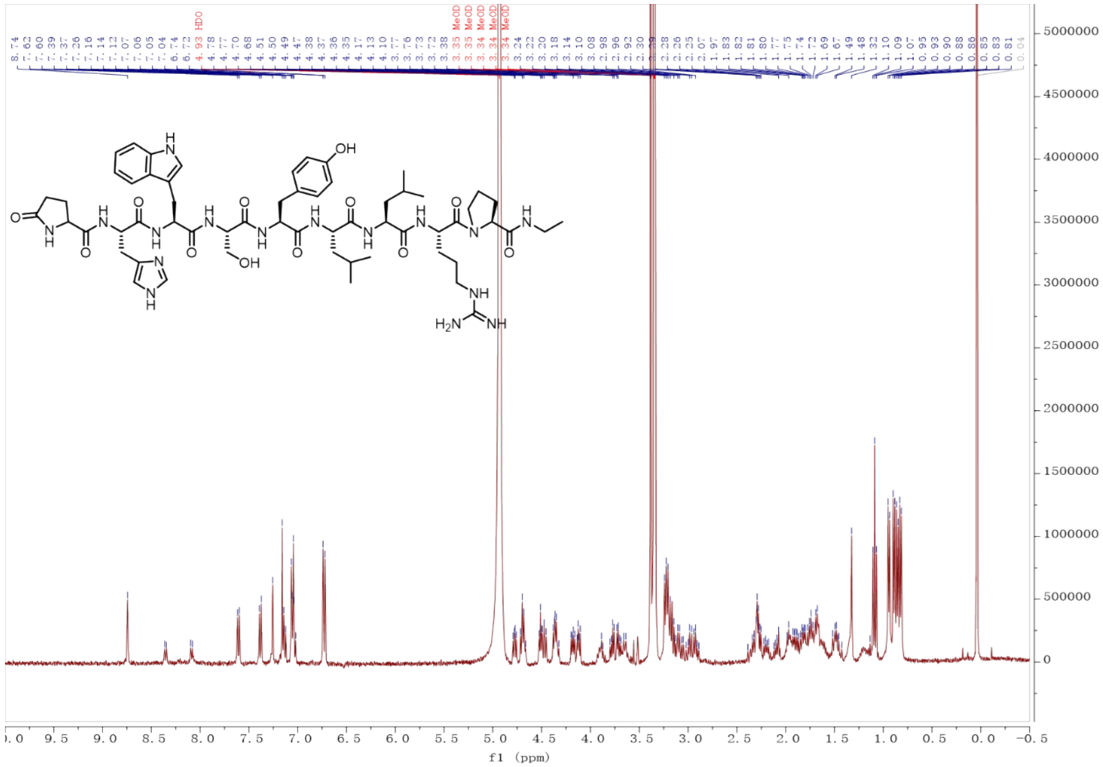

A control spectrum of ^{1}H NMR (400 MHz, MeOD) analysis of Leuprorelin

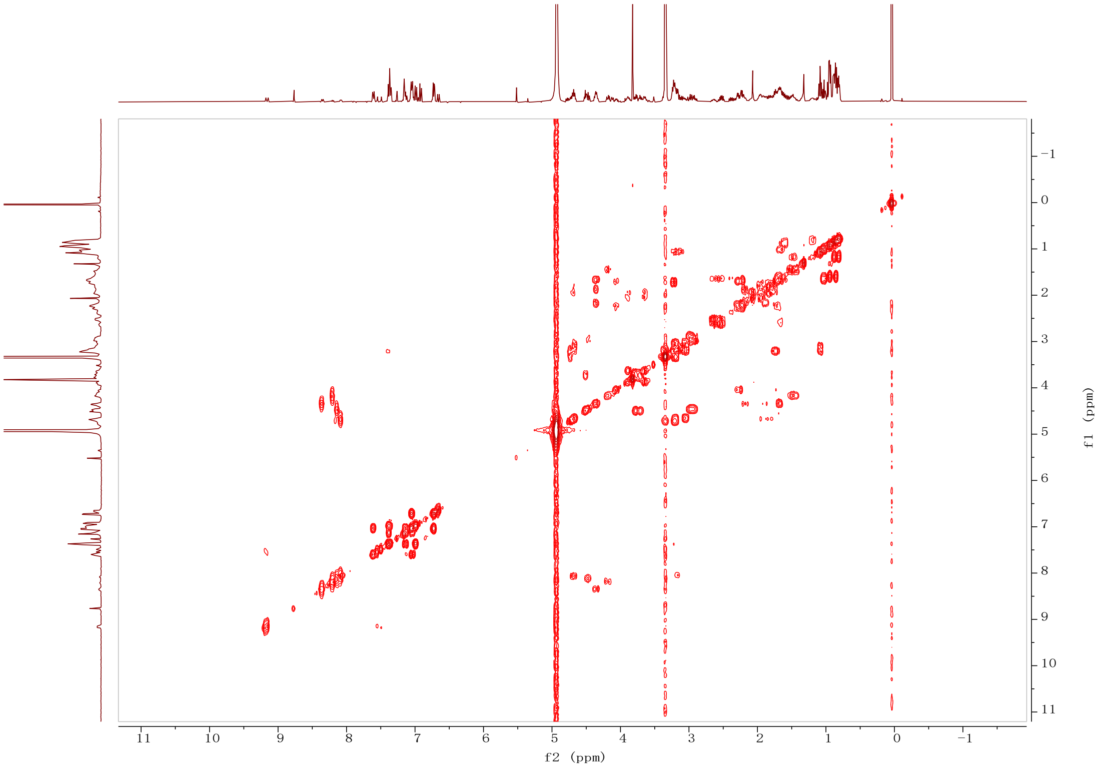

^1H-^1H COSY NMR spectrum of **4a**

Characterization data of **4b**

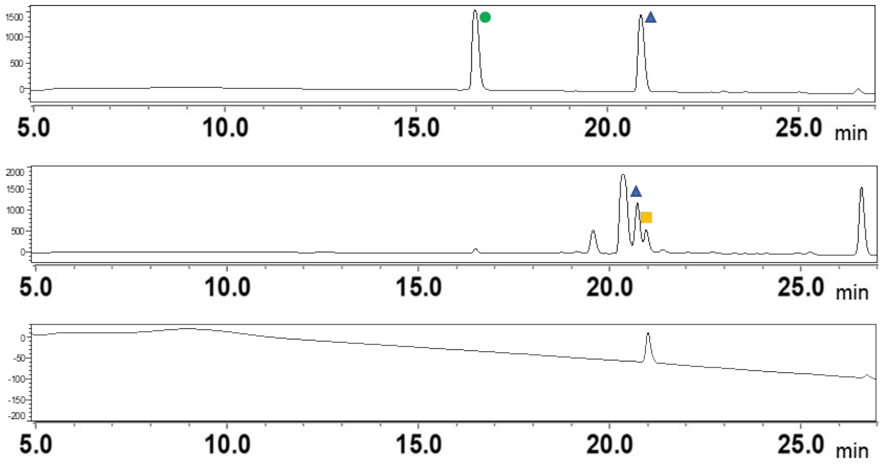

HPLC traces of the quantity relationship of matter, crud reaction and purified **4b**. (10-80% B phase over 25 min, 1 mL·min^{-1} flow rate, 0.1% TFA, λ = 220 nm, Kromasil 100-5-C18 4.6 × 250 mm, 5 µm column). The green circle, blue triangle and orange square refer to the starting peptide, internal standard and target product.

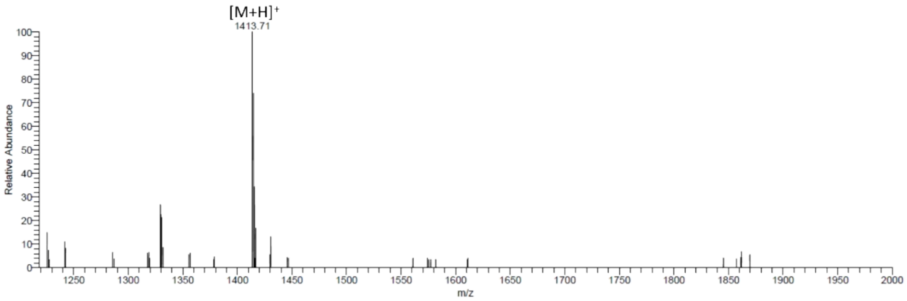

ESI Mass spectrum of purified product **4b**. Calculated Mass $[M+H]^+$: 1413.71; Mass Found (ESI+) $[M+H]^+$: 1413.71.

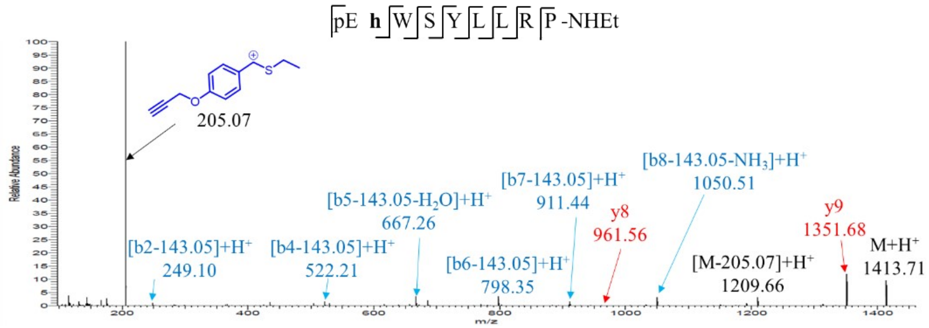

#	b	b-H_2O	b-NH_3	b(2+)	Seq	y	y-H_2O	y-NH_3	y(2+)	#
1	112.04	94.03	95.01	56.52	pE	1351.69	1333.68	1334.66	676.345	9
2	391.14	373.13	374.11	196.07	H(+143.05)	1240.66	1222.65	1223.63	620.83	8
3	577.22	559.21	560.19	289.11	W	961.56	943.55	944.53	481.28	7
4	664.25	646.24	647.22	332.625	S	775.48	757.47	758.45	388.24	6
5	827.31	809.3	810.28	414.155	Y	688.45	670.44	671.42	344.725	5
6	940.40	922.39	923.37	470.7	L	525.39	507.38	508.36	263.195	4
7	1053.48	1035.47	1036.45	527.24	L	412.30	394.29	395.27	206.65	3
8	1209.58	1191.57	1192.55	605.29	R	299.22	281.21	282.19	150.11	2
9	1306.64	1288.63	1289.61	653.82	P	143.12	125.11	126.09	72.06	1

MS/MS analysis of **4b**

Characterization data of **4c**

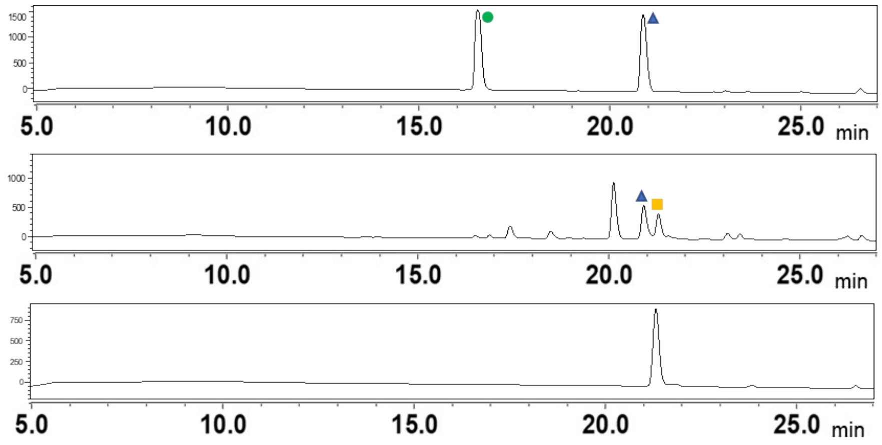

HPLC traces of the quantity relationship of matter, crud reaction and purified **4c**. (10-80% B phase over 25 min, 1 mL·min^{-1} flow rate, 0.1% TFA, λ = 220 nm, Kromasil 100-5-C18 4.6 × 250 mm, 5 µm column). The green circle, blue triangle and orange square refer to the starting peptide, internal standard and target product.

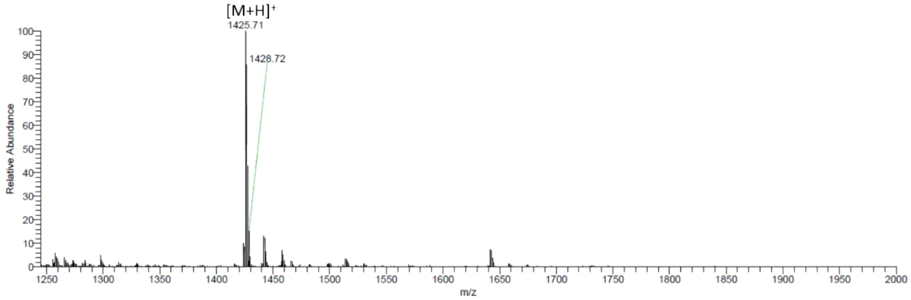

ESI Mass spectrum of purified product **4c**. Calculated Mass $[M+H]^+$: 1425.71; Mass Found (ESI+) $[M+H]^+$: 1425.71.

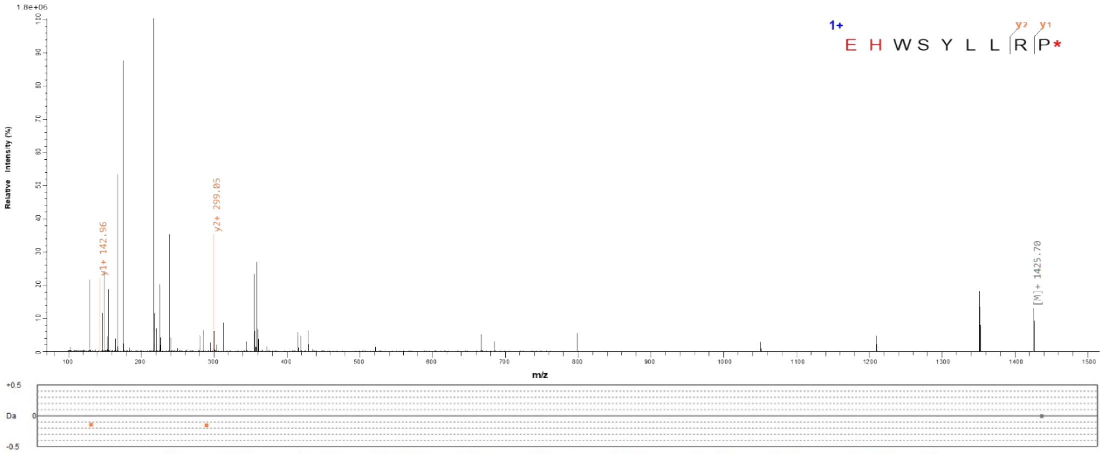

#	E(1/*)	H(2/8)	W(3/7)	S(4/6)	Y(5/5)	L(6/4)	L(7/3)	R(8/2)	P(*/1)
b+	112.0393	465.1591	651.2384	738.2705	901.3338	1014.4178	1127.5019	1283.6030	*
b++	56.5233	233.0832	326.1229	369.6389	451.1705	507.7126	564.2546	642.3051	*
y+	*	1314.6816	961.5618	775.4825	688.4504	525.3871	412.3031	299.2190	143.1179
y++	*	657.8444	481.2845	388.2449	344.7289	263.1972	206.6552	150.1131	72.0626

MS/MS analysis of **4c**

Characterization data of **4d**

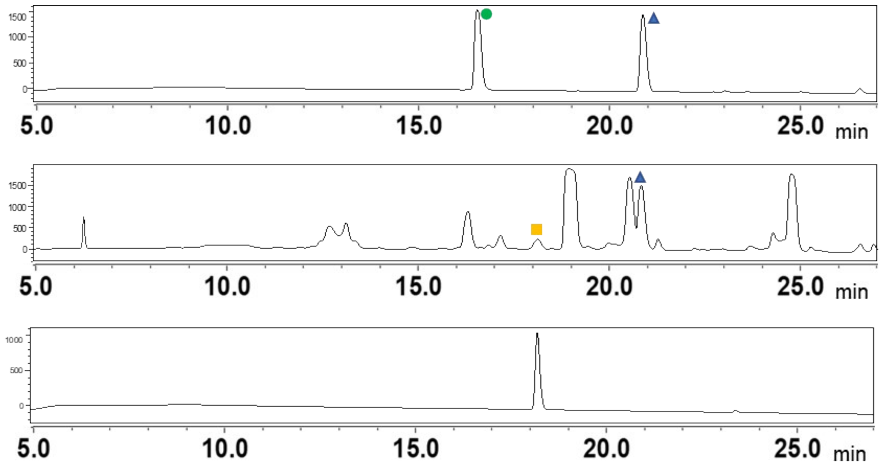

HPLC traces of the quantity relationship of matter, crud reaction and purified **4d**. (10-80% B phase over 25 min, 1 mL·min^{-1} flow rate, 0.1% TFA, λ = 220 nm, Kromasil 100-5-C18 4.6 × 250 mm, 5 µm column). The green circle, blue triangle and orange square refer to the starting peptide, internal standard and target product.

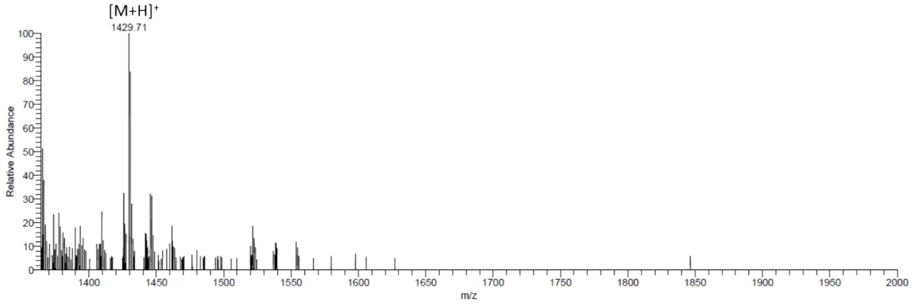

ESI Mass spectrum of purified product **4d**. Calculated Mass [M+H]$^{+}$: 1429.71; Mass Found (ESI+) [M+H]$^{+}$: 1429.71.

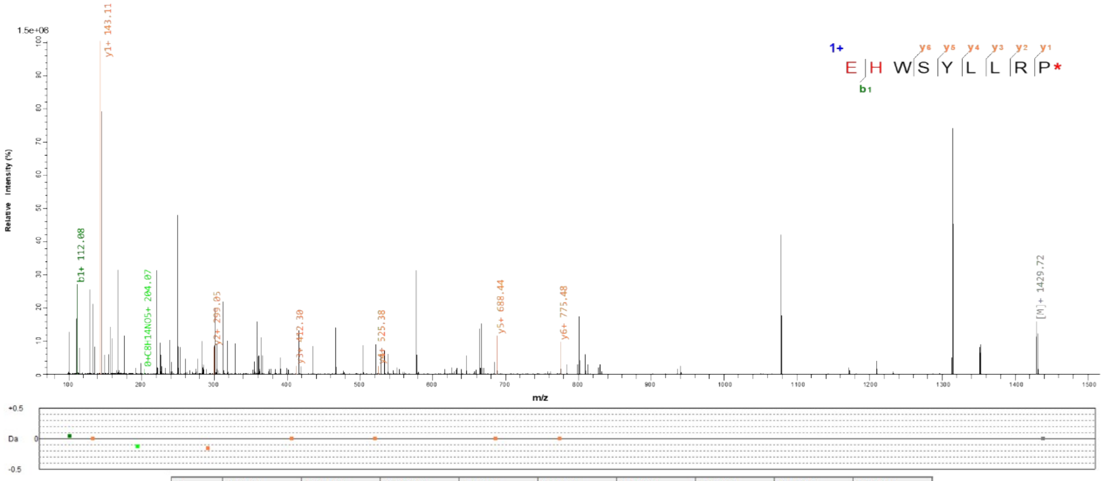

#	E(1/*)	H(2/8)	W(3/7)	S(4/6)	Y(5/5)	L(6/4)	L(7/3)	R(8/2)	P(*/1)
b+	112.0393	469.1540	655.2333	742.2654	905.3287	1018.4127	1131.4968	1287.5979	*
b++	56.5233	235.0806	328.1203	371.6363	453.1680	509.7100	566.2520	644.3026	*
y+	*	1318.6765	961.5618	775.4825	688.4504	525.3871	412.3031	299.2190	143.1179
y++	*	659.8419	481.2845	388.2449	344.7289	263.1972	206.6552	150.1131	72.0626

MS/MS analysis of **4d**

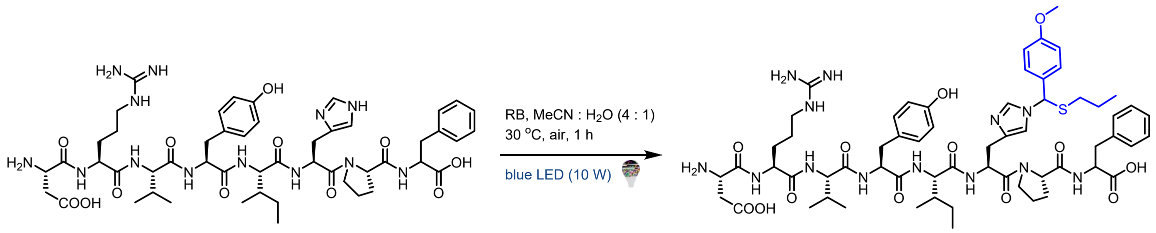

The reaction was followed General Procedure C using peptide **5** (100 uL 1 mM solution, sequence: NH_2-DRVYI**H**PF-OH) and thioacetal **1a**. A stock solution of peptide **5** (1mM) was made up by 2 mL solvent (MeCN/H_2O = 4/1) and 2.2 mg peptide **5**.

Before the reaction, a quantity relationship of matter between peptide **5** and the internal standard (dibenzyl sulfoxide) was established by a gradient concentration. The volume of the reaction mixture was adjusted to 500 uL with MeCN/H_2O (1/1), and then 2 uL dibenzyl sulfoxide stock solution (50 mM in MeCN/H_2O = 1/1) was added. The resulted solution was filtrated and analyzed with LC-MS.

Characterization data of **5**

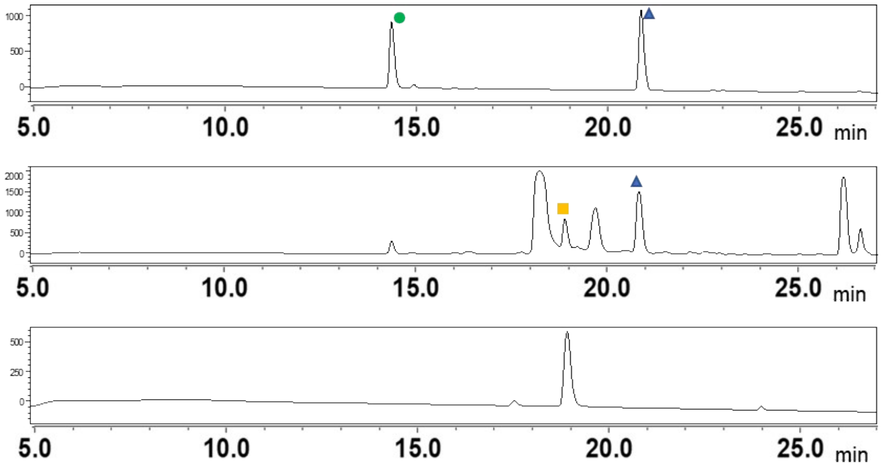

HPLC traces of the quantity relationship of matter, crud reaction and purified **5**. (10-80% B phase over 25 min, 1 mL·min^{-1} flow rate, 0.1% TFA, λ = 220 nm, Kromasil 100-5-C18 4.6 × 250 mm, 5 µm column). The green circle, blue triangle and orange square refer to the starting peptide, internal standard and target product.

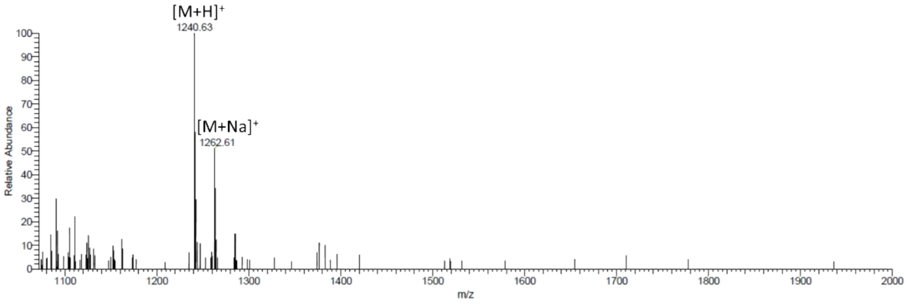

ESI Mass spectrum (positive charge) of purified product **5**. Calculated Mass $[M+H]^+$: 1240.63; Mass Found (ESI+) $[M+H]^+$: 1240.63, $[M+Na]^+$: 1262.61.

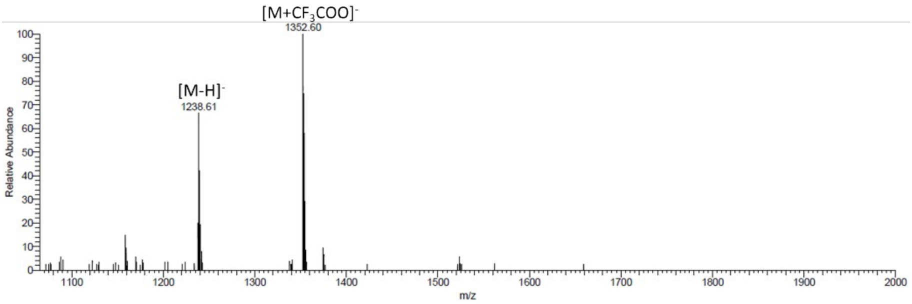

ESI Mass spectrum (negative charge) of purified product **5**. Calculated Mass $[M-H]^-$: 1238.61; Mass Found (ESI+) $[M-H]^-$: 1238.61, $[M+CF_3COO]^-$: 1352.60.

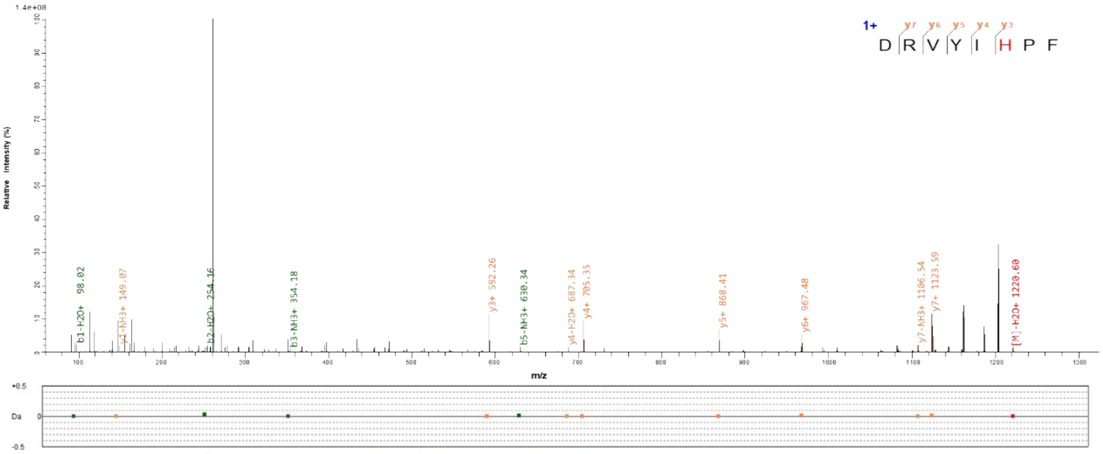

#	D(1/*)	R(2/7)	V(3/6)	Y(4/5)	I(5/4)	H(6/3)	P(7/2)	F(*/1)
b+	116.0342	272.1353	371.2037	534.2671	647.3511	976.4709	1073.5237	*
b++	58.5207	136.5713	186.1055	267.6372	324.1792	488.7391	537.2655	*
y+	*	1123.5757	967.4746	868.4062	705.3429	592.2588	263.1390	166.0862
y++	*	562.2915	484.2409	434.7067	353.1751	296.6330	132.0731	83.5468

MS/MS (negative charge) analysis of **5**

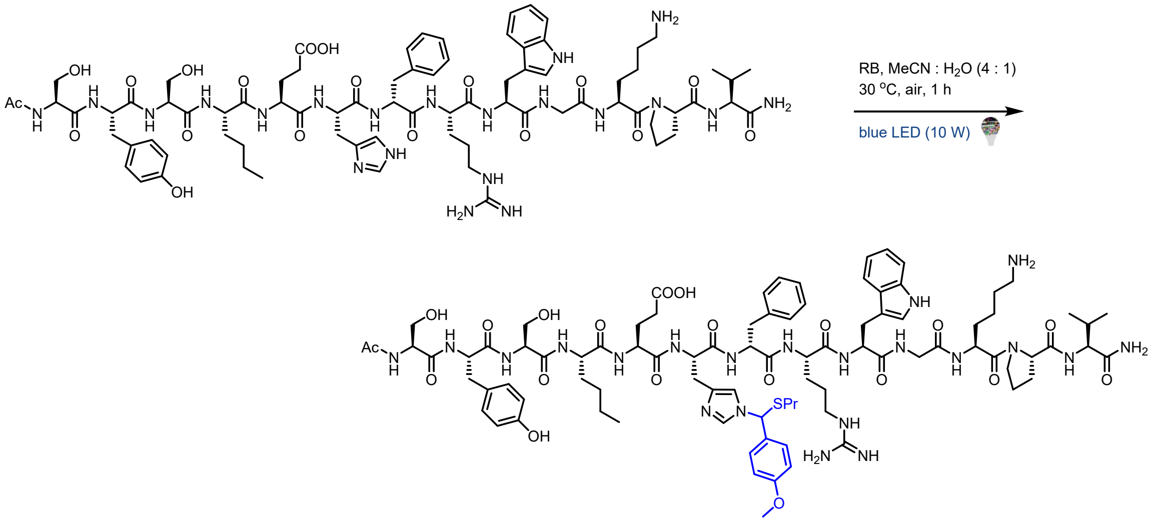

The reaction was followed General Procedure C using peptide **6** (100 uL 1 mM solution, sequence: Ac-SYSNleE**H**fRWGKPV-NH_2) and thioacetal **1a**. A stock solution of peptide **6** (1mM) was made up by 1 mL solvent (MeCN/H_2O = 4/1) and 1.7 mg peptide **6**.

Before the reaction, a quantity relationship of matter between peptide **6** and the internal standard (dibenzyl sulfoxide) was established by a gradient concentration. The volume of the reaction mixture was adjusted to 500 uL with MeCN/H_2O (1/1), and then 2 uL dibenzyl sulfoxide stock solution (50 mM in MeCN/H_2O = 1/1) was added. The resulted solution was filtrated and analyzed with LC-MS.

Characterization data of **6**

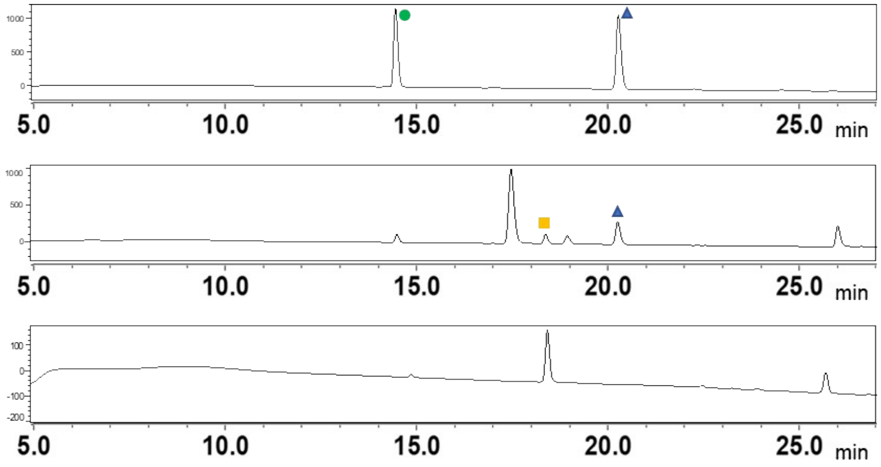

HPLC traces of the quantity relationship of matter, crud reaction and purified **6**. (10-70% B phase over 25 min, 1 mL·min^{-1} flow rate, 0.1% TFA, λ = 220 nm, Kromasil 100-5-C18 4.6 × 250 mm, 5 µm column). The green circle, blue triangle and orange square refer to the starting peptide, internal standard and target product.

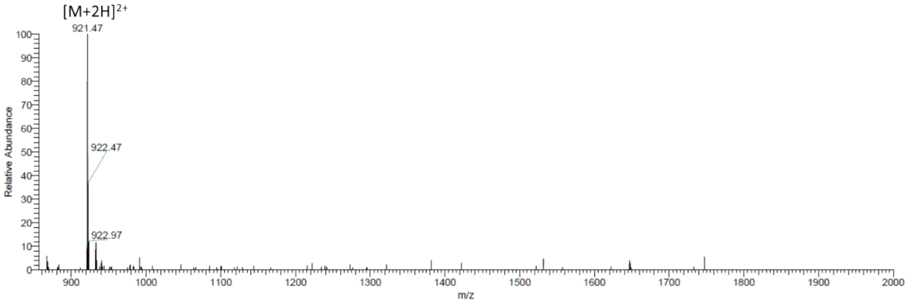

ESI Mass spectrum of purified product **6**. Calculated Mass $[M+2H]^{2+}$: 921.47; Mass Found (ESI+) $[M+2H]^{2+}$: 921.47.

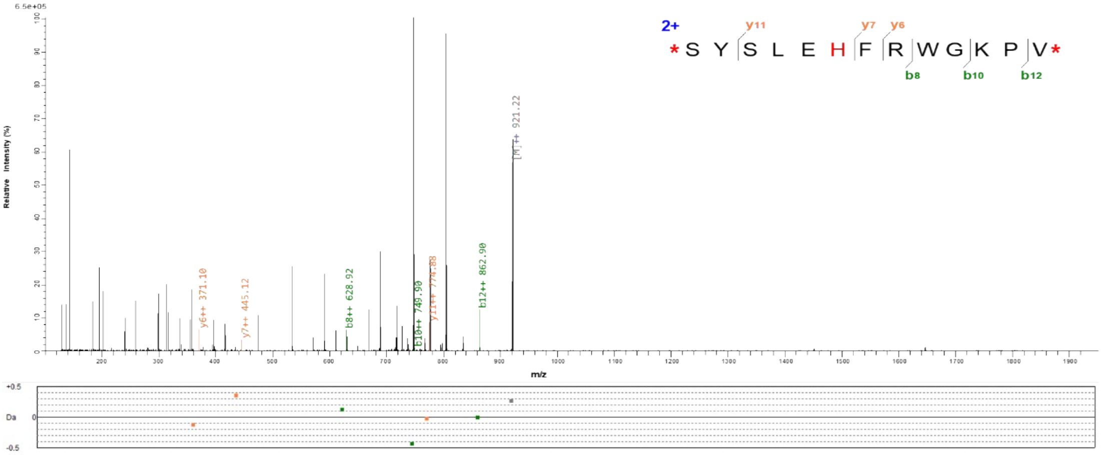

#	S(1/*)	Y(2/12)	S(3/11)	L(4/10)	E(5/9)	H(6/8)	F(7/7)	R(8/6)	W(9/5)	G(10/4)	K(11/3)	P(12/2)	V(*/1)
b+	130.0499	293.1132	380.1452	493.2293	622.2719	953.4073	1100.4757	1256.5768	1442.6561	1499.6776	1627.7726	1724.8253	*
b++	65.5286	147.0602	190.5762	247.1183	311.6396	477.2073	550.7415	628.7921	721.8317	750.3424	814.3899	862.9163	*
y+	*	1711.8777	1548.8144	1461.7823	1348.6983	1219.6557	888.5202	741.4518	585.3507	399.2714	342.2500	214.1550	117.1022
y++	*	856.4425	774.9108	731.3948	674.8528	610.3315	444.7638	371.2296	293.1790	200.1393	171.6286	107.5811	59.0548

MS/MS analysis of **6**

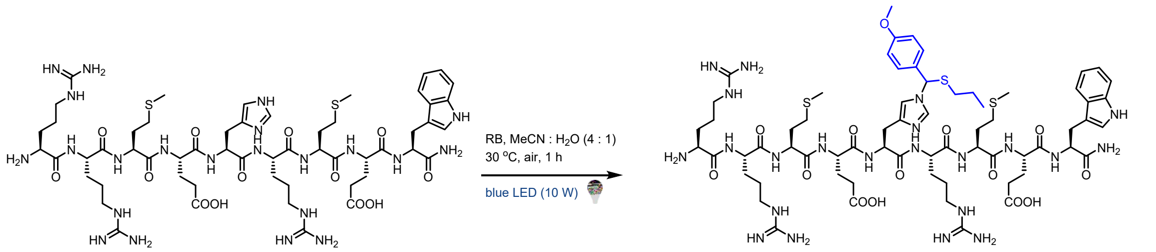

The reaction was followed General Procedure C using peptide **7** (100 uL 1 mM solution, sequence: NH_2-RRME**H**RMEW-NH_2) and thioacetal **1a**. A stock solution of peptide **7** (1 mM) was made up by 1 mL solvent (MeCN/H_2O = 4/1) and 1.3 mg peptide **7**.

Before the reaction, a quantity relationship of matter between peptide **7** and the internal standard (dibenzyl sulfoxide) was established by a gradient concentration. The volume of the reaction mixture was adjusted to 500 uL with MeCN/H_2O (1/1), and then 2 uL dibenzyl sulfoxide stock solution (50 mM in MeCN/H_2O = 1/1) was added. The resulted solution was filtrated and analyzed with LC-MS.

Characterization data of **7**

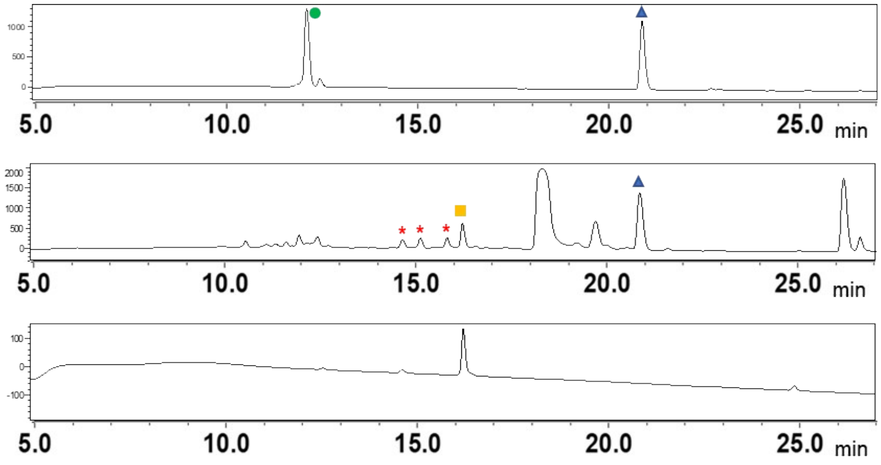

HPLC traces of the quantity relationship of matter, crud reaction and purified **7**. (10-80% B phase over 25 min, 1 mL·min^{-1} flow rate, 0.1% TFA, λ = 220 nm, Kromasil 100-5-C18 4.6 × 250 mm, 5 µm column). The green circle, blue triangle and orange square refer to the starting peptide, internal standard and target product. The red asterisks indicate oxidative products with both oxidized Met and modified His.

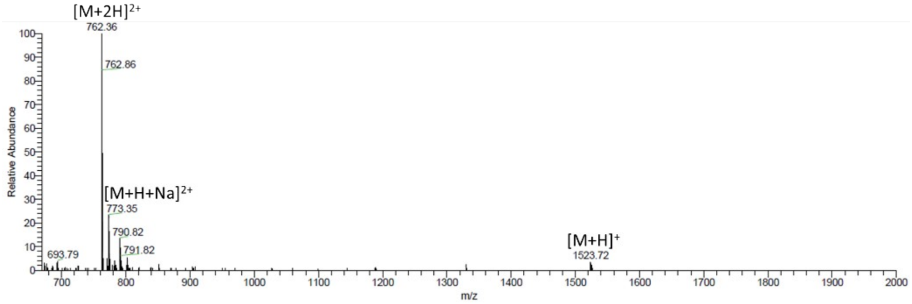

ESI Mass spectrum of purified product **7**. Calculated Mass $[M+H]^+$: 1523.72, $[M+2H]^{2+}$: 762.36; Mass Found (ESI+) $[M+H]^+$: 1523.72, $[M+H+Na]^{2+}$: 773.35, $[M+2H]^{2+}$: 762.36.

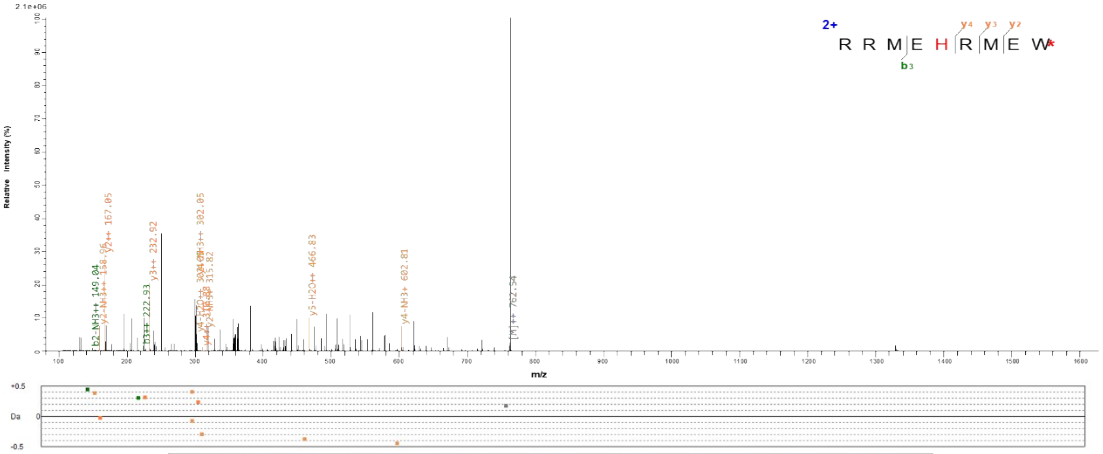

#	R(1/*)	R(2/8)	M(3/7)	E(4/6)	H(5/5)	R(6/4)	M(7/3)	E(8/2)	W(*/1)
b+	157.1084	313.2095	444.2500	573.2926	904.4280	1060.5291	1191.5696	1320.6122	*
b++	79.0578	157.1084	222.6286	287.1499	452.7177	530.7682	596.2885	660.8097	*
y+	*	1367.6170	1211.5159	1080.4754	951.4328	620.2973	464.1962	333.1557	204.1131
y++	*	684.3121	606.2616	540.7413	476.2200	310.6523	232.6018	167.0815	102.5602

MS/MS analysis of **7**

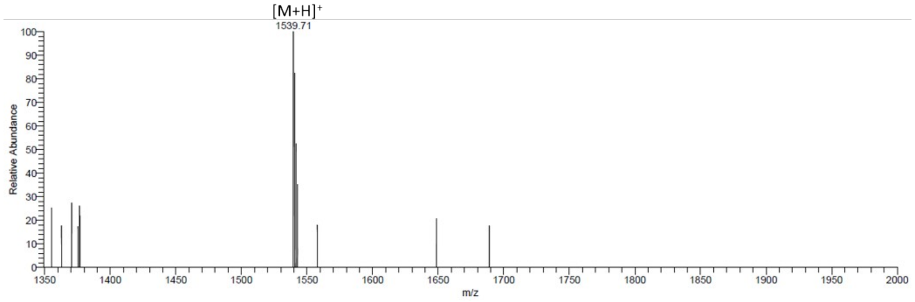

ESI Mass spectrum of purified product **7** with both mono-oxidized Met and modified His. Calculated Mass $[M+H]^+$: 1539.71; Mass Found (ESI+) $[M+H]^+$: 1539.71.

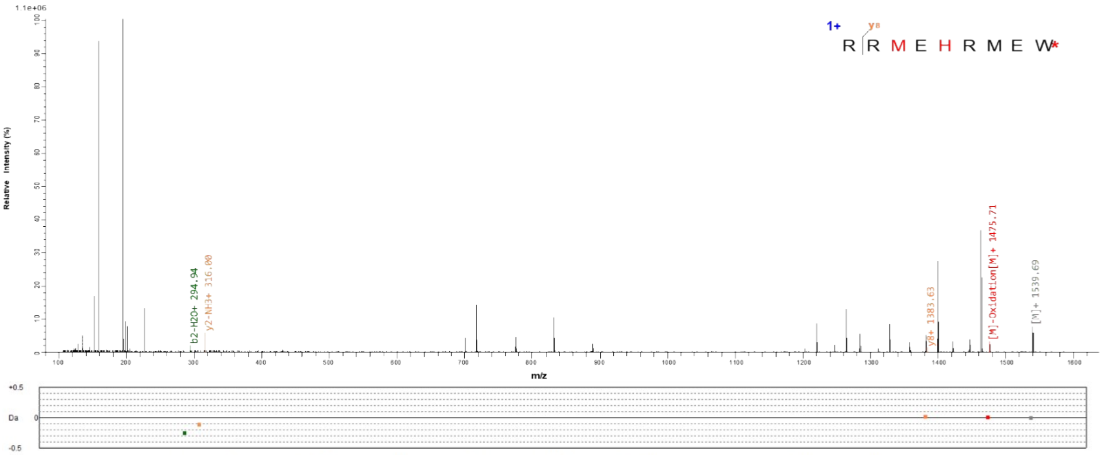

#	R(1/*)	R(2/8)	M(3/7)	E(4/6)	H(5/5)	R(6/4)	M(7/3)	E(8/2)	W(*/1)
b+	157.1084	313.2095	460.2449	589.2875	920.4229	1076.5240	1207.5645	1336.6071	*
b++	79.0578	157.1084	230.6261	295.1474	460.7151	538.7657	604.2859	668.8072	*
y+	*	1383.6119	1227.5108	1080.4754	951.4328	620.2973	464.1962	333.1557	204.1131
y++	*	692.3096	614.2590	540.7413	476.2200	310.6523	232.6018	167.0815	102.5602

MS/MS analysis of **7** with both mono-oxidized Met and modified His

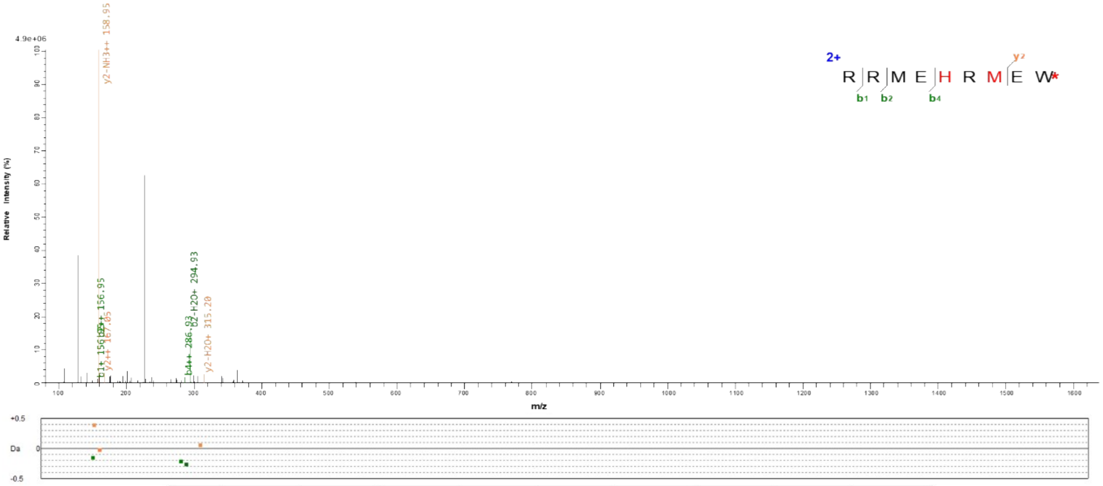

#	R(1/*)	R(2/8)	M(3/7)	E(4/6)	H(5/5)	R(6/4)	M(7/3)	E(8/2)	W(*/1)
b+	157.1084	313.2095	444.2500	573.2926	904.4280	1060.5291	1207.5645	1336.6071	*
b++	79.0578	157.1084	222.6286	287.1499	452.7177	530.7682	604.2859	668.8072	*
y+	*	1383.6119	1227.5108	1096.4703	967.4277	636.2922	480.1911	333.1557	204.1131
y++	*	692.3096	614.2590	548.7388	484.2175	318.6498	240.5992	167.0815	102.5602

MS/MS analysis of **7** with both mono-oxidized Met and modified His

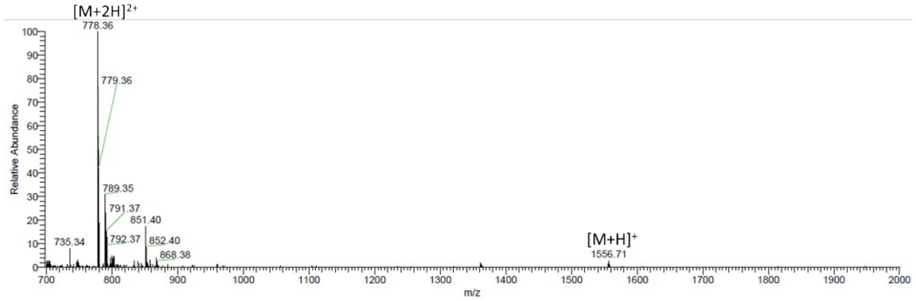

ESI Mass spectrum of purified product **7** with both di-oxidized Met and modified His. Calculated Mass [M+H]$^+$: 1556.71, [M+2H]$^{2+}$: 778.36; Mass Found (ESI+) [M+H]$^+$: 1556.71, [M+2H]$^{2+}$: 778.36.

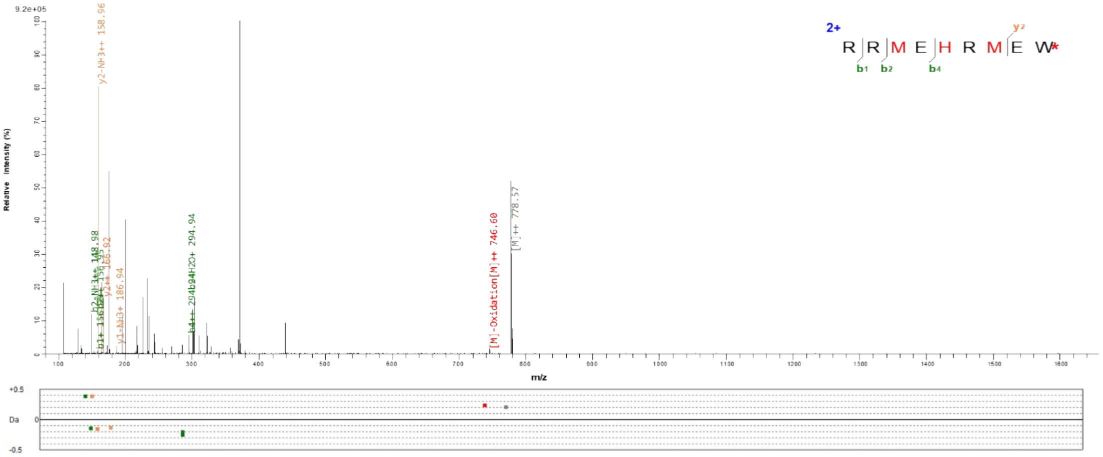

#	R(1/*)	R(2/8)	M(3/7)	E(4/6)	H(5/5)	R(6/4)	M(7/3)	E(8/2)	W(*/1)
b+	157.1084	313.2095	460.2449	589.2875	920.4229	1076.5240	1223.5595	1352.6020	*
b++	79.0578	157.1084	230.6261	295.1474	460.7151	538.7657	612.2834	676.8047	*
y+	*	1399.6068	1243.5057	1096.4703	967.4277	636.2922	480.1911	333.1557	204.1131
y++	*	700.3070	622.2565	548.7388	484.2175	318.6498	240.5992	167.0815	102.5602

MS/MS analysis of **7** with both di-oxidized Met and modified His

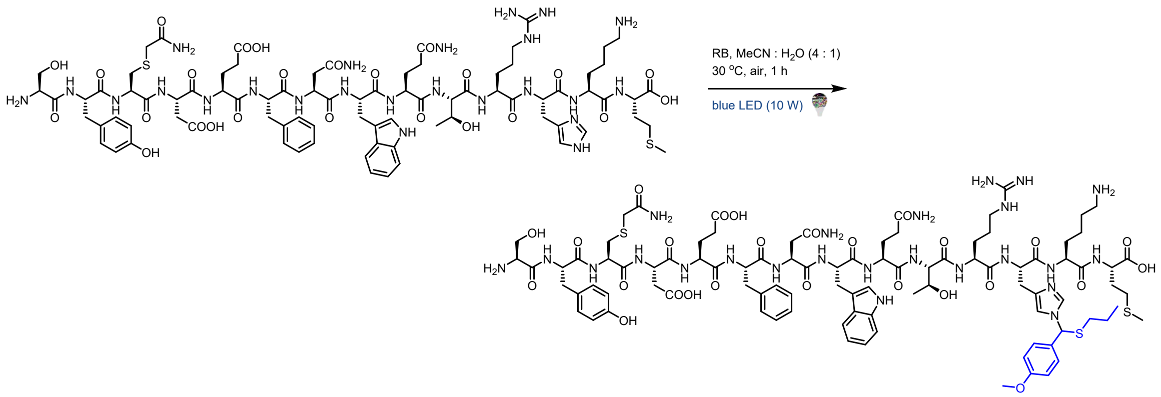

The reaction was followed General Procedure C using peptide **8** (100 uL 1 mM solution, sequence: NH_2- SYCDEFNWQTR**H**KM-NH_2) and thioacetal **1a**. A stock solution of peptide **7** (1 mM) was made up by 1 mL solvent (MeCN/H_2O = 4/1) and 1.9 mg peptide **8**.

Before the reaction, a quantity relationship of matter between peptide **7** and the internal standard (dibenzyl sulfoxide) was established by a gradient concentration. The volume of the reaction mixture was adjusted to 500 uL with MeCN/H_2O (1/1), and then 2 uL dibenzyl sulfoxide stock solution (50 mM in MeCN/H_2O = 1/1) was added. The resulted solution was filtrated and analyzed with LC-MS.

Characterization data of **8**

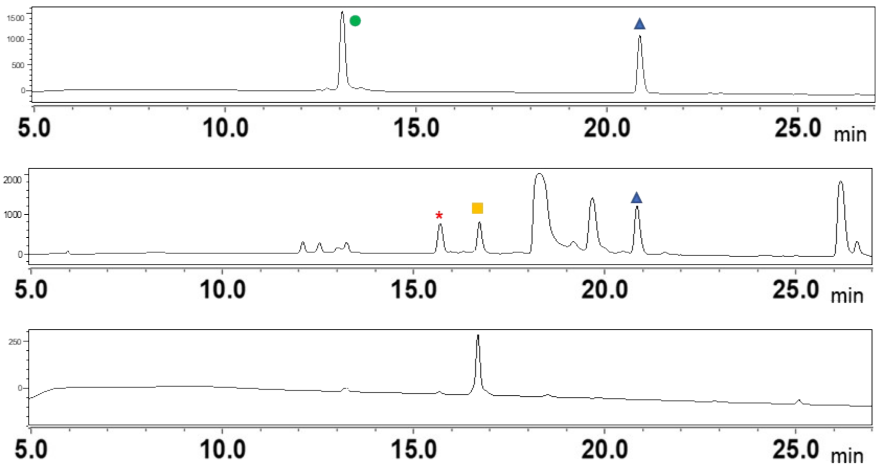

HPLC traces of the quantity relationship of matter, crud reaction and purified **8**. (10-80% B phase over 25 min, 1 mL·min^{-1} flow rate, 0.1% TFA, λ = 220 nm, Kromasil 100-5-C18 4.6 × 250 mm, 5 μm column). The green circle, blue triangle and orange square refer to the starting peptide, internal standard and target product. The red asterisks indicate oxidative products with both oxidized Met and modified His.

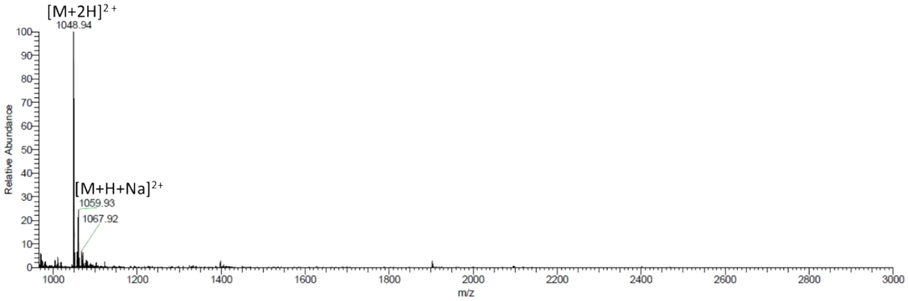

ESI Mass spectrum of purified product **8**. Calculated Mass [M+2H]$^{2+}$: 1048.94; Mass Found (ESI+) [M+2H]$^{2+}$: 1048.94, [M+H+Na]$^{2+}$: 1059.93.

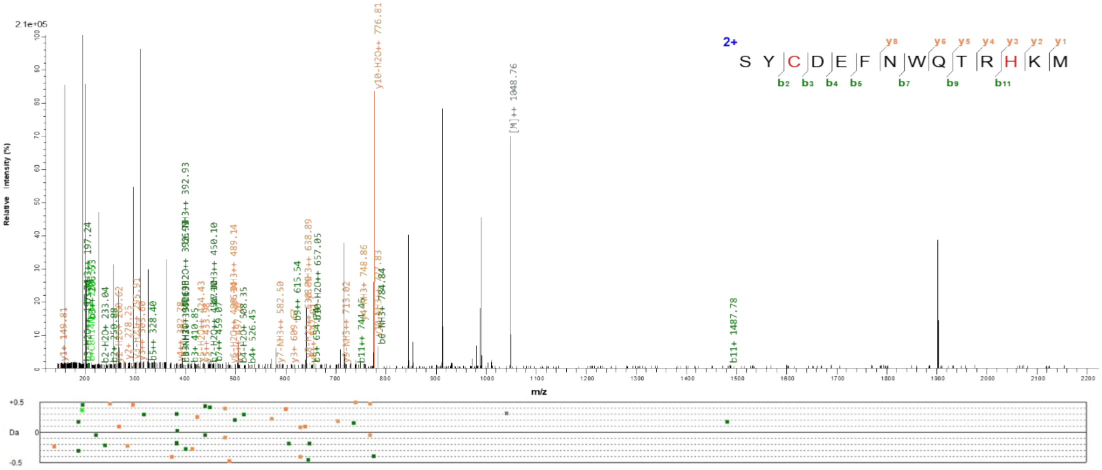

#	S(1/*)	Y(2/13)	C(3/12)	D(4/11)	E(5/10)	F(6/9)	N(7/8)	W(8/7)	Q(9/6)	T(10/5)	R(11/4)	H(12/3)	K(13/2)	M(*/1)
b+	88.0393	251.1026	411.1333	526.1603	655.2028	802.2713	916.3142	1102.3935	1230.4521	1331.4998	1487.6009	1818.7363	1946.8313	*
b++	44.5233	126.0550	206.0703	263.5838	328.1051	401.6393	458.6607	551.7004	615.7297	666.2535	744.3041	909.8718	973.9193	*
y+	*	2008.8503	1845.7870	1685.7563	1570.7294	1441.6868	1294.6184	1180.5754	994.4961	866.4375	765.3899	609.2887	278.1533	150.0583
y++	*	1004.9288	923.3971	843.3818	785.8683	721.3470	647.8128	590.7914	497.7517	433.7224	383.1986	305.1480	139.5803	75.5328

MS/MS analysis of **8**

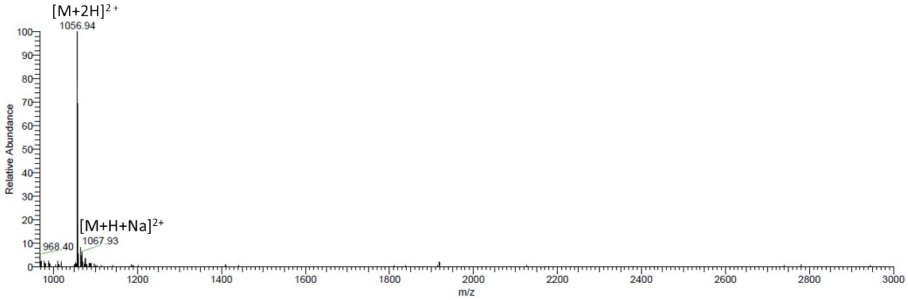

ESI Mass spectrum of purified product **8** with both mono-oxidized Met and modified His. Calculated Mass [M+2H]$^{2+}$: 1056.94; Mass Found (ESI+) [M+2H]$^{2+}$: 1056.94, [M+H+Na]$^{2+}$: 1067.93.

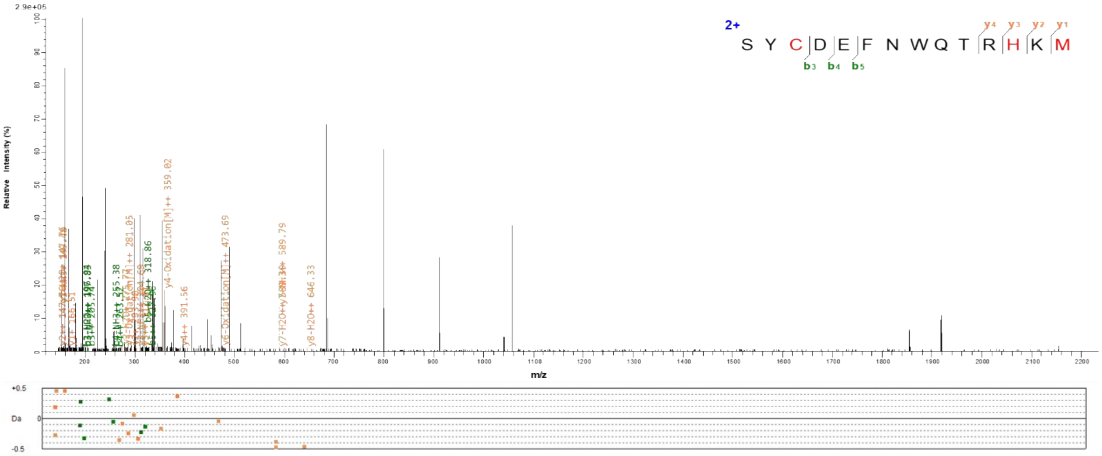

#	S(1/*)	Y(2/13)	C(3/12)	D(4/11)	E(5/10)	F(6/9)	N(7/8)	W(8/7)	Q(9/6)	T(10/5)	R(11/4)	H(12/3)	K(13/2)	M(*/1)
b+	88.0393	251.1026	411.1333	526.1603	655.2028	802.2713	916.3142	1102.3935	1230.4521	1331.4998	1487.6009	1818.7363	1946.8313	*
b++	44.5233	126.0550	206.0703	263.5838	328.1051	401.6393	458.6607	551.7004	615.7297	666.2535	744.3041	909.8718	973.9193	*
y+	*	2024.8452	1861.7819	1701.7512	1586.7243	1457.6817	1310.6133	1196.5703	1010.4910	882.4325	781.3848	625.2837	294.1482	166.0533
y++	*	1012.9263	931.3946	851.3792	793.8658	729.3445	655.8103	598.7888	505.7492	441.7199	391.1960	313.1455	147.5777	83.5303

MS/MS analysis of **8** with both mono-oxidized Met and modified His

5. References

[1] S. Learte-Aymami, C. Vidal, A. Gutierrez-Gonzalez, J. L. Mascarenas, *Angew. Chem. Int. Ed. Engl.* **2020**, *59*, 9149-9154.

[2] J. Ohata, M. B. Minus, M. E. Abernathy, Z. T. Ball, *J Am Chem Soc* **2016**, *138*, 7472-7475.

[3] Y. Takaoka, H. Tsutsumi, N. Kasagi, E. Nakata, I. Hamachi, *J. Am. Chem. Soc.* **2006**, *128*, 3273-3280.

[4] S. Jia, D. He, C. J. Chang, *J. Am. Chem. Soc.* **2019**, *141*, 7294-7301.

[5] X. Chen, F. Ye, X. Luo, X. Liu, J. Zhao, S. Wang, Q. Zhou, G. Chen, P. Wang, *J. Am. Chem. Soc.* **2019**.

[6] K. Peciak, E. Laurine, R. Tommasi, J. W. Choi, S. Brocchini, *Chem. Sci.* **2019**, *10*, 427-439.

[7] D. Kaiser, I. Klose, R. Oost, J. Neuhaus, N. Maulide, *Chem. Rev.* **2019**, *119*, 8701-8780.

[8] a) R. Parnes, H. Reiss, D. Pappo, *J. Org. Chem.* **2018**, *83*, 723-732; b) R. Parnes, D. Pappo, *Org. Lett.* **2015**, *17*, 2924-2927; c) R. Parnes, S. Narute, D. Pappo, *Org. Lett.* **2014**, *16*, 5922-5925.

[9] C. S. McKay, M. G. Finn, *Angew Chem Int Ed Engl* **2016**, *55*, 12643-12649.

[10] K. Susumu, H. T. Uyeda, I. L. Medintz, T. Pons, J. B. Delehanty, H. Mattoussi, *J Am Chem Soc* **2007**, *129*, 13987-13996.

[11] N. George, M. Bekkaye, G. Masson, J. Zhu, *European Journal of Organic Chemistry* **2011**, *2011*, 3695-3699.

6. NMR spectra

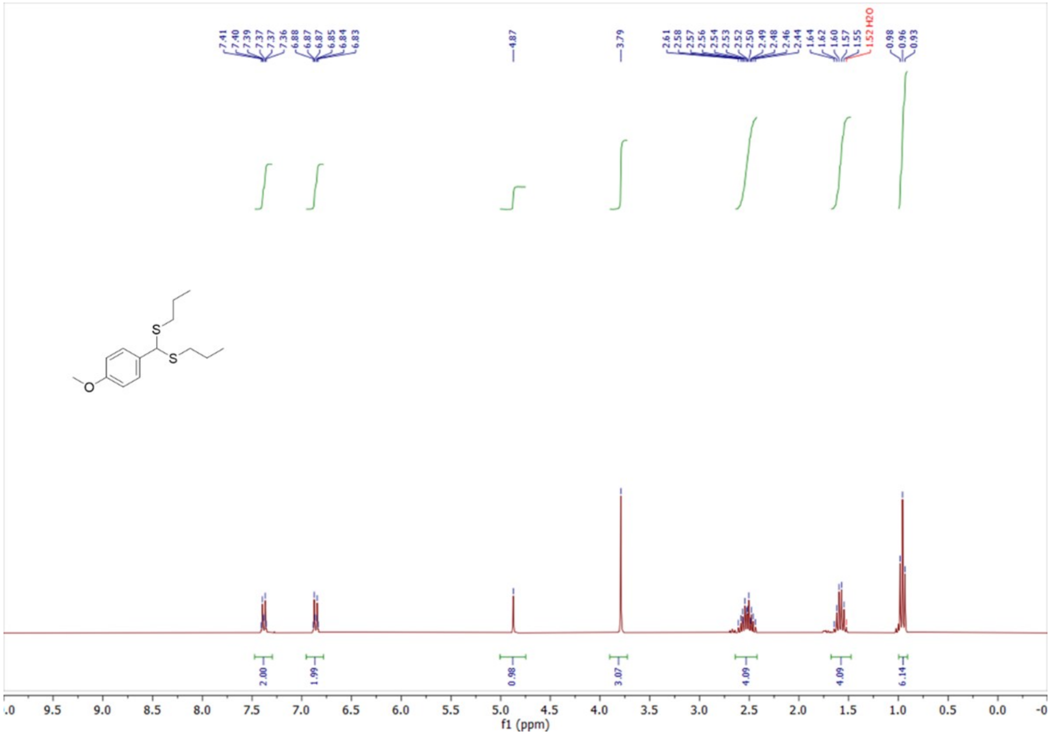

^1H NMR spectrum of **1a**

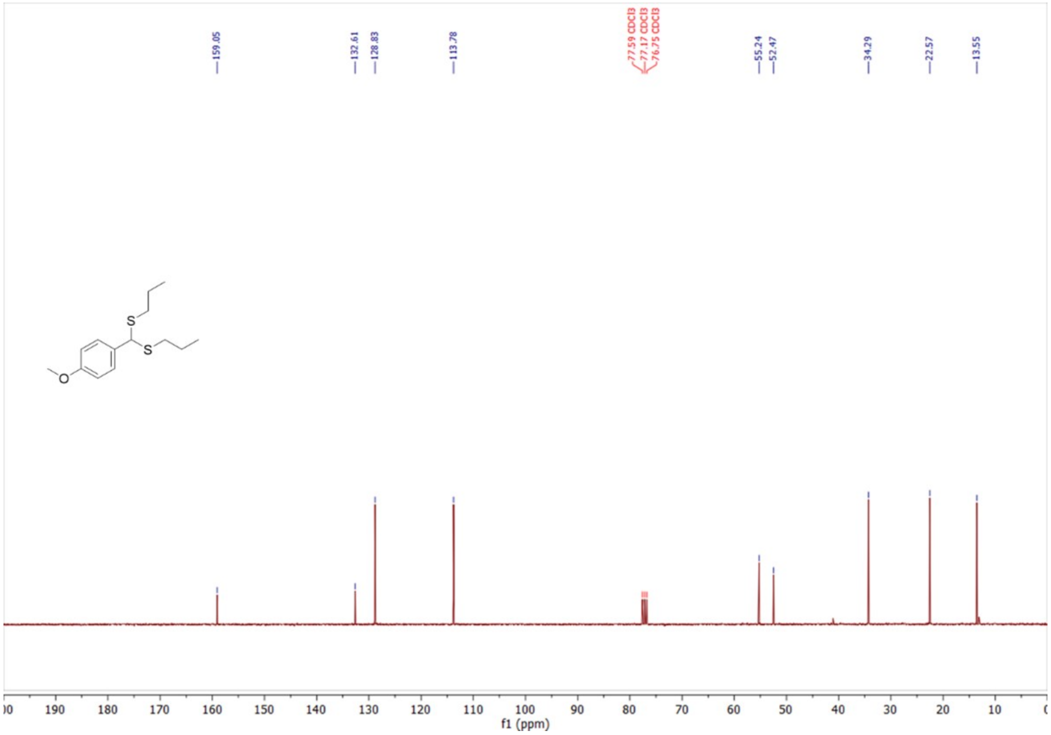

^{13}C NMR spectrum of **1a**

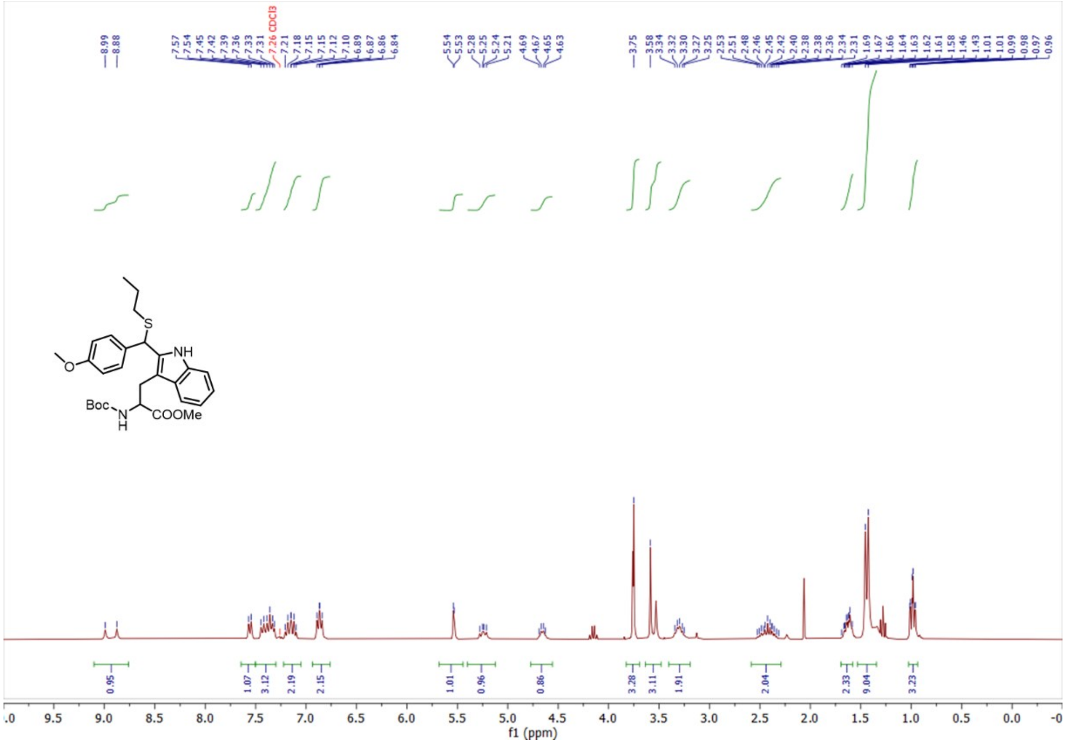

^1H NMR spectrum of Boc-Trp-OMe-**1a**

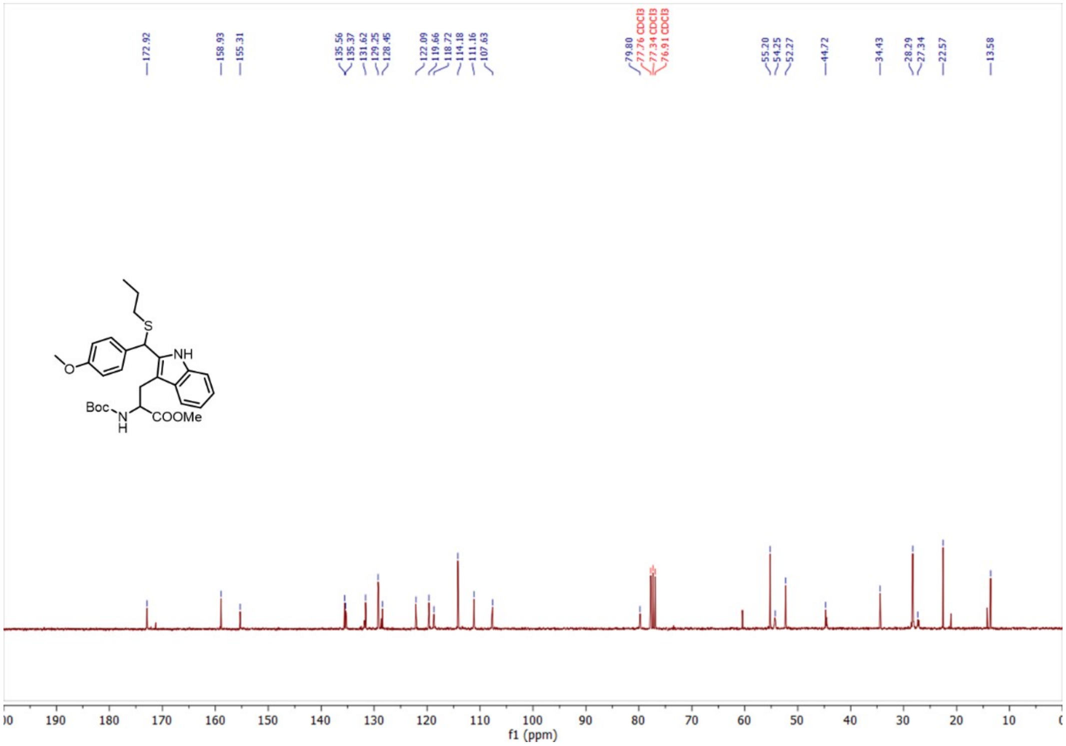

^{13}C NMR spectrum of Boc-Trp-OMe-**1a**

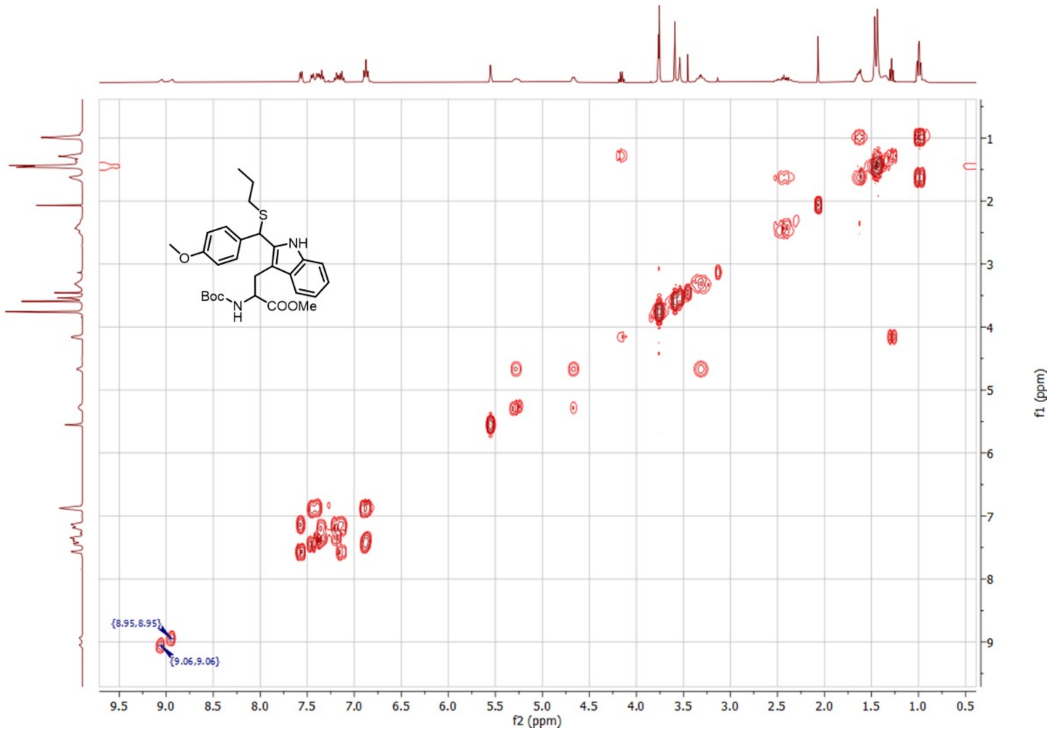

^1H-^1H COSY NMR spectrum of Boc-Trp-OMe-**1a**

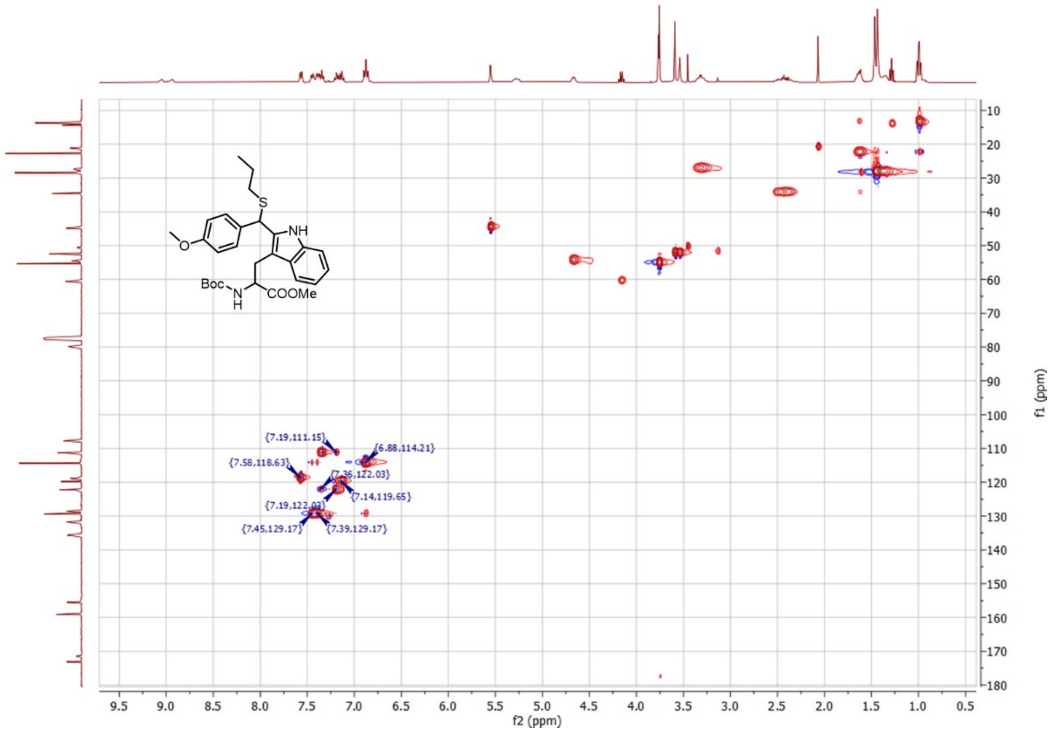

^1H-^{13}C HSQC NMR spectrum of Boc-Trp-OMe-**1a**

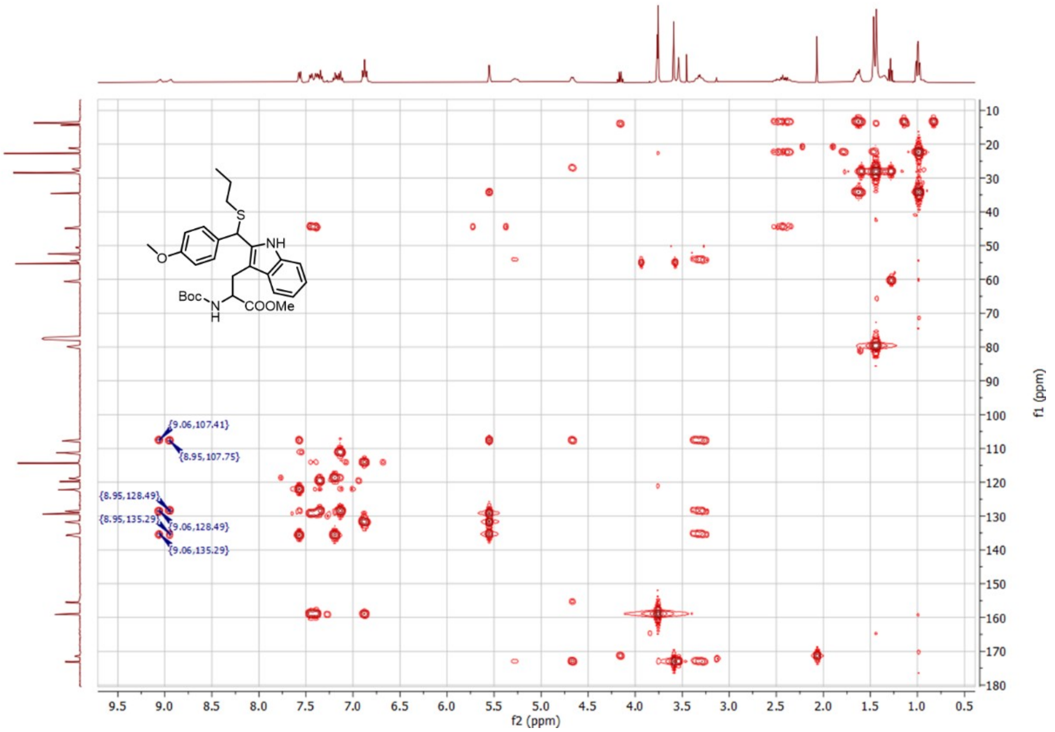

^{1}H-^{13}C HMBC NMR spectrum of Boc-Trp-OMe-**1a**

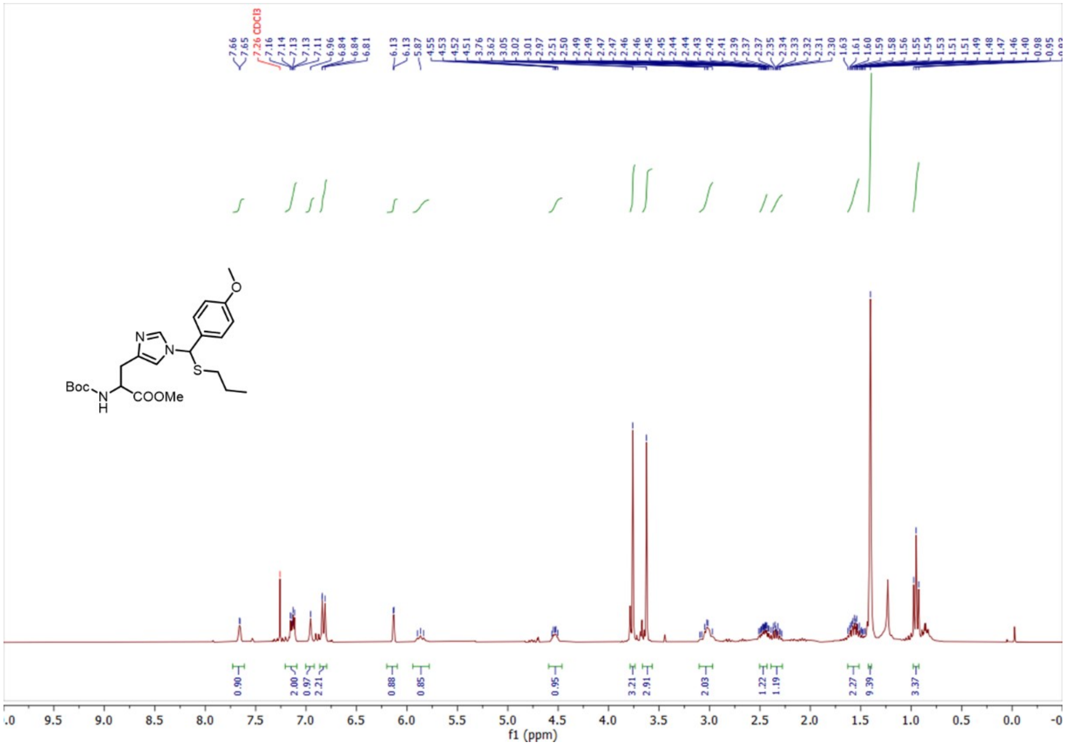

^{1}H NMR spectrum of **3a**

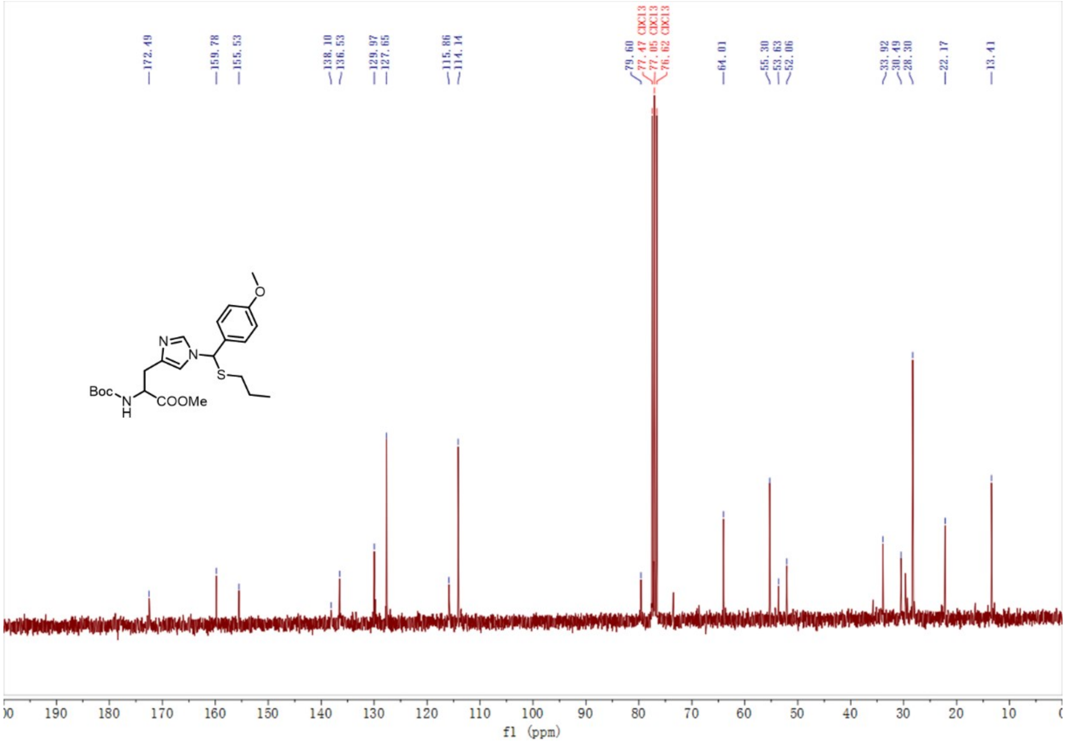

^{13}C NMR spectrum of **3a**

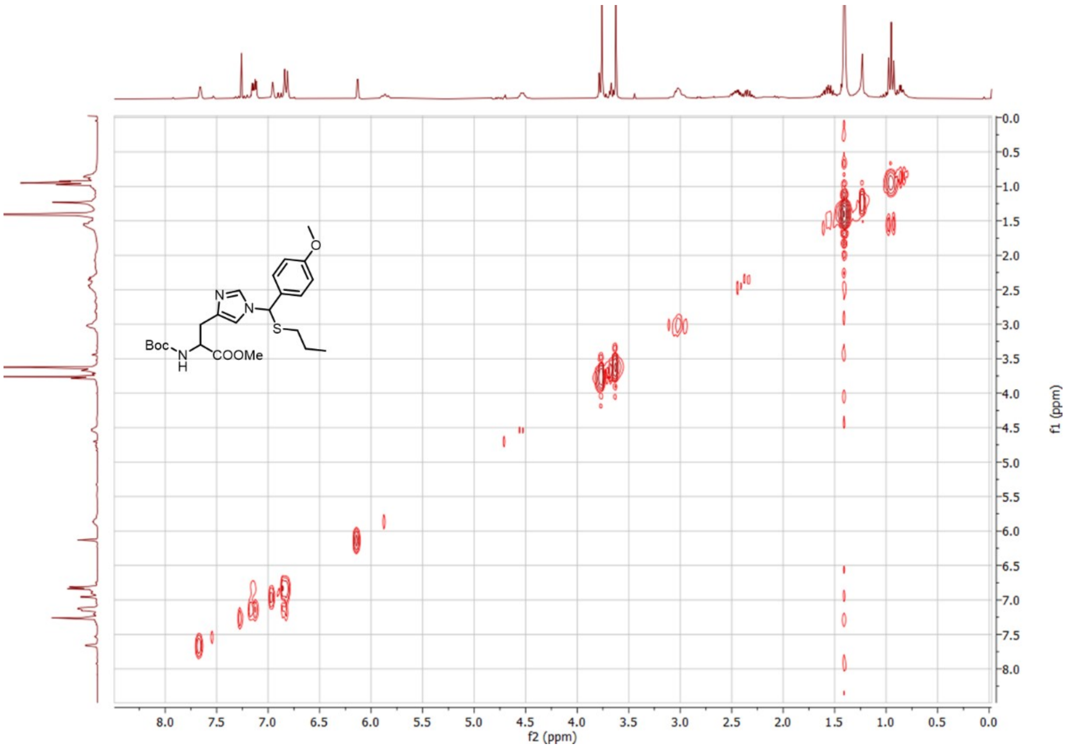

^{1}H-^{1}H COSY NMR spectrum of **3a**

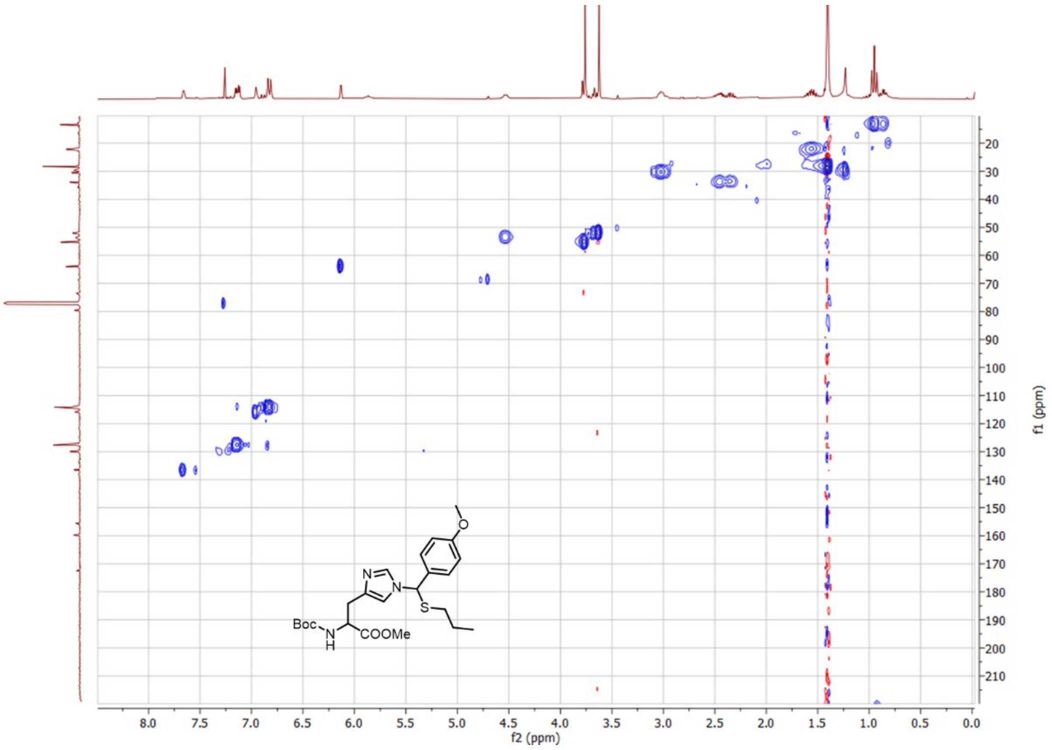

^{1}H-^{13}C HSQC NMR spectrum of **3a**

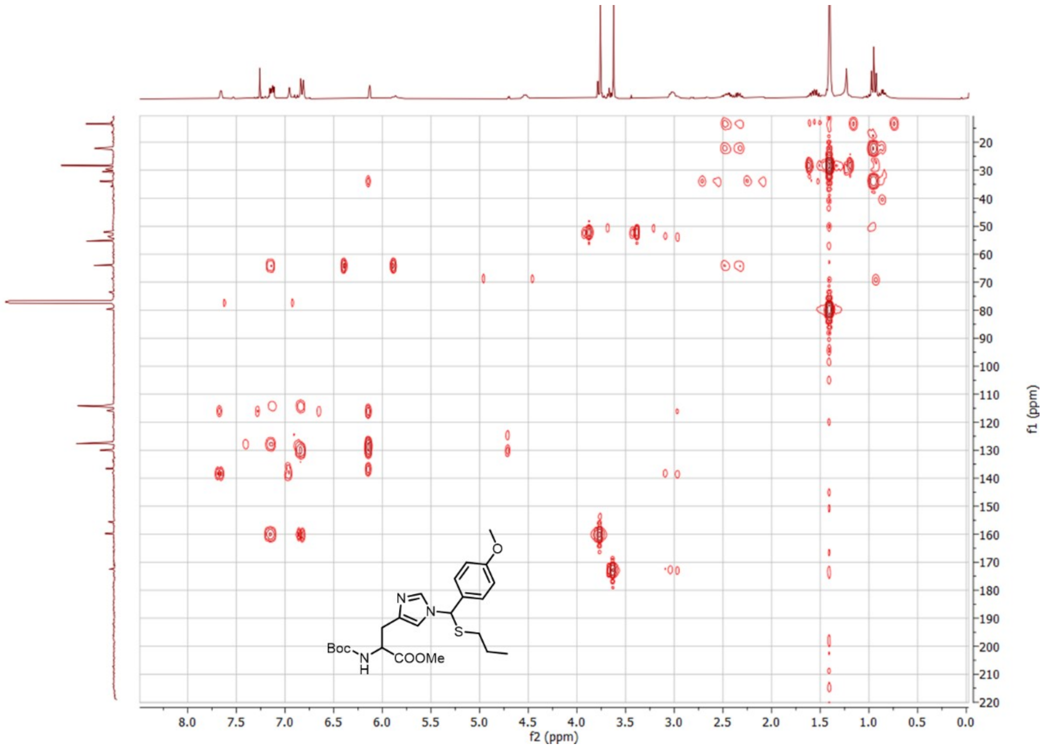

^{1}H-^{13}C HMBC NMR spectrum of **3a**

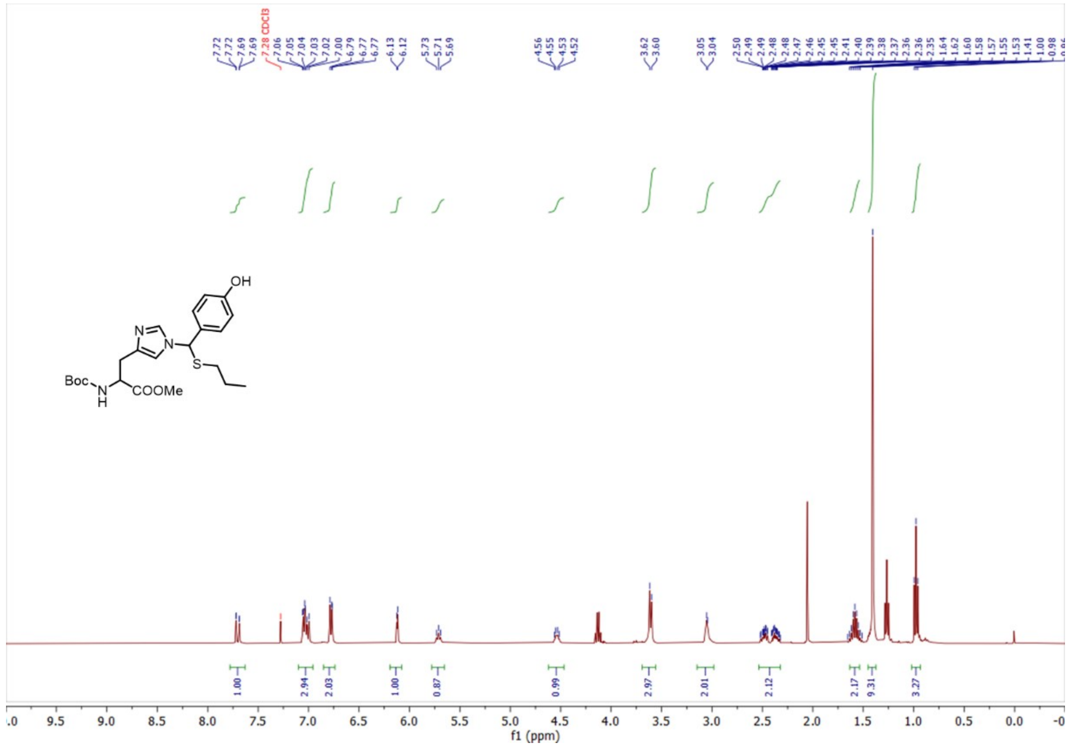

^{1}H NMR spectrum of **3b**

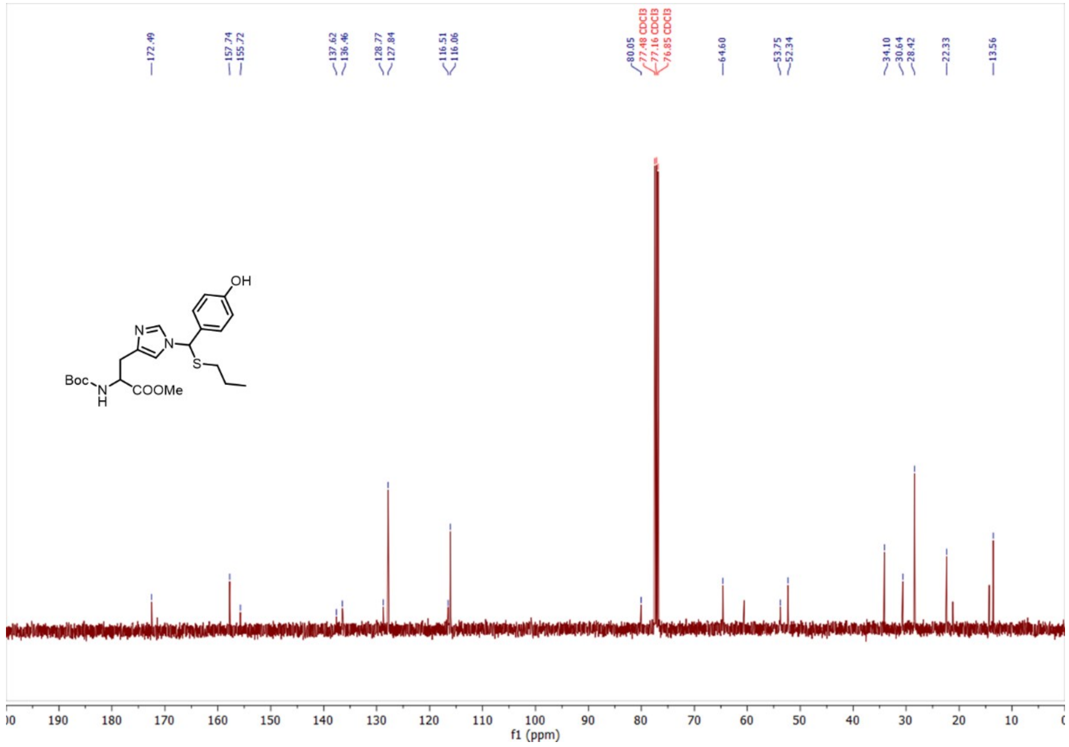

^{13}C NMR spectrum of **3b**

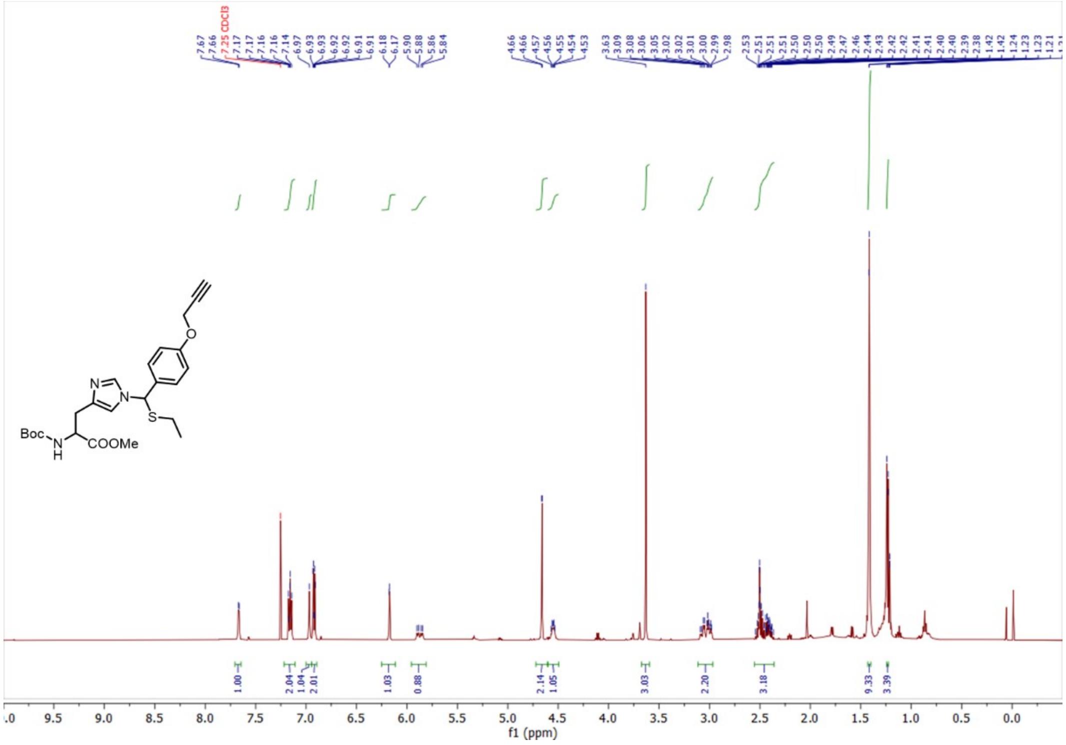

^{1}H NMR spectrum of **3c**

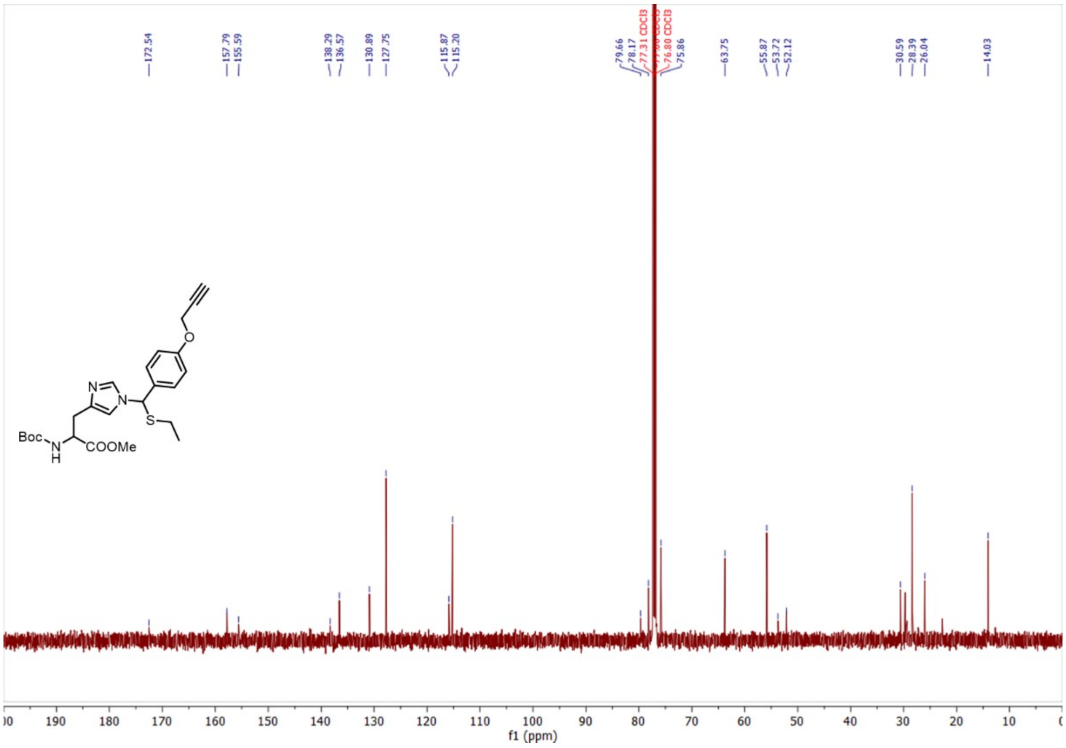

^{13}C NMR spectrum of **3c**

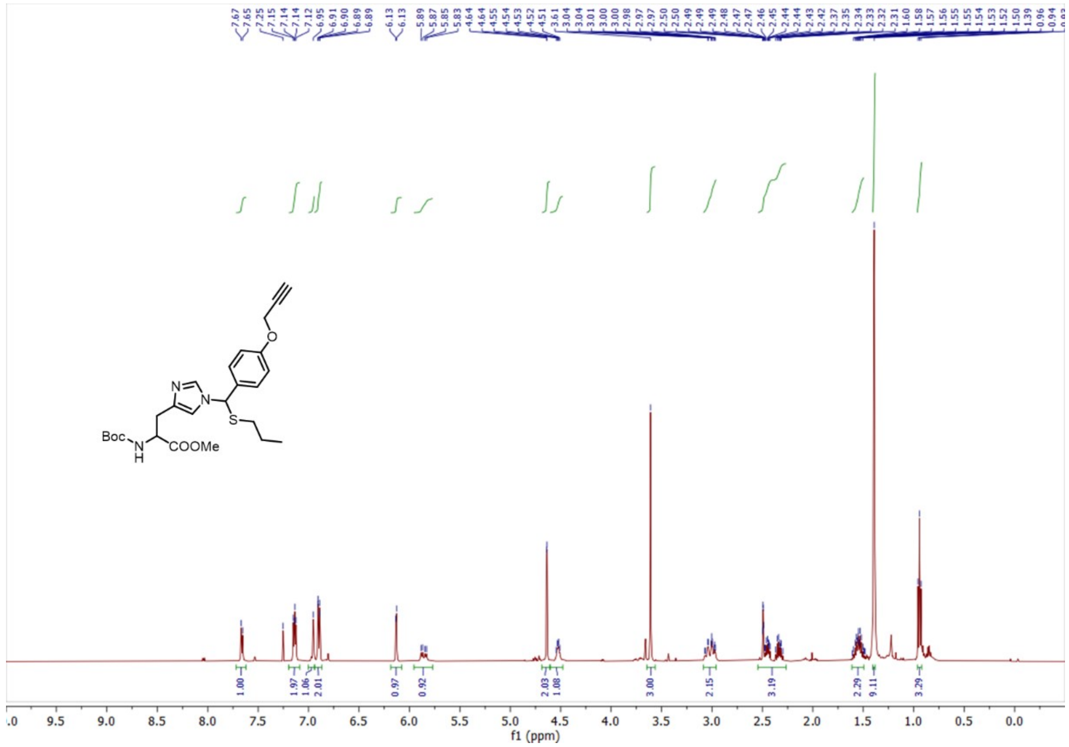

^{1}H NMR spectrum of **3d**

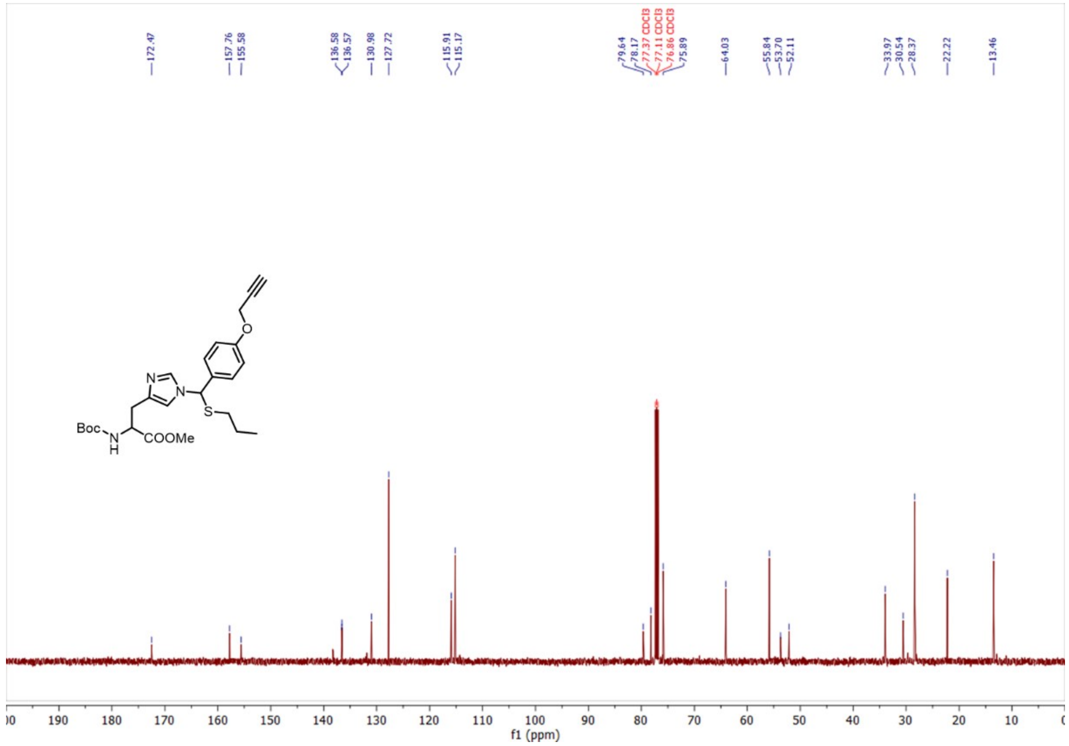

^{13}C NMR spectrum of **3d**

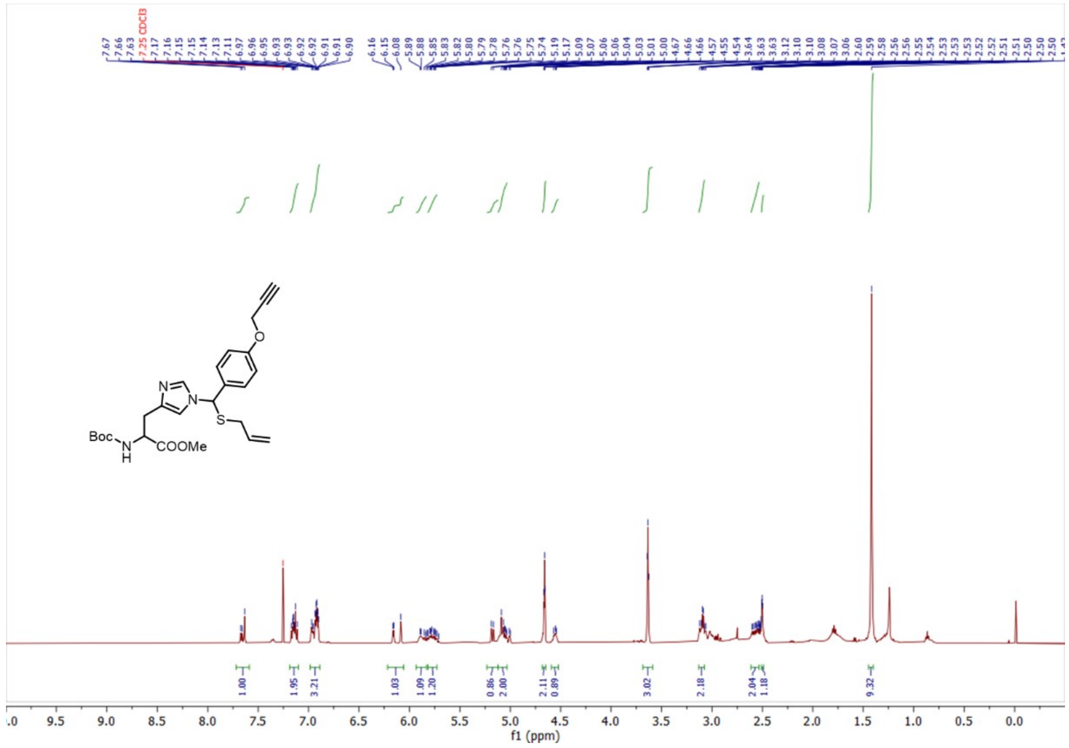

^{1}H NMR spectrum of **3e**

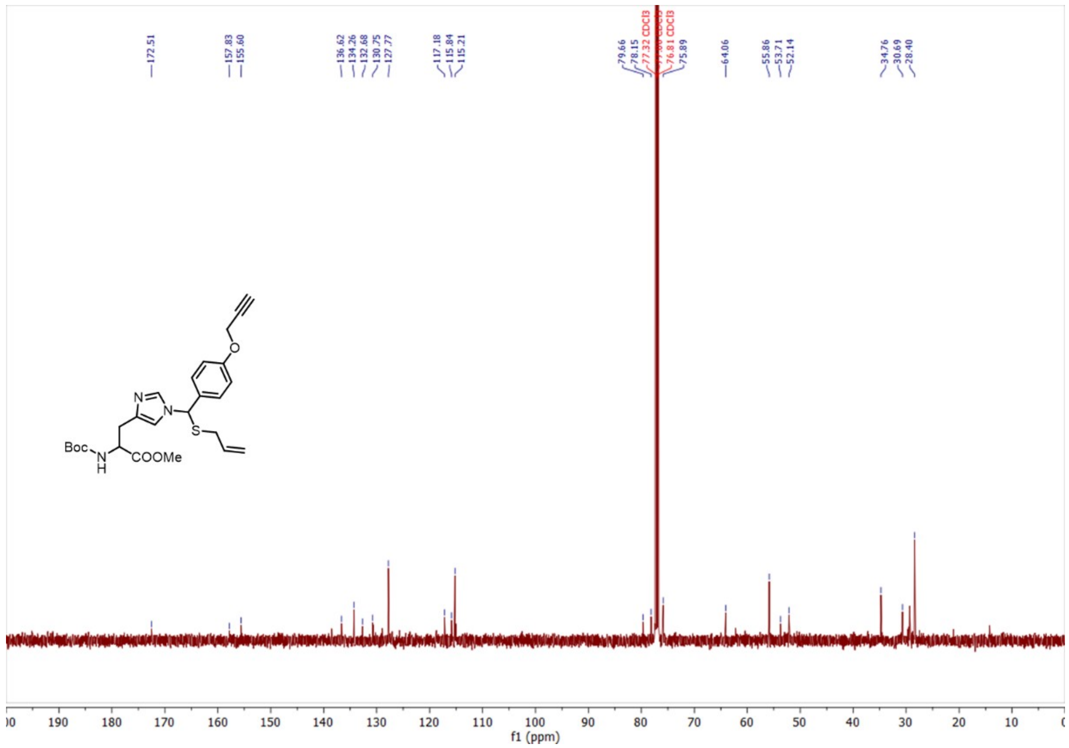

^{13}C NMR spectrum of **3e**

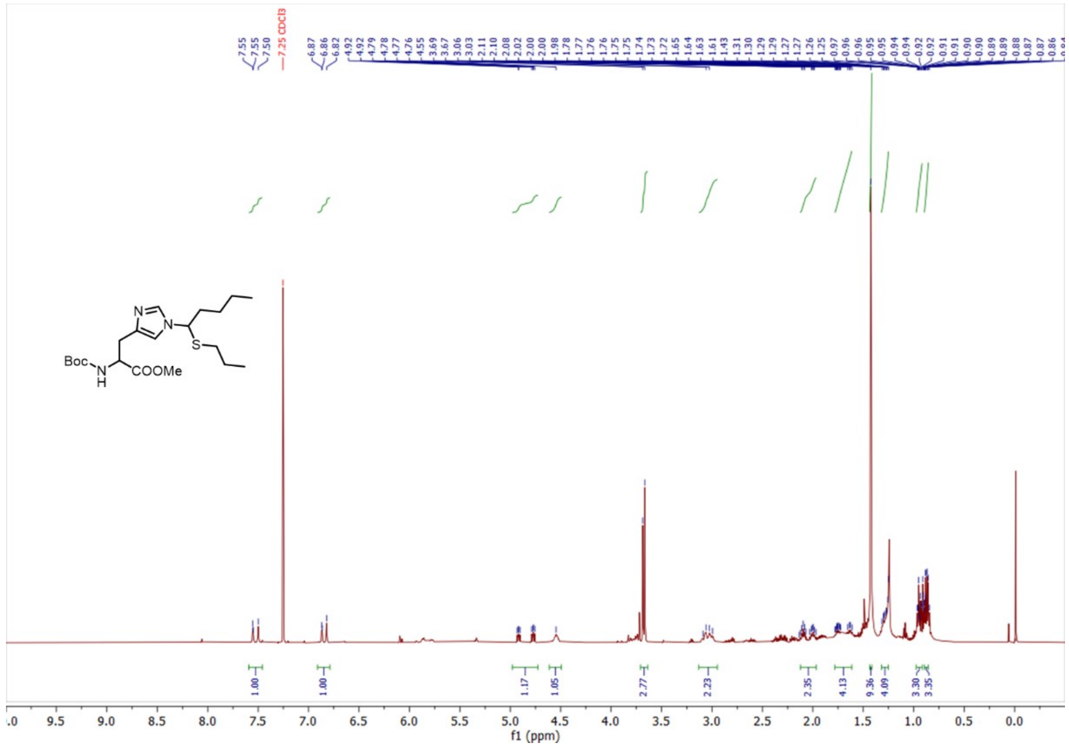

^{1}H NMR spectrum of **3f**

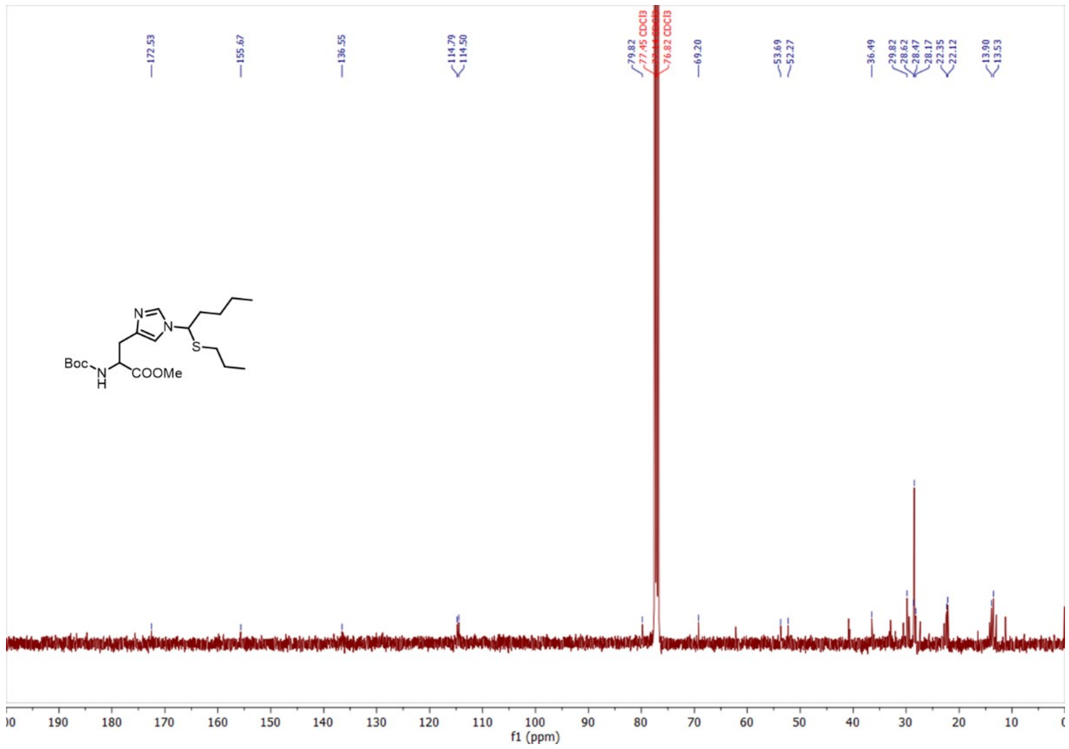

^{13}C NMR spectrum of **3f**

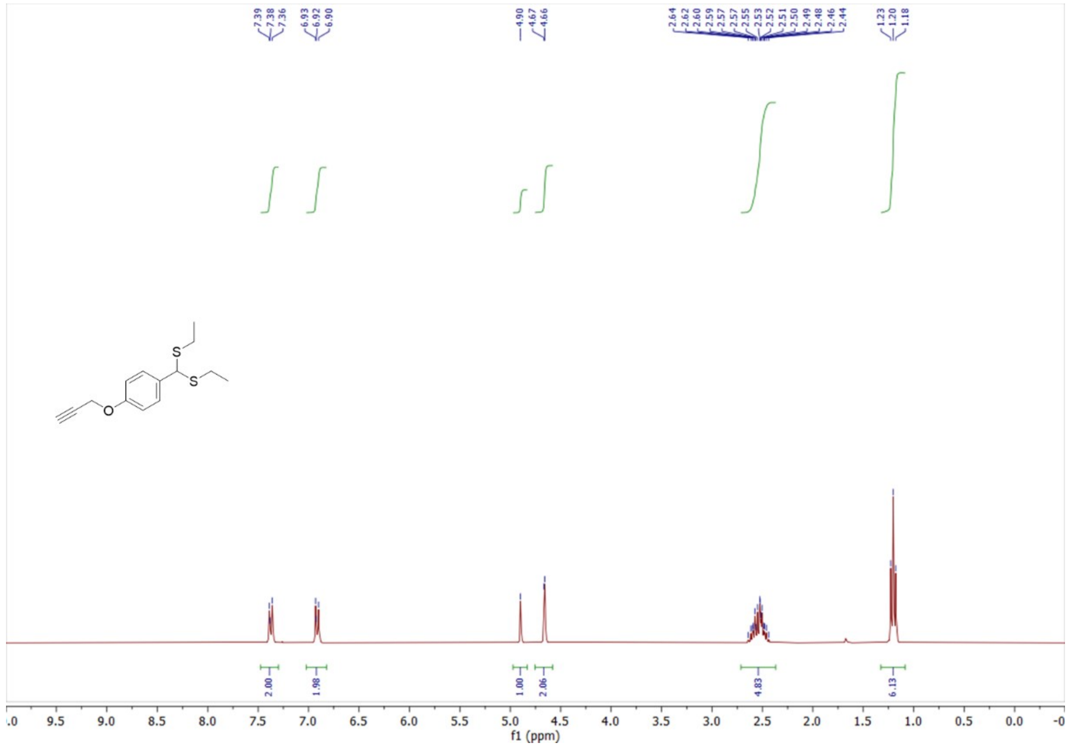

^{1}H NMR spectrum of **TA3**

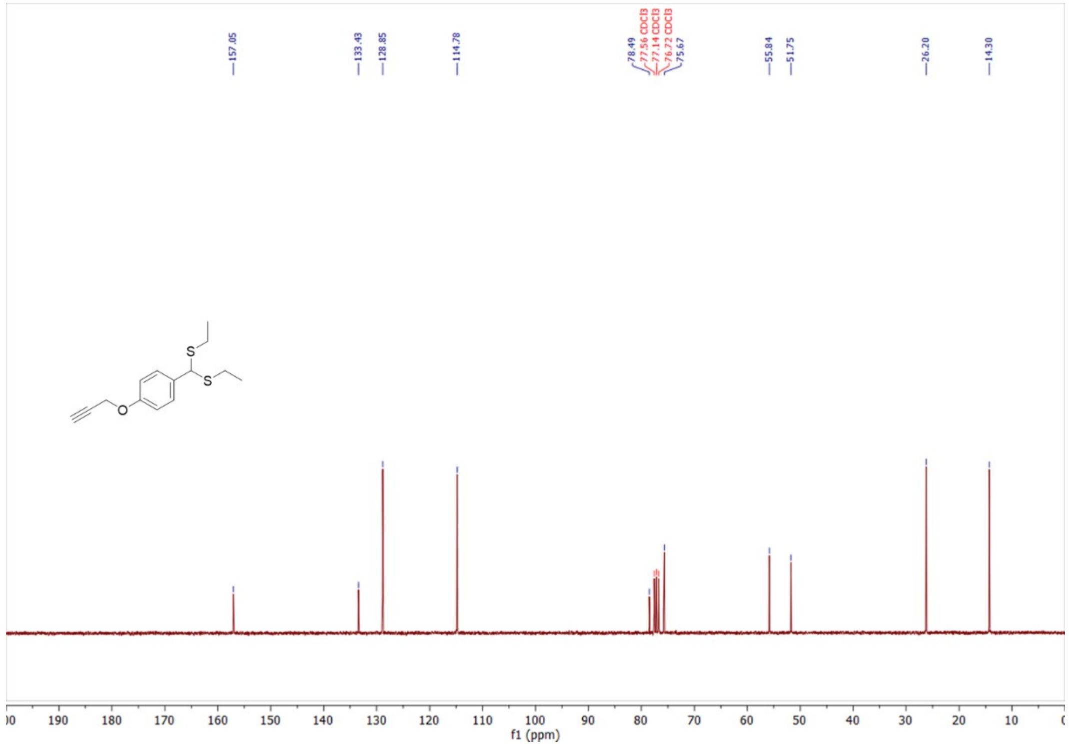

^{13}C NMR spectrum of **TA3**

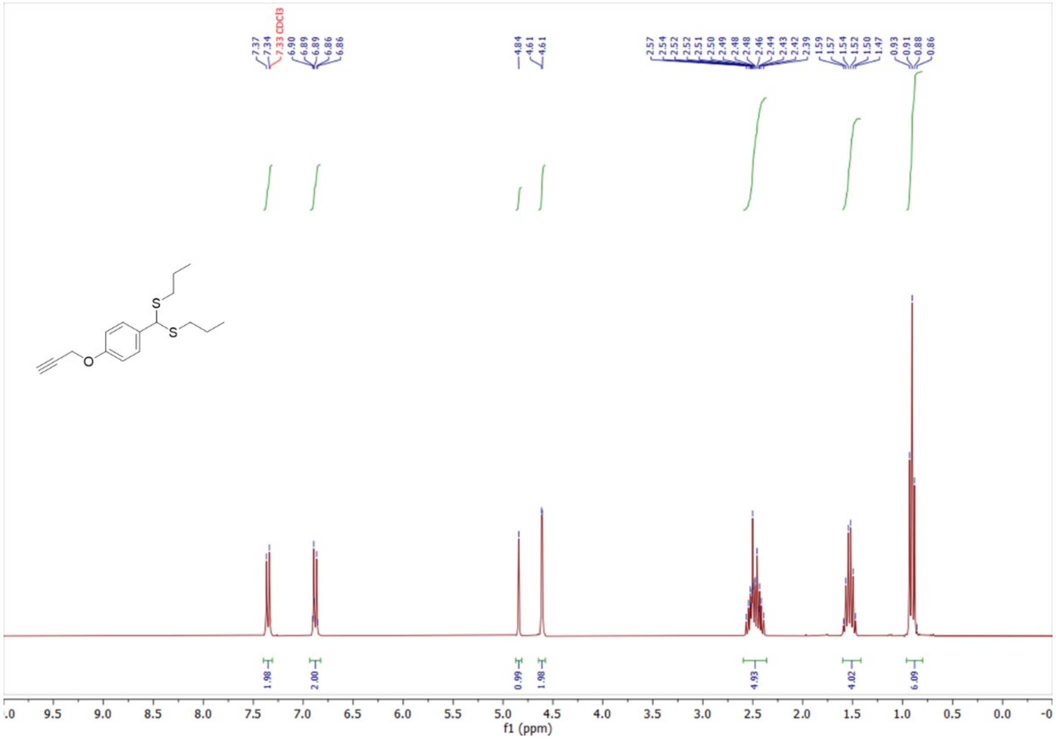

^{1}H NMR spectrum of **TA4**

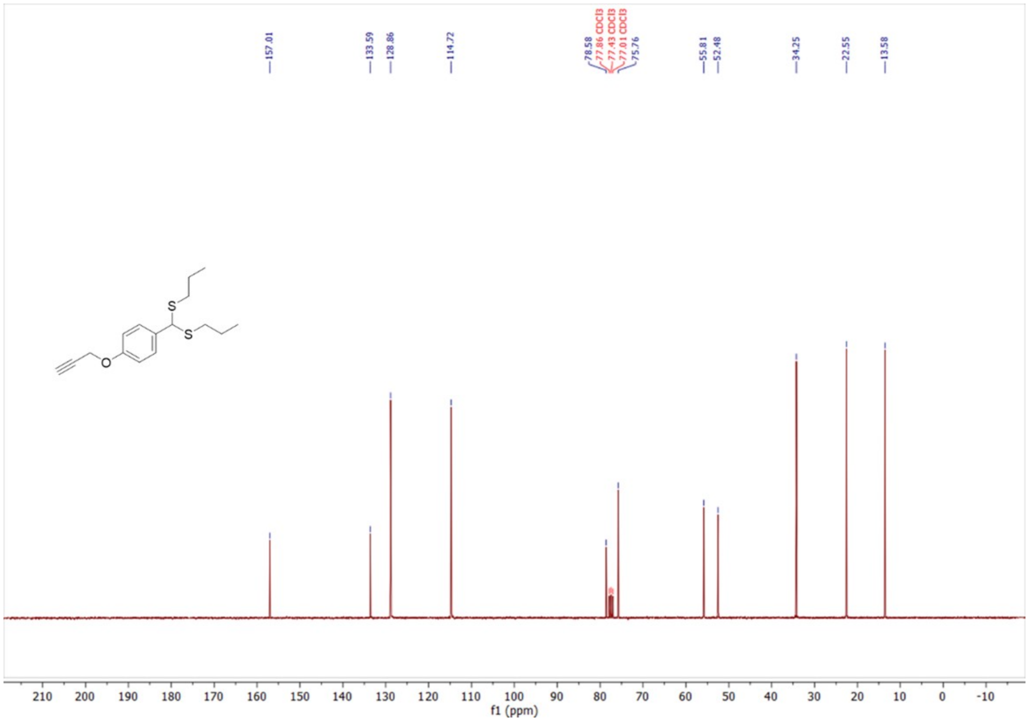

^{13}C NMR spectrum of **TA4**

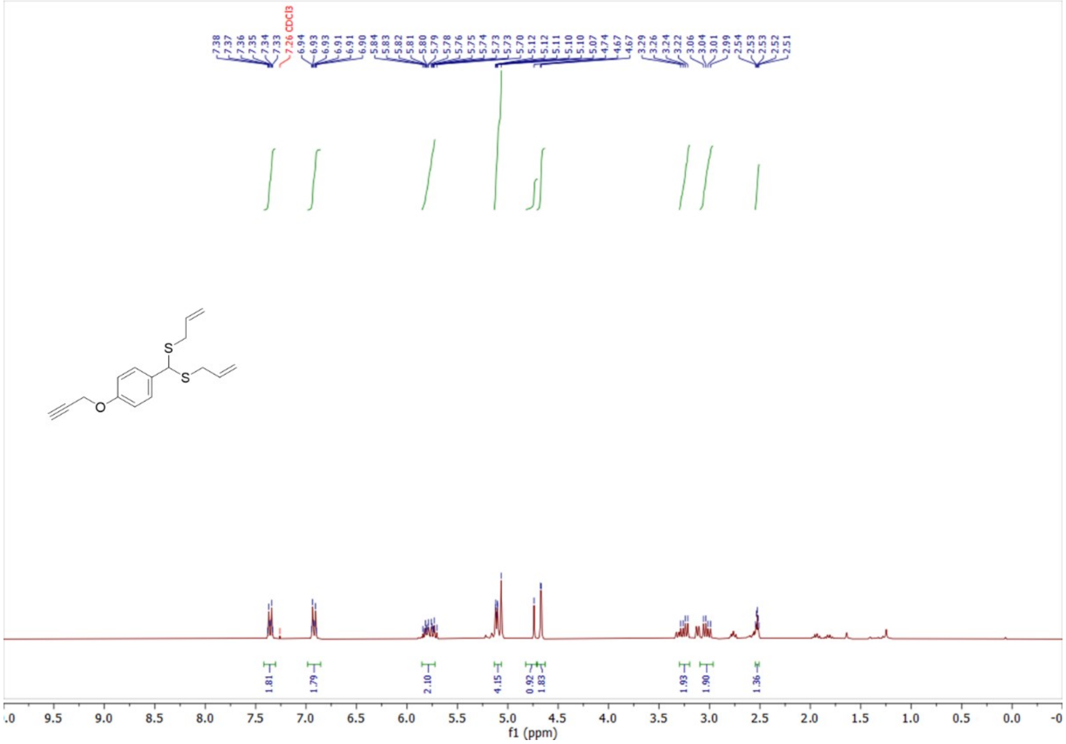

^{1}H NMR spectrum of **TA5**

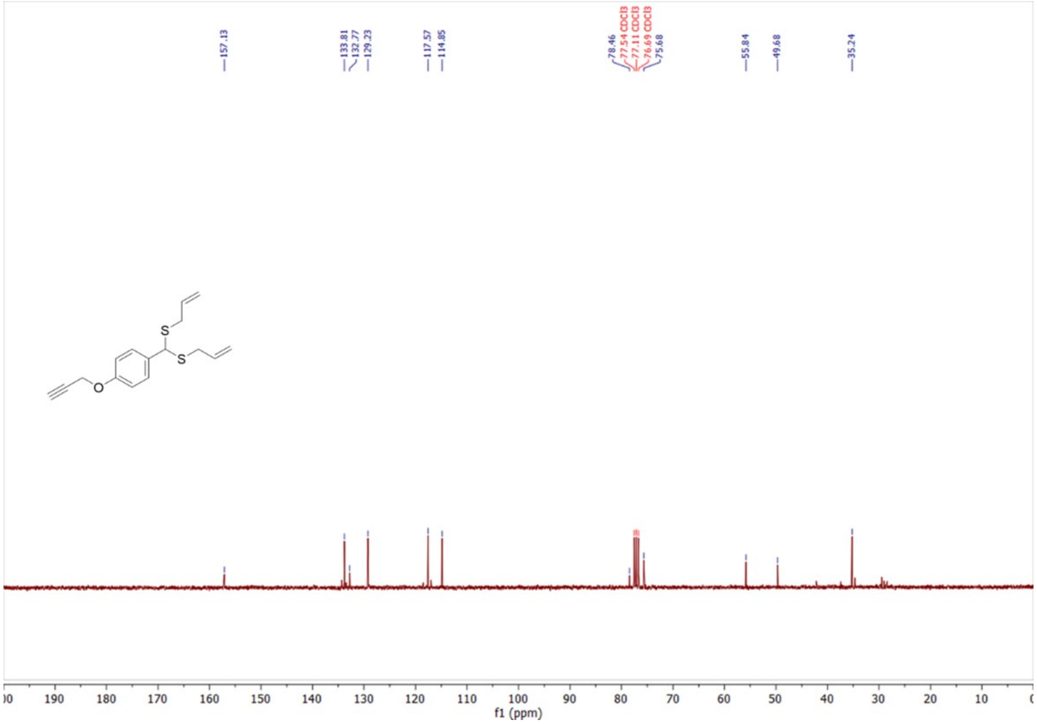

^{13}C NMR spectrum of **TA5**

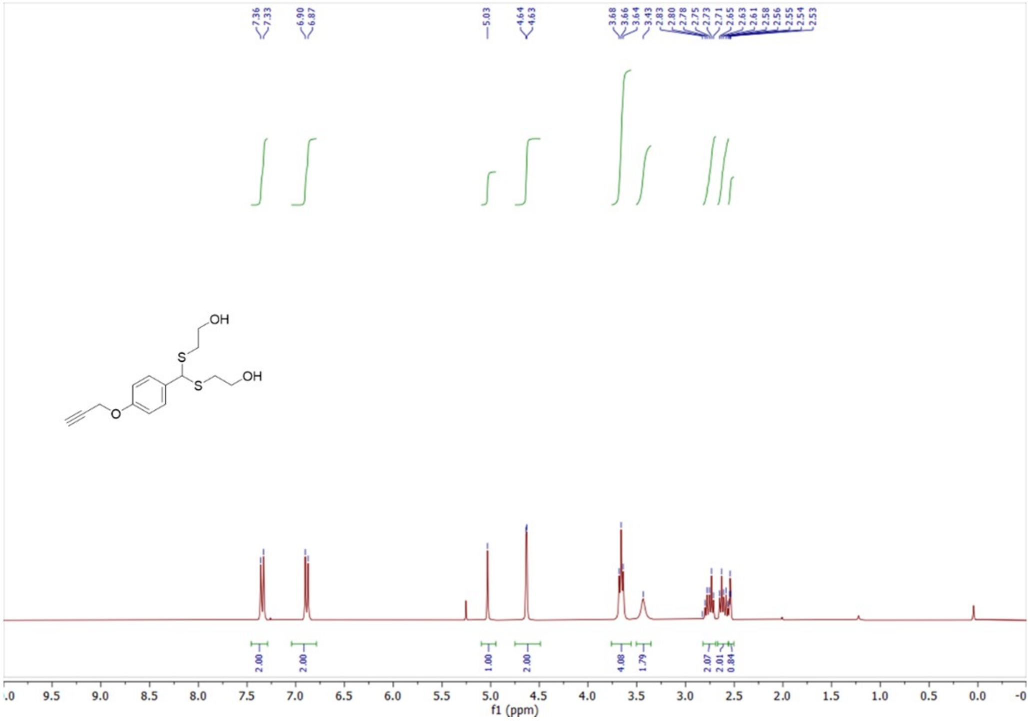

^{1}H NMR spectrum of **TA6**

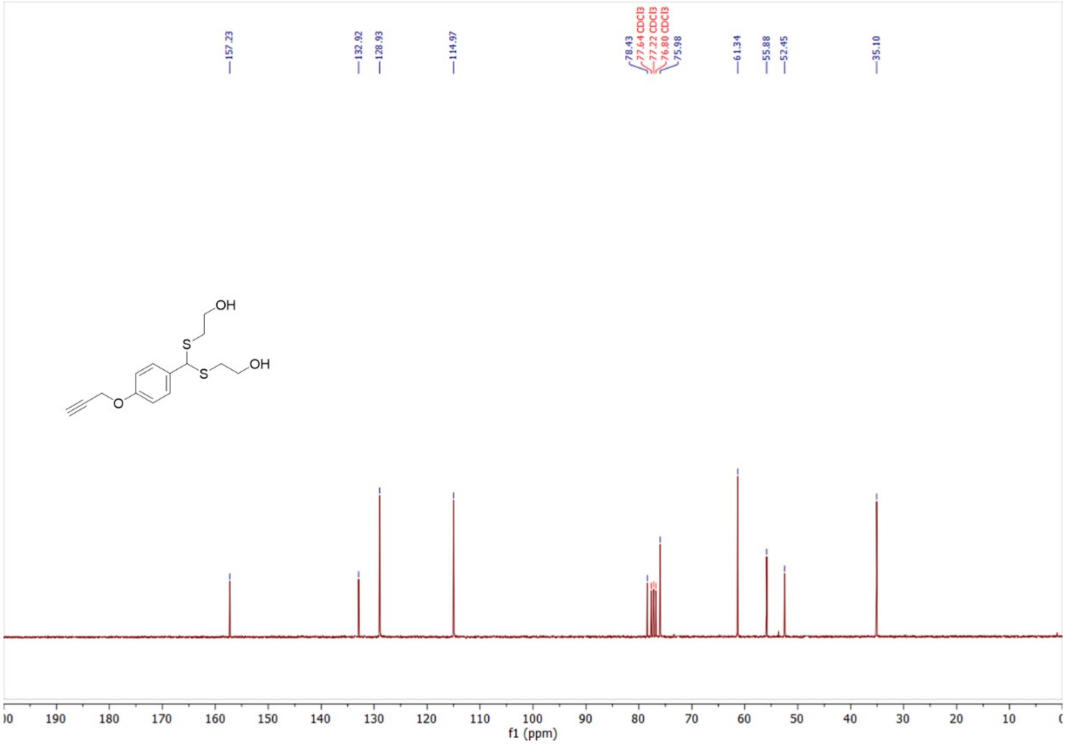

^{13}C NMR spectrum of **TA6**

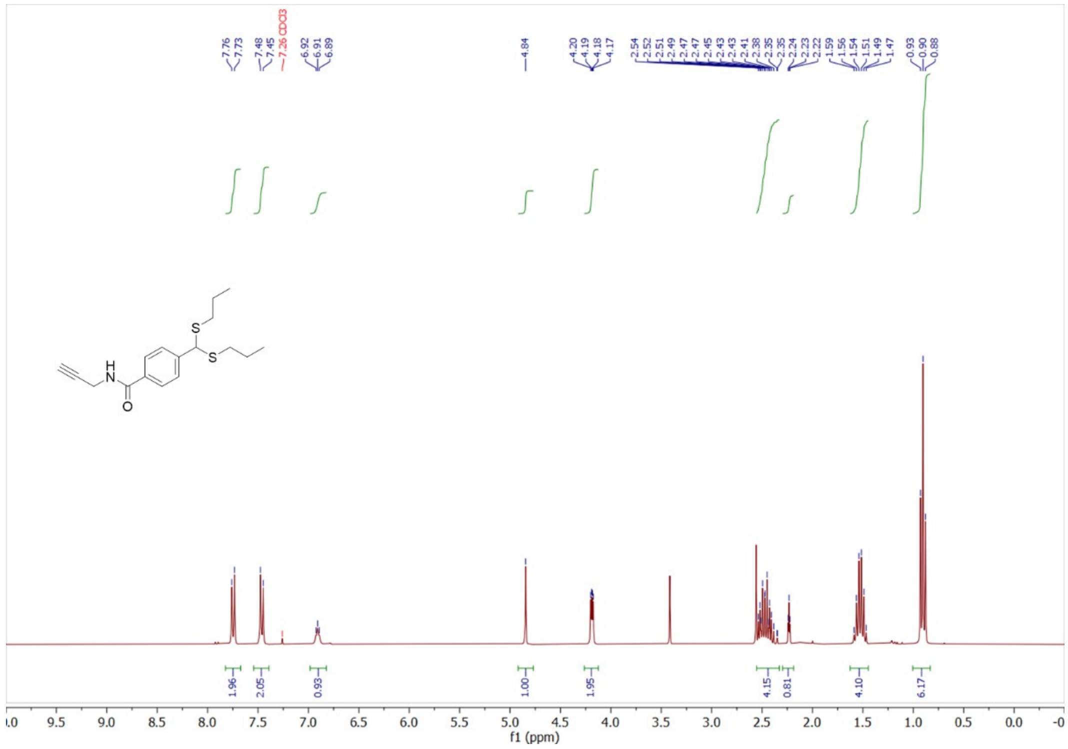

^{1}H NMR spectrum of **TA7**

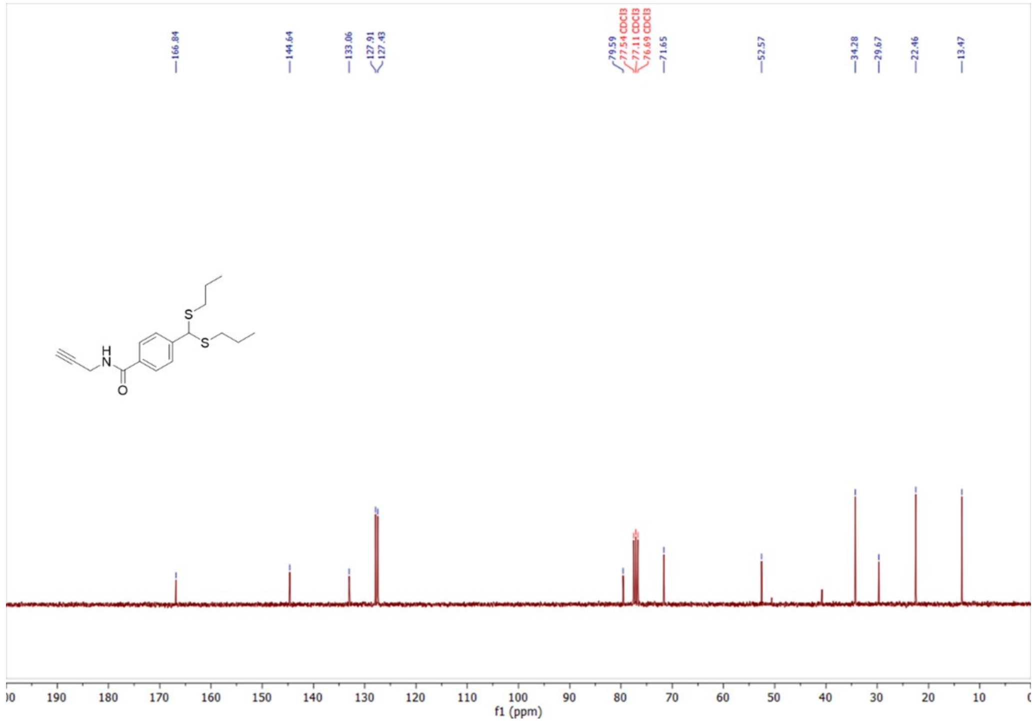

^{13}C NMR spectrum of **TA7**

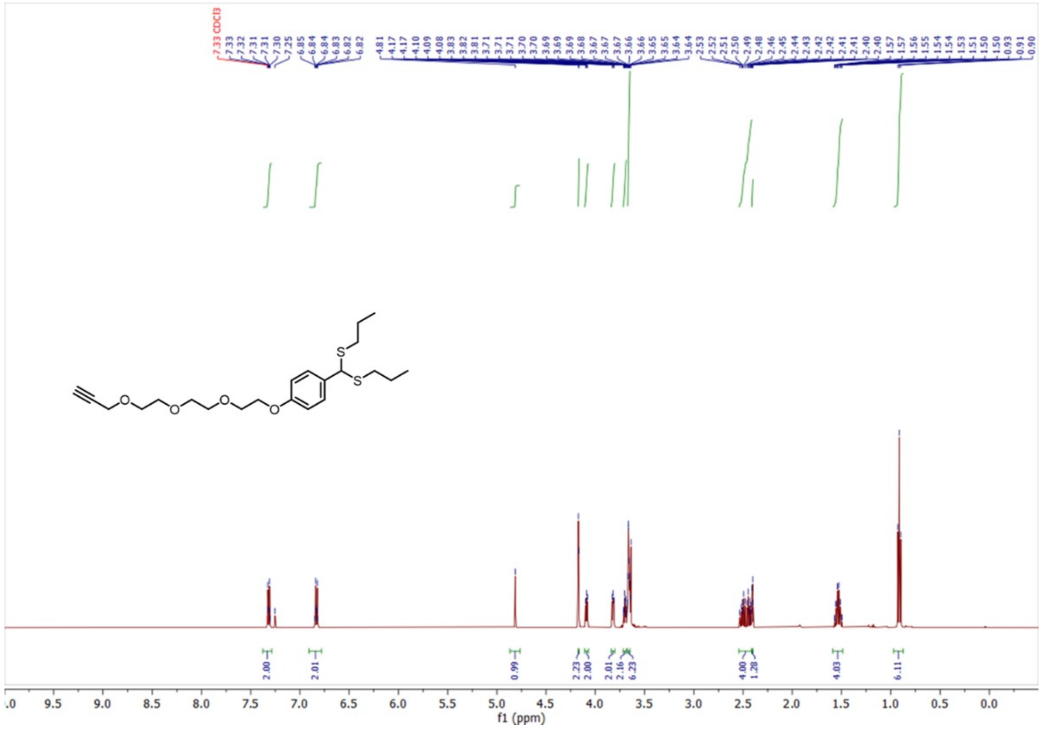

^{1}H NMR spectrum of **TA8**

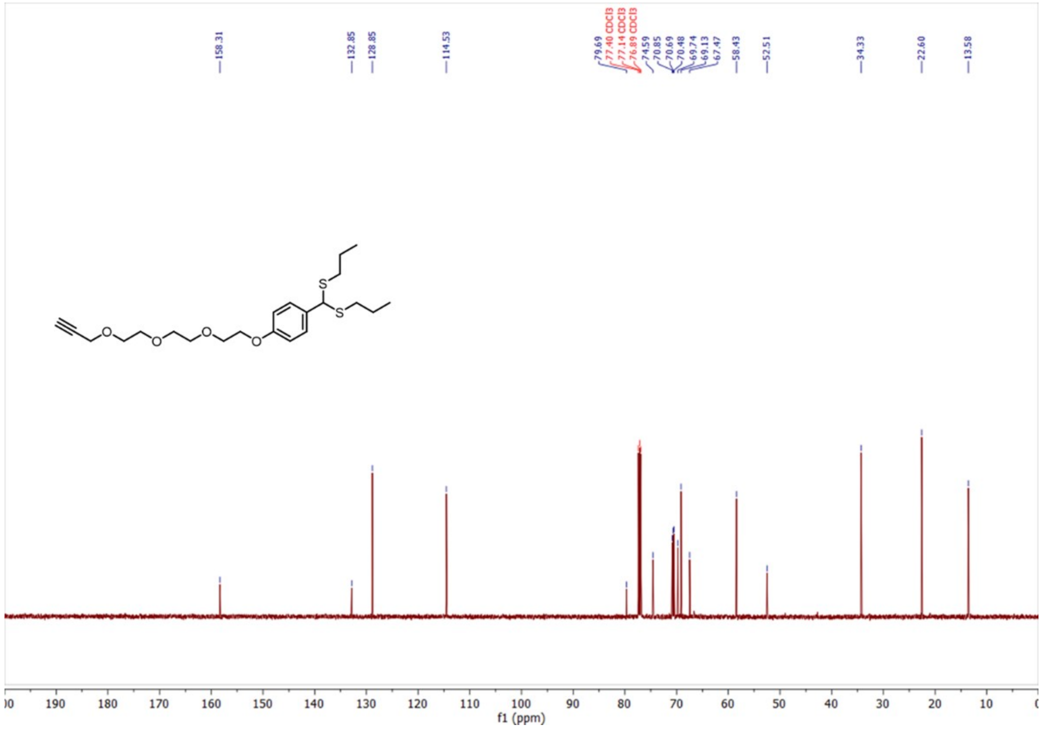

^{13}C NMR spectrum of **TA8**

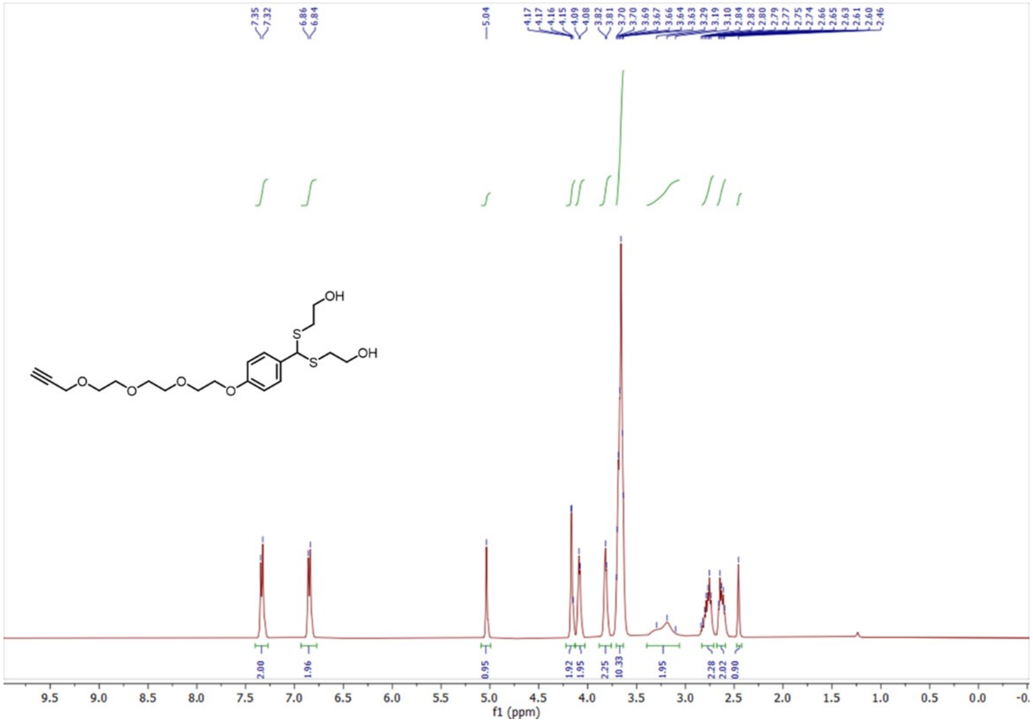

^1H NMR spectrum of **TA9**

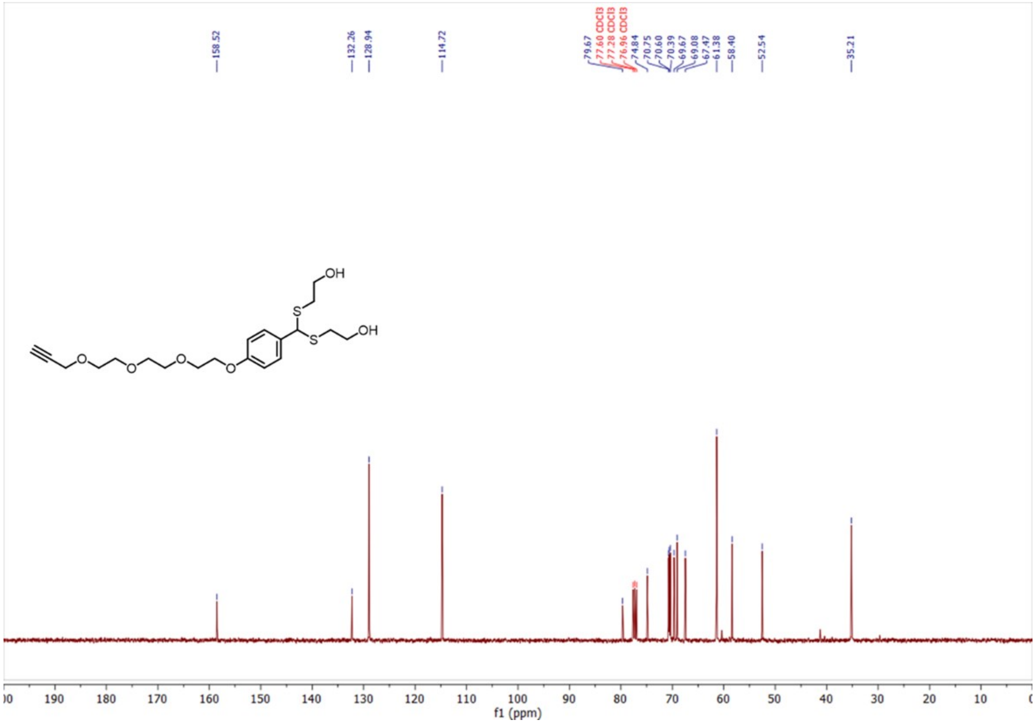

^{13}C NMR spectrum of **TA9**

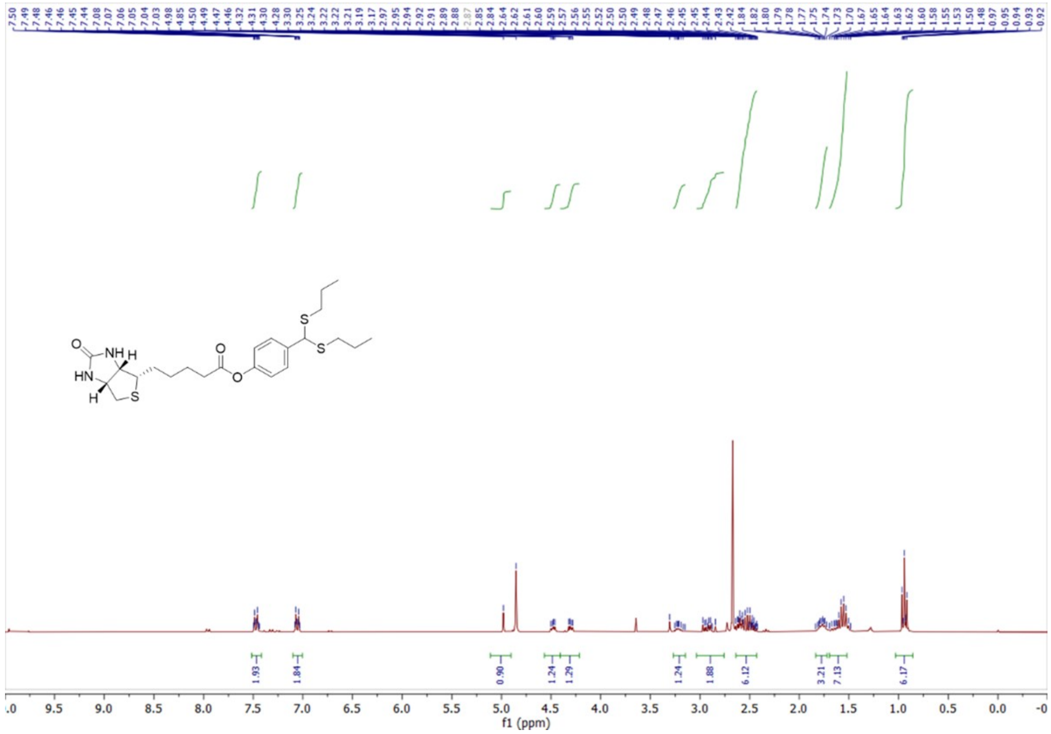

^{1}H NMR spectrum of **TA1**

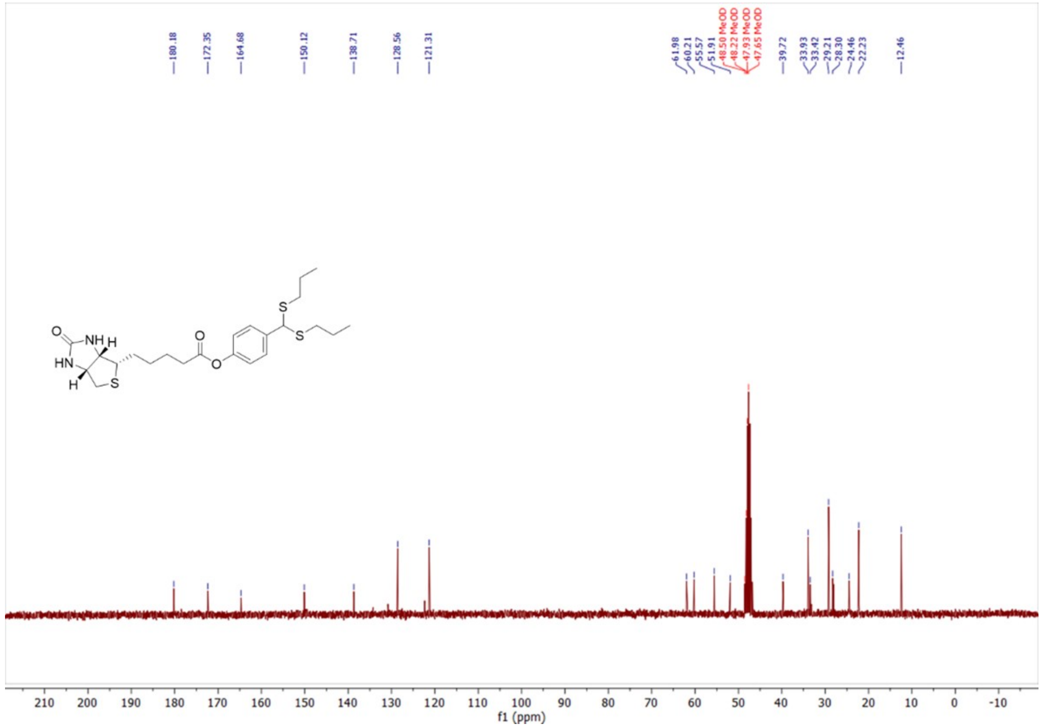

^{13}C NMR spectrum of **TA1**

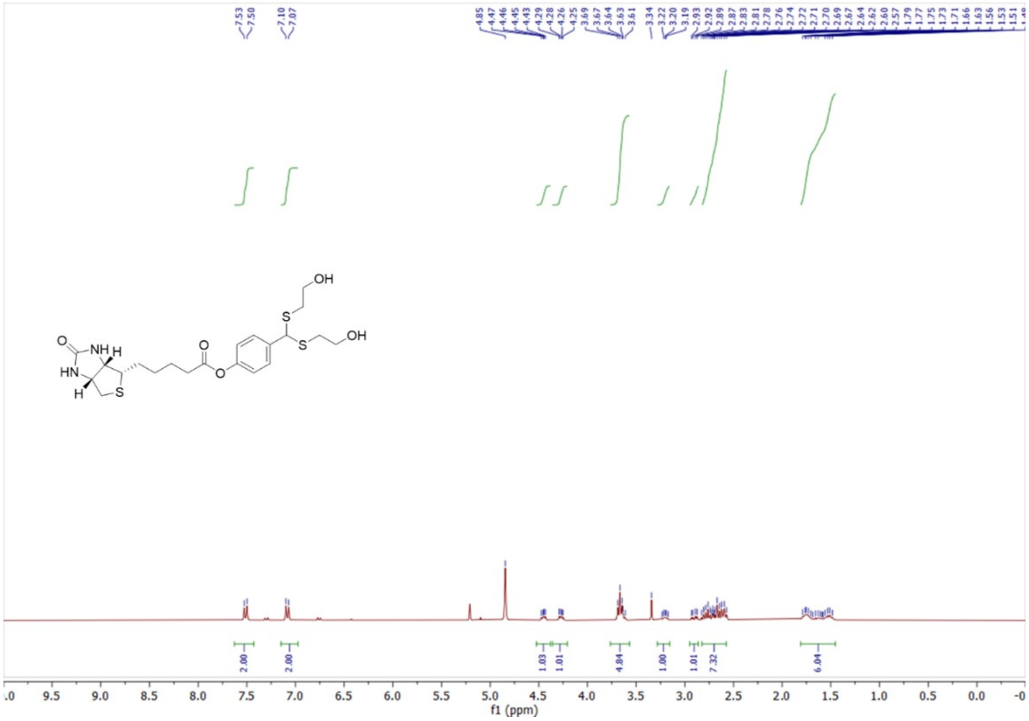

^{1}H NMR spectrum of **TA10**

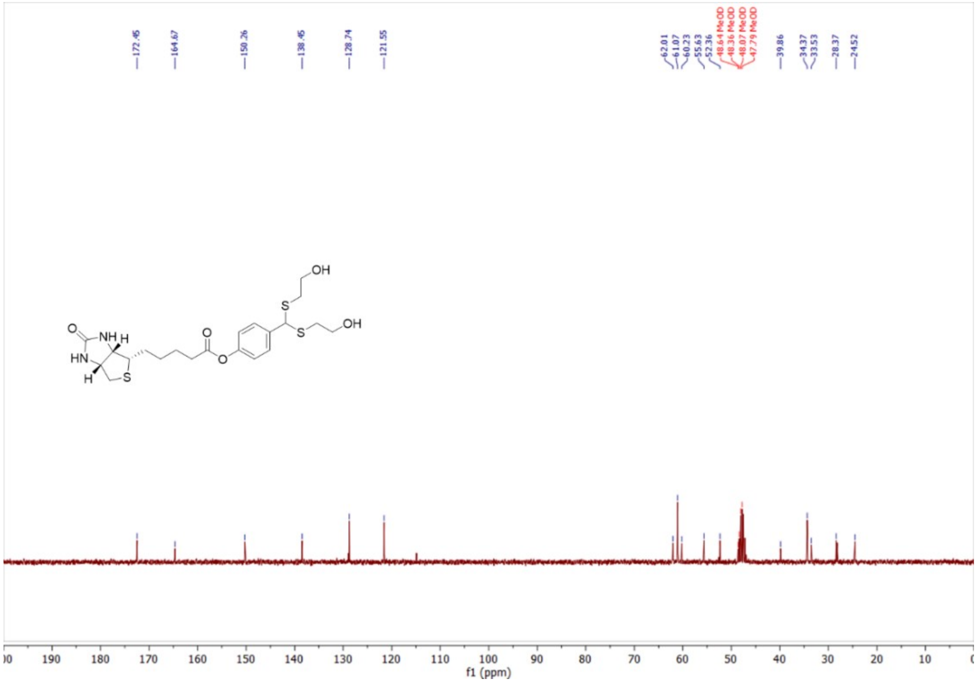

^{13}C NMR spectrum of **TA10**

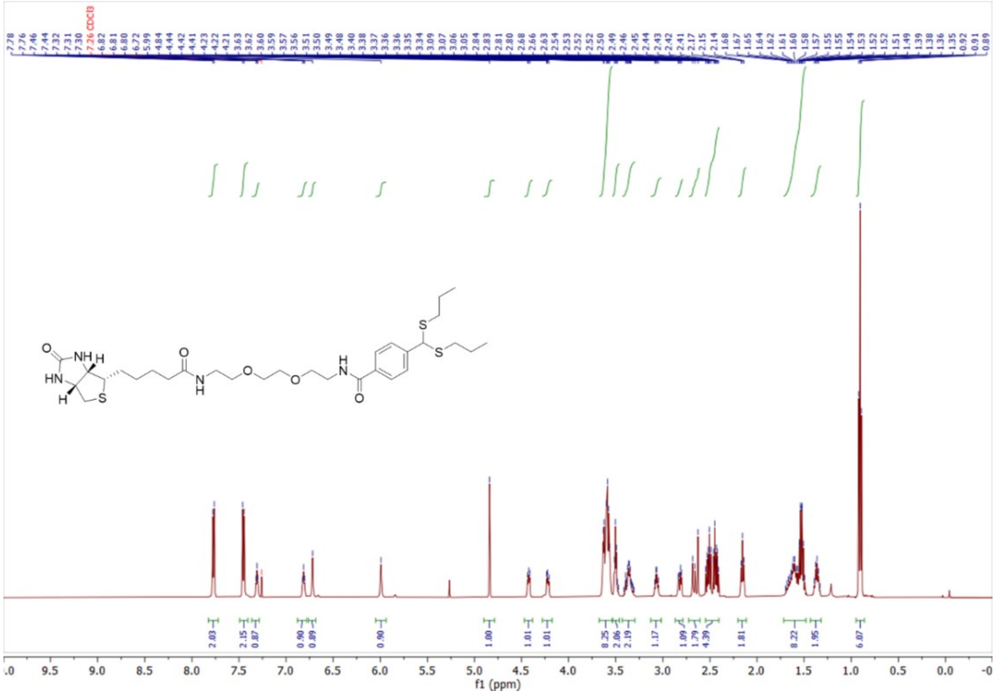

^{1}H NMR spectrum of **TA2**

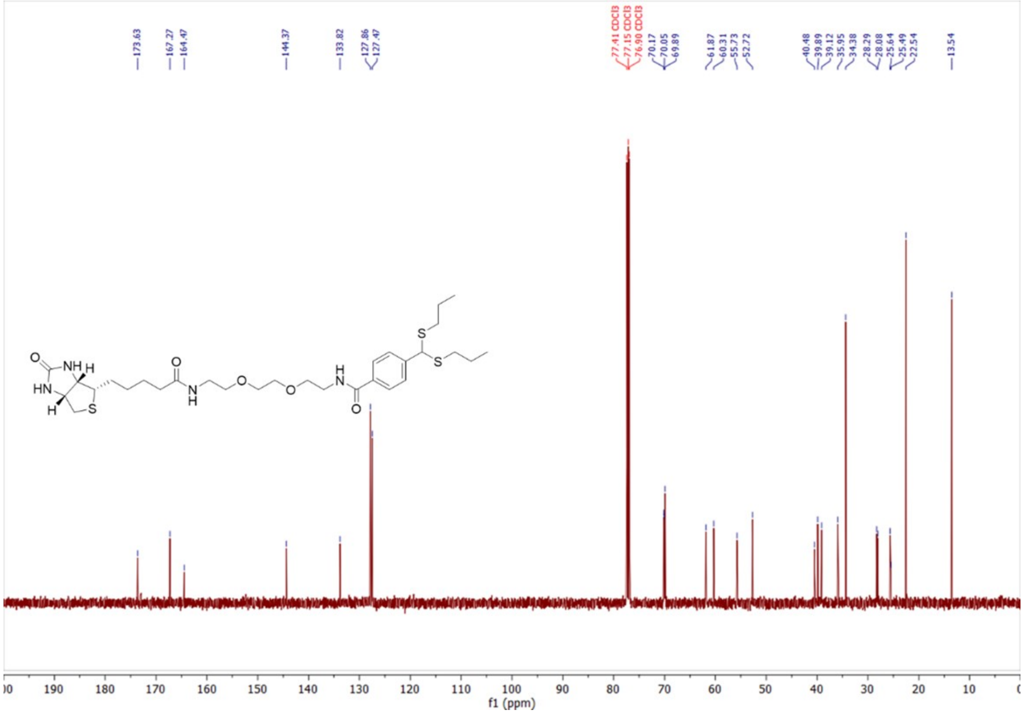

^{13}C NMR spectrum of **TA2**

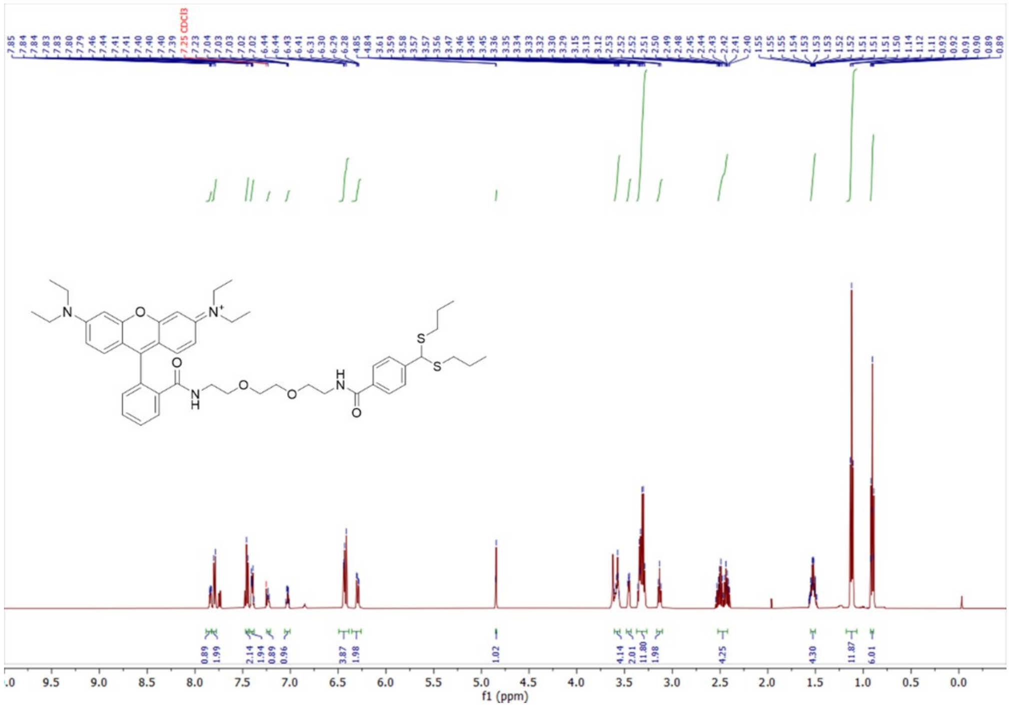

^{1}H NMR spectrum of **TA11**

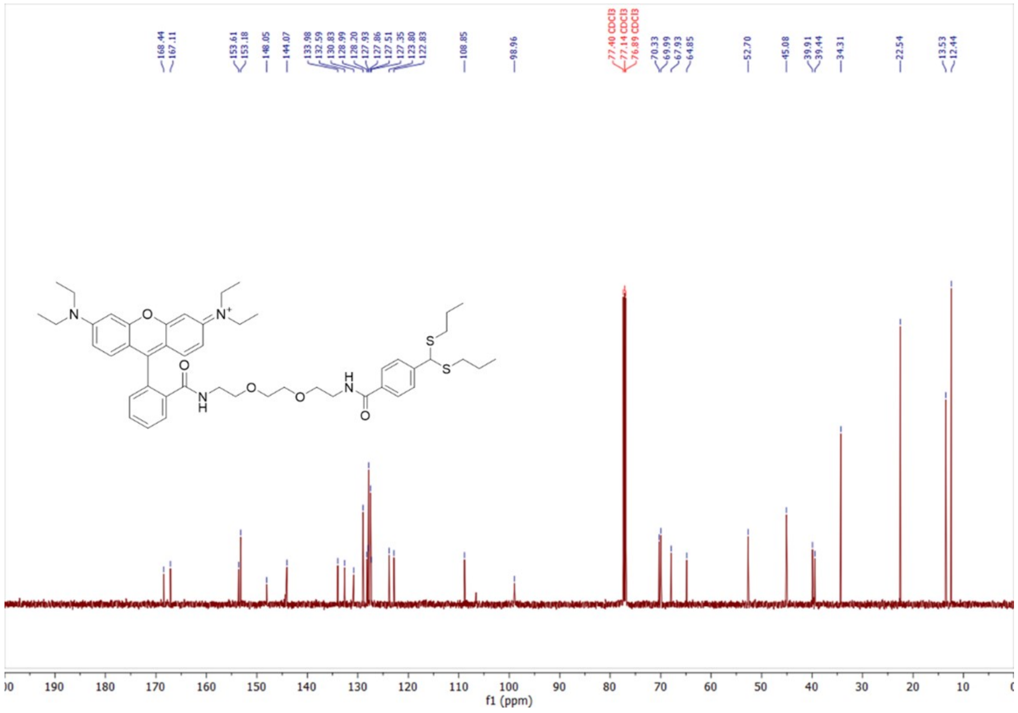

^{13}C NMR spectrum of **TA11**

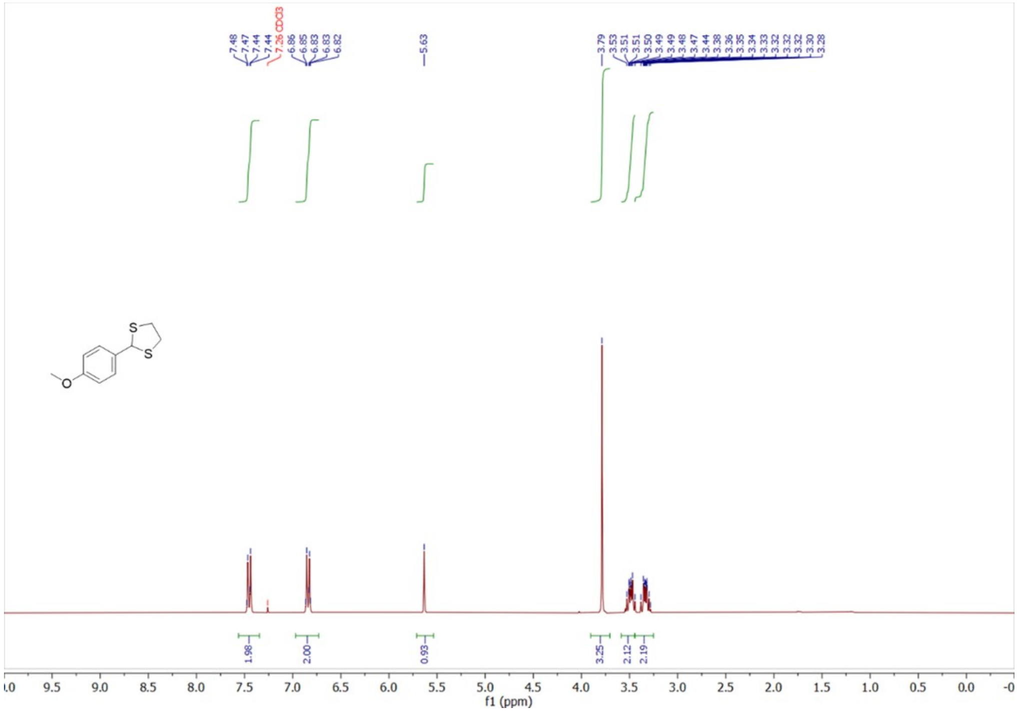

^{1}H NMR spectrum of **S1**

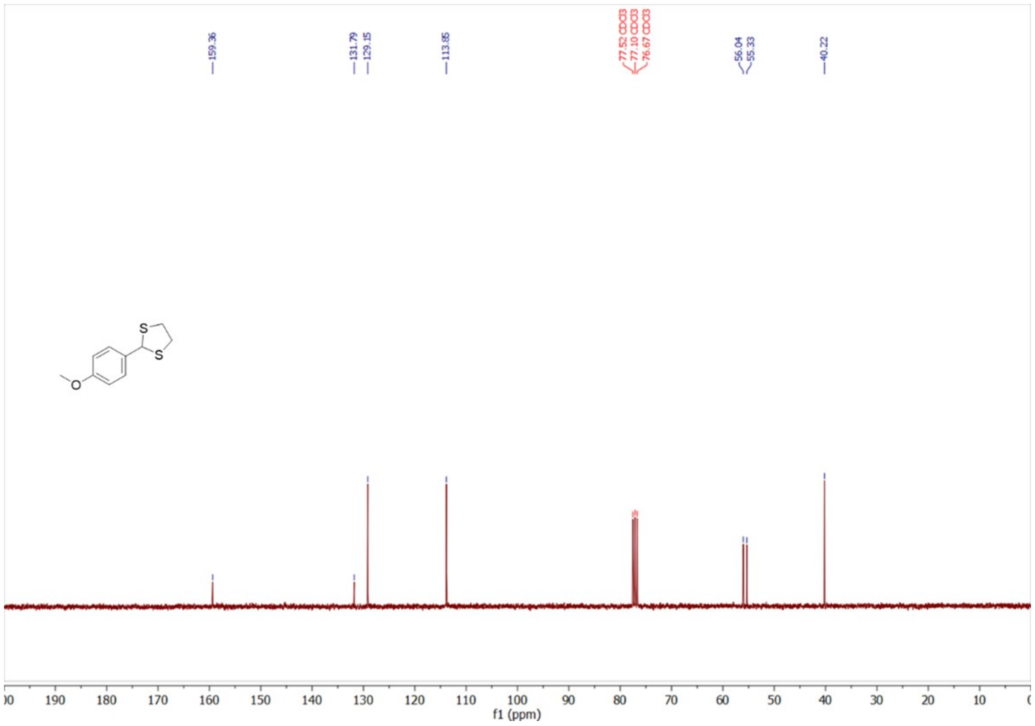

^{13}C NMR spectrum of **S1**

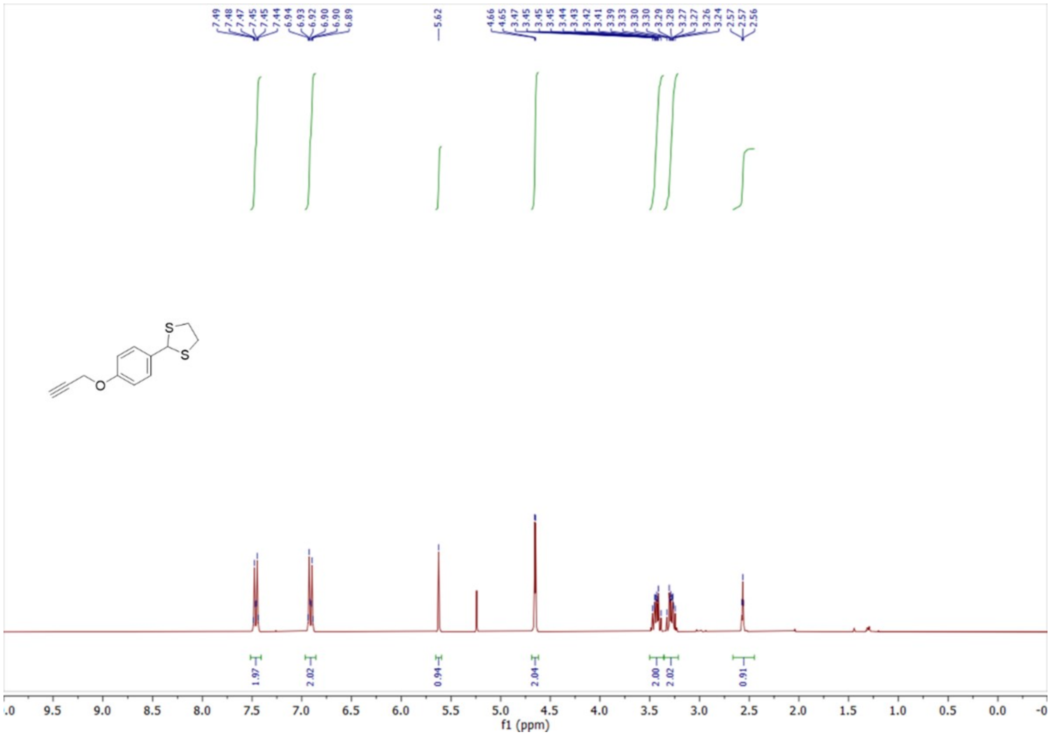

^{1}H NMR spectrum of **S2**

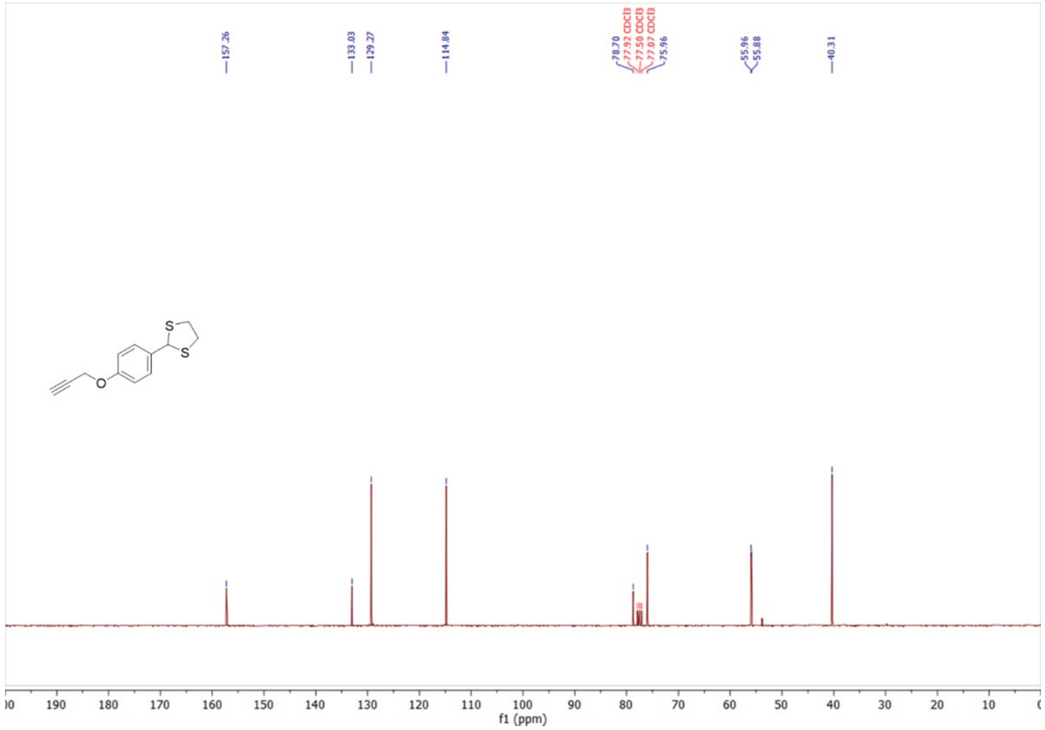

^{13}C NMR spectrum of **S2**

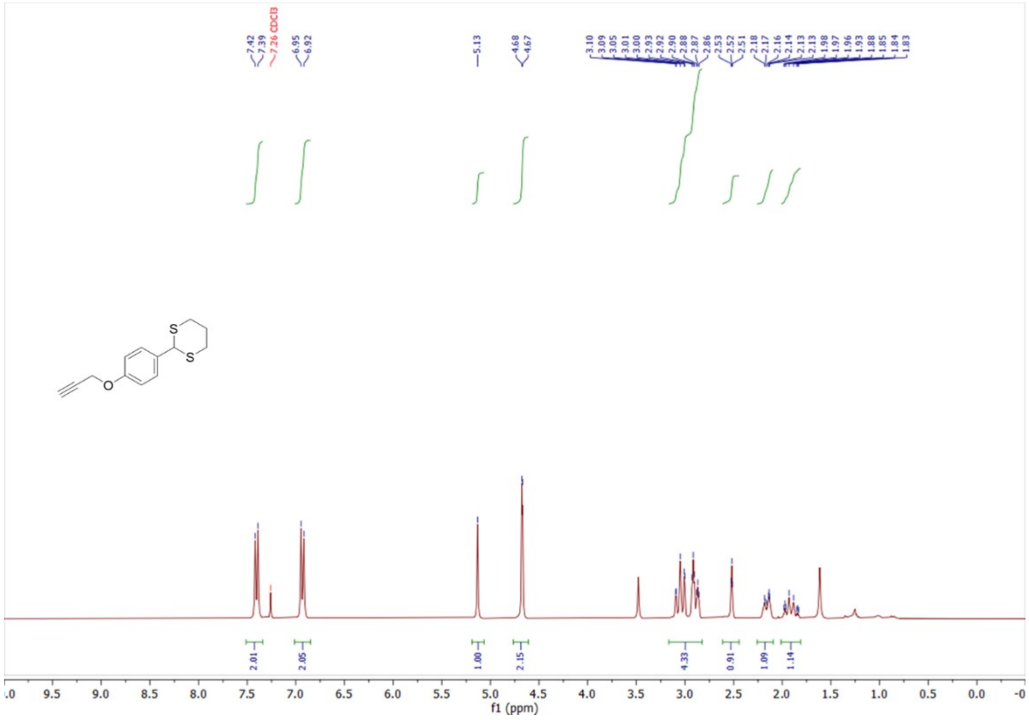

^{1}H NMR spectrum of **S3**

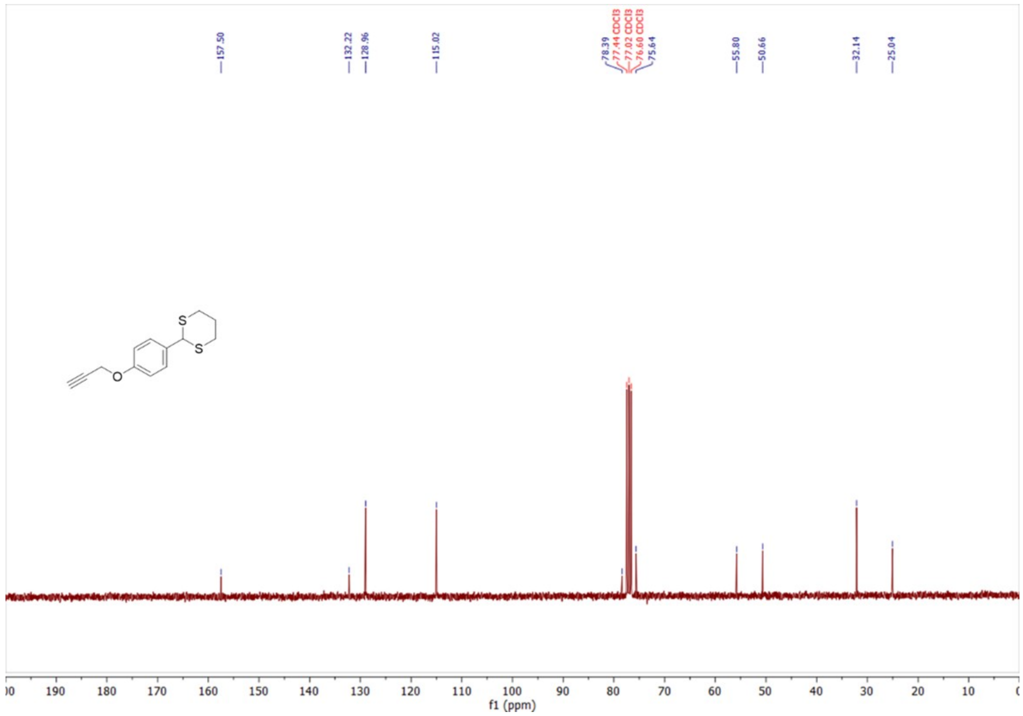

^{13}C NMR spectrum of **S3**

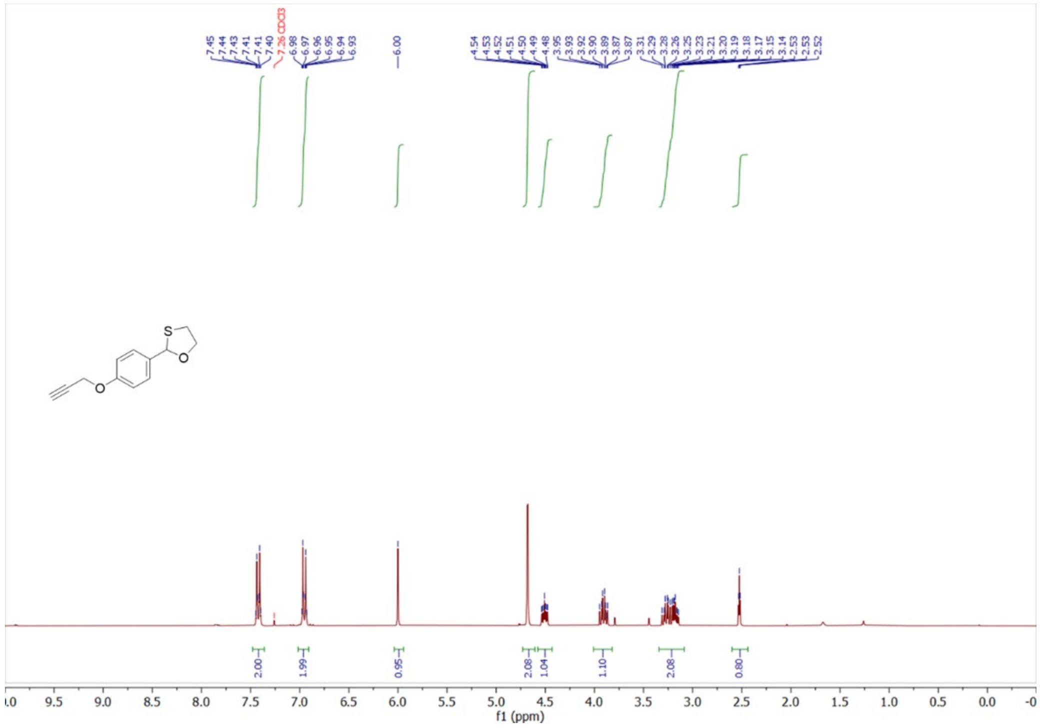

^1H NMR spectrum of **S4**

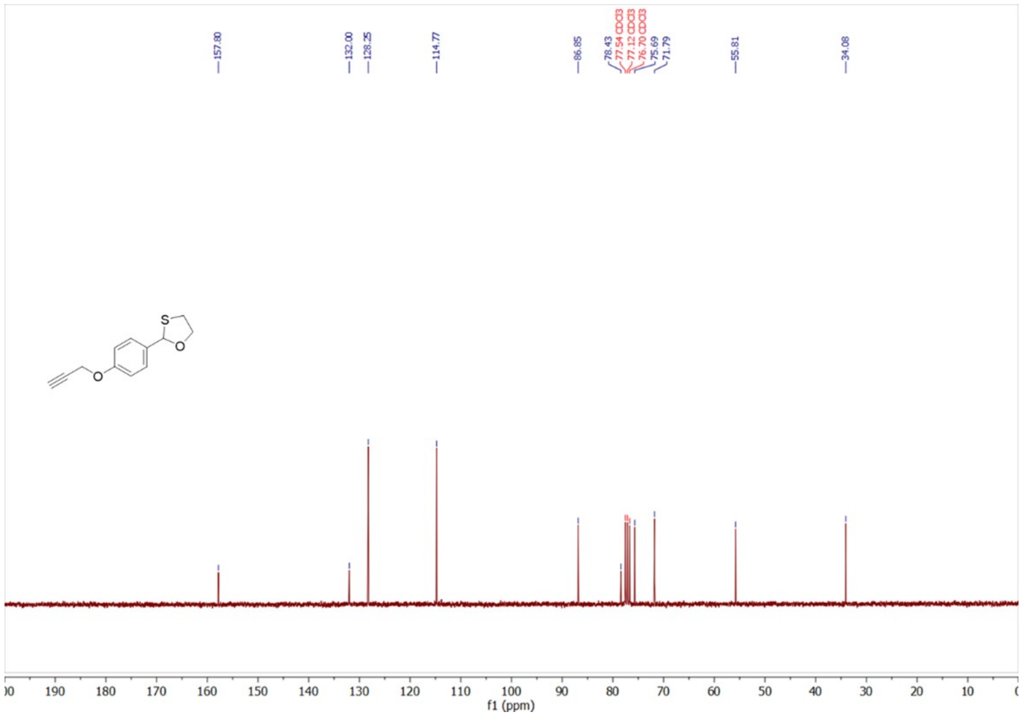

^{13}C NMR spectrum of **S4**

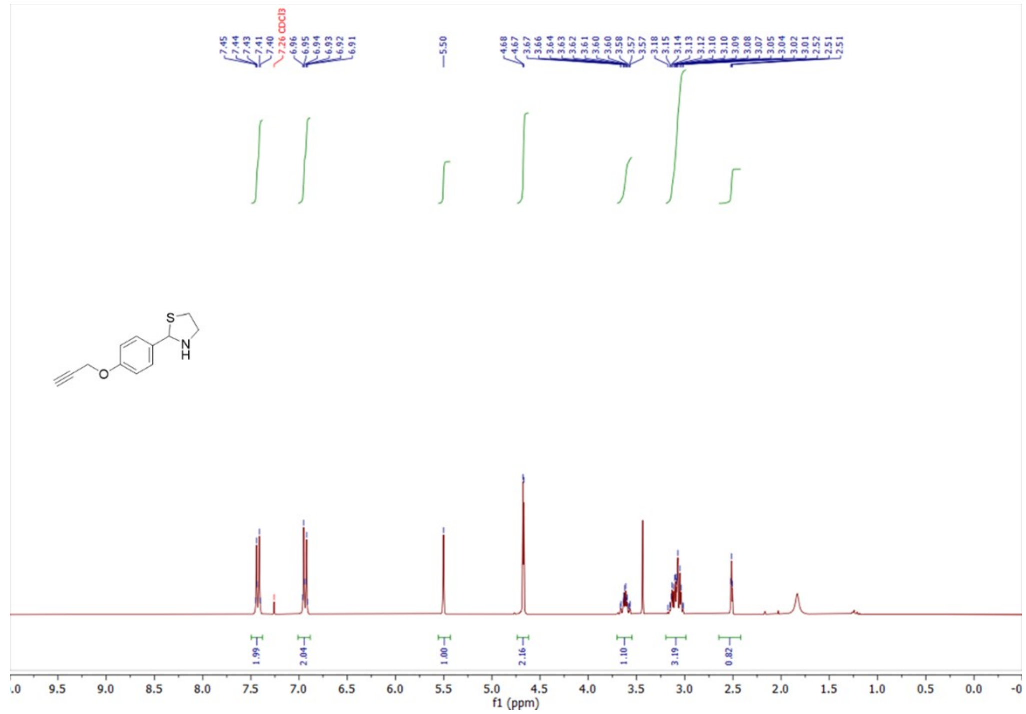

^{1}H NMR spectrum of **S5**

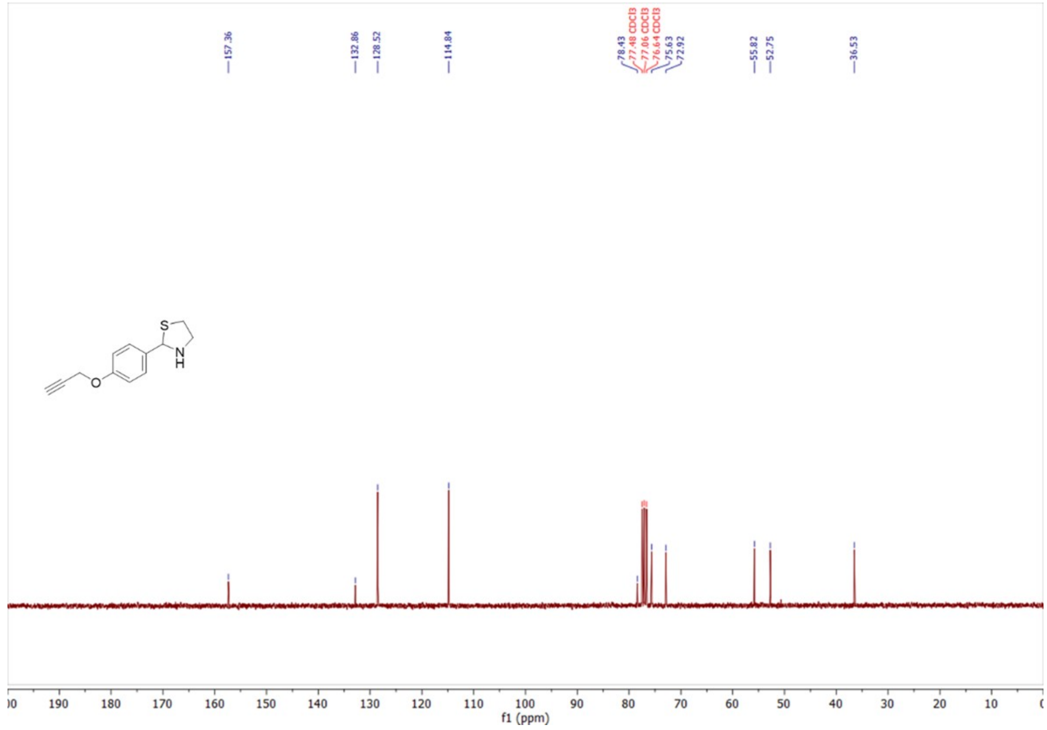

^{13}C NMR spectrum of **S5**

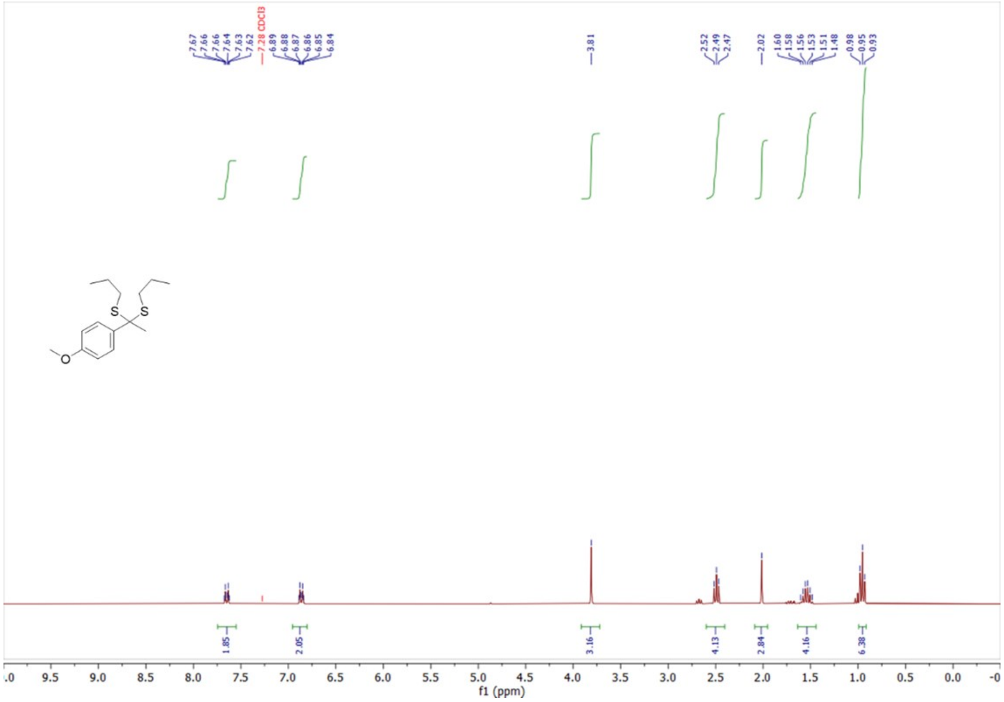

^{1}H NMR spectrum of **S6**

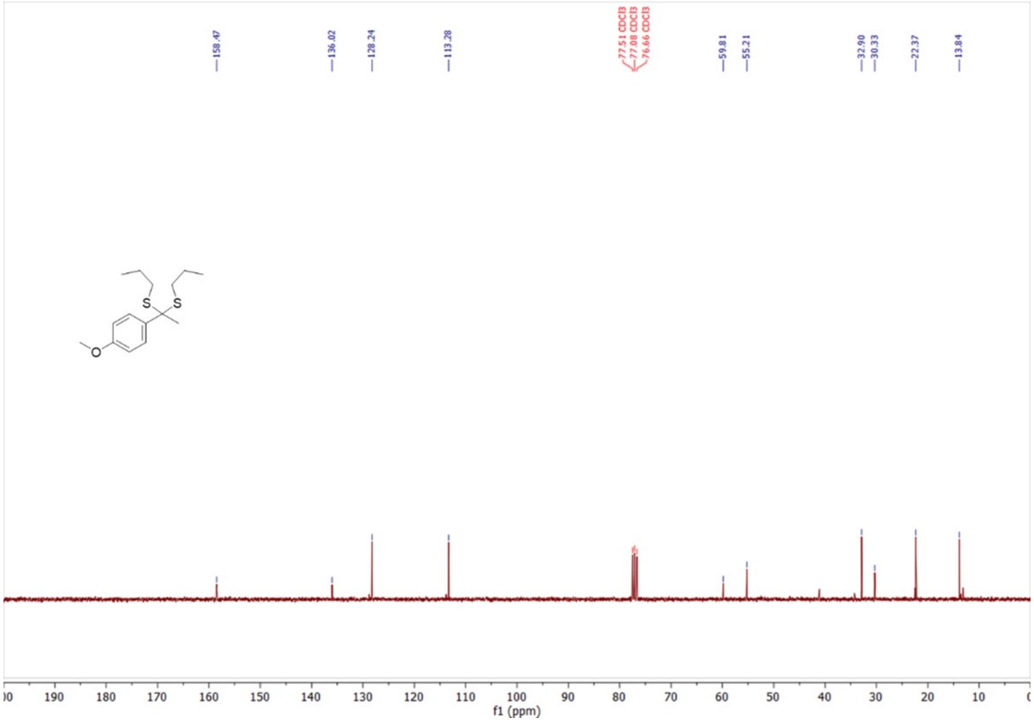

^{13}C NMR spectrum of **S6**

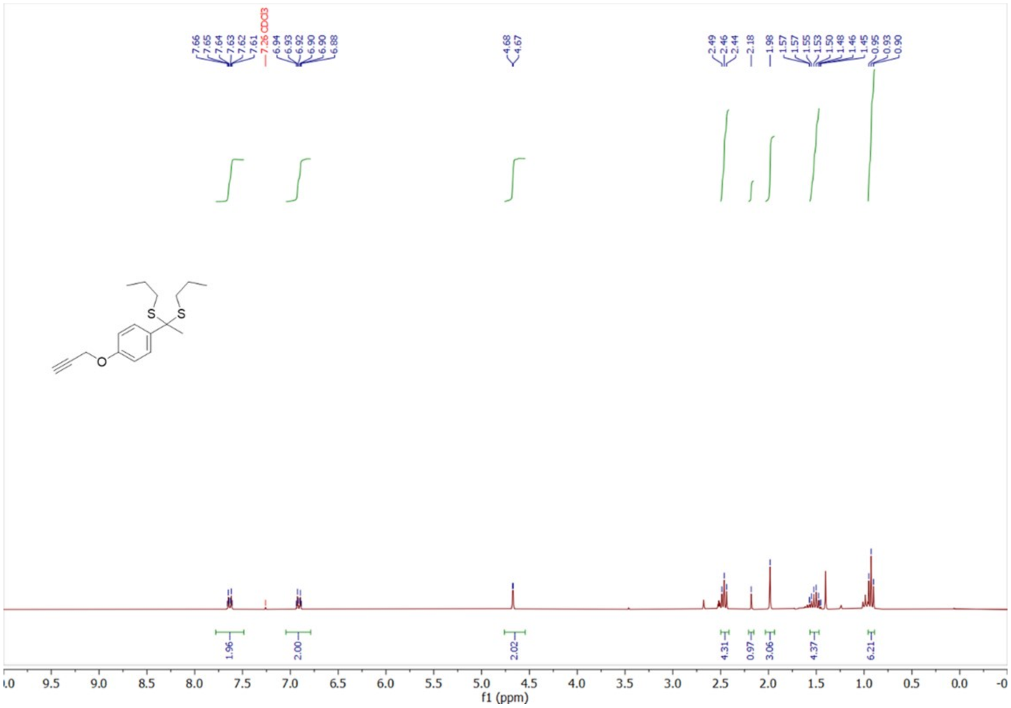

^{1}H NMR spectrum of **S7**

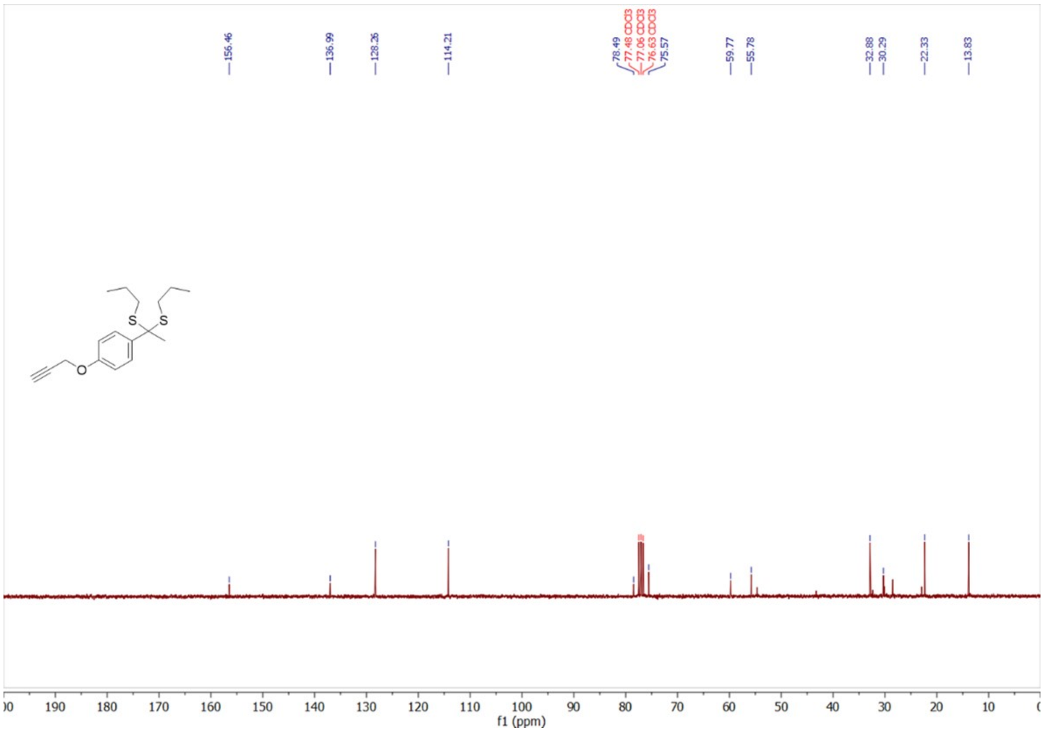

^{13}C NMR spectrum of **S7**

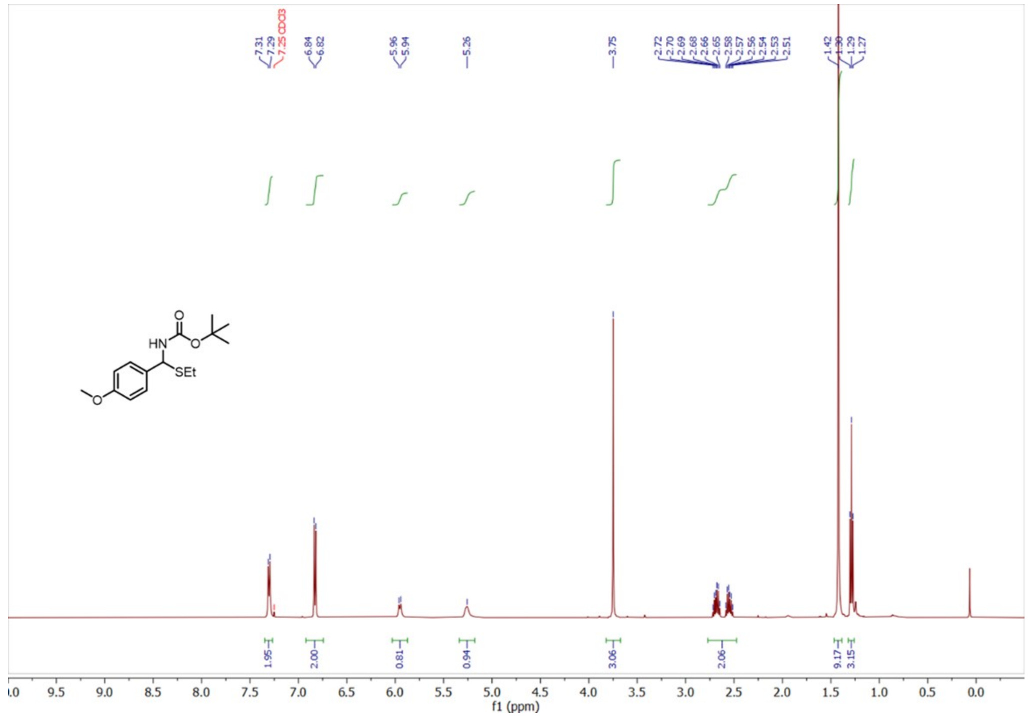

^{1}H NMR spectrum of **S8**

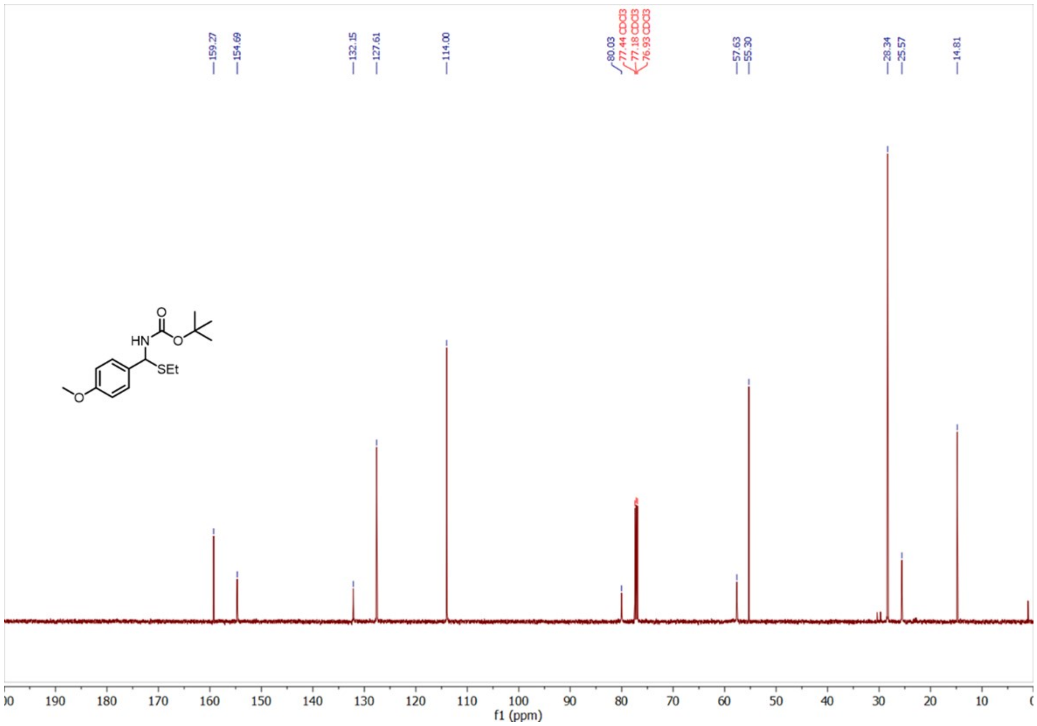

^{13}C NMR spectrum of **S8**

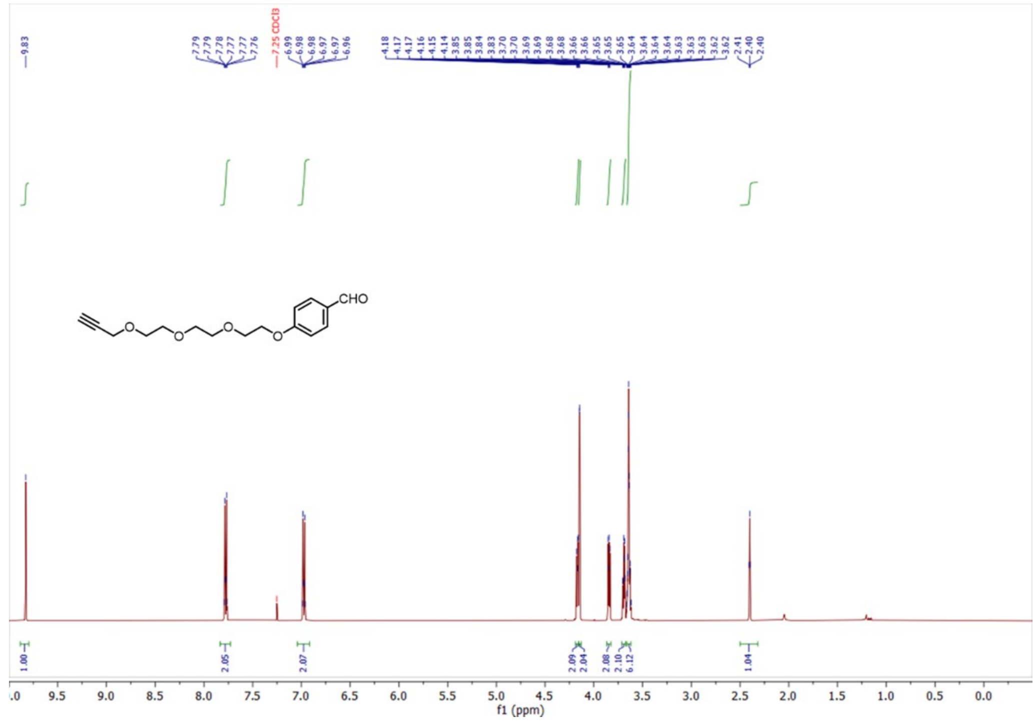

^{1}H NMR spectrum of **S9**

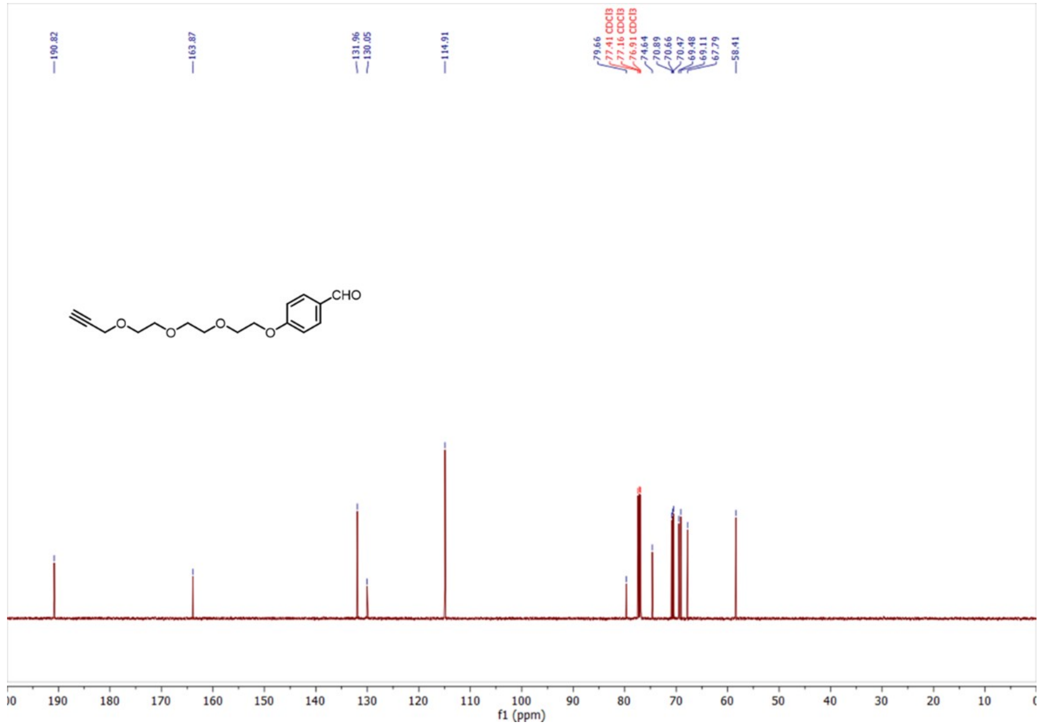

^{13}C NMR spectrum of **S9**

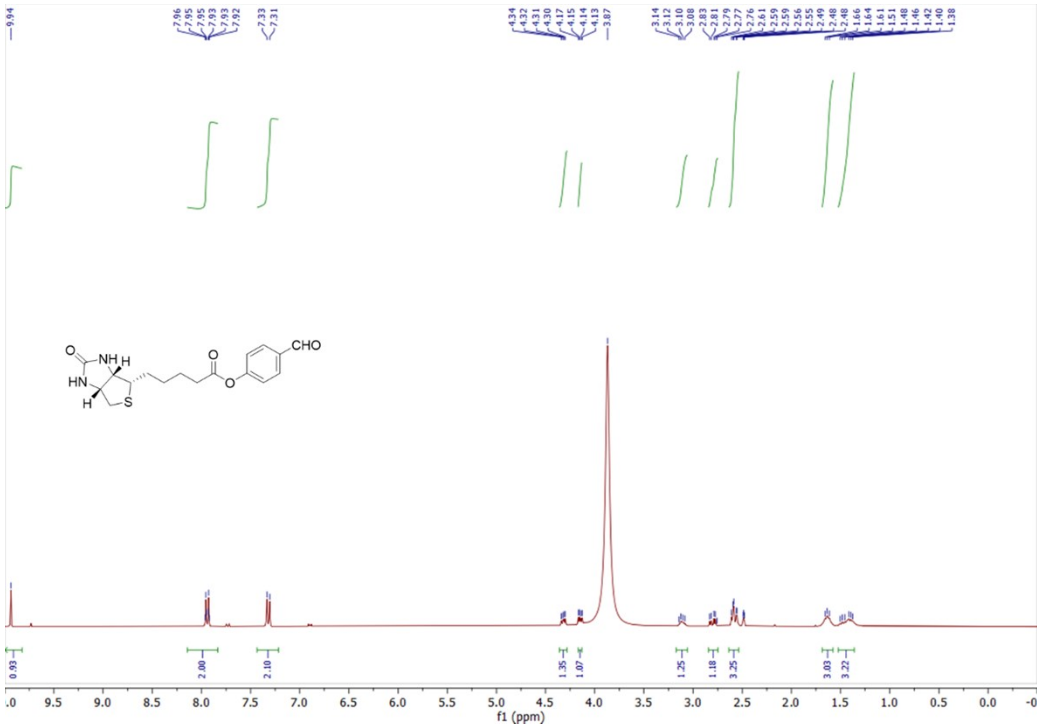

^{1}H NMR spectrum of **S10**

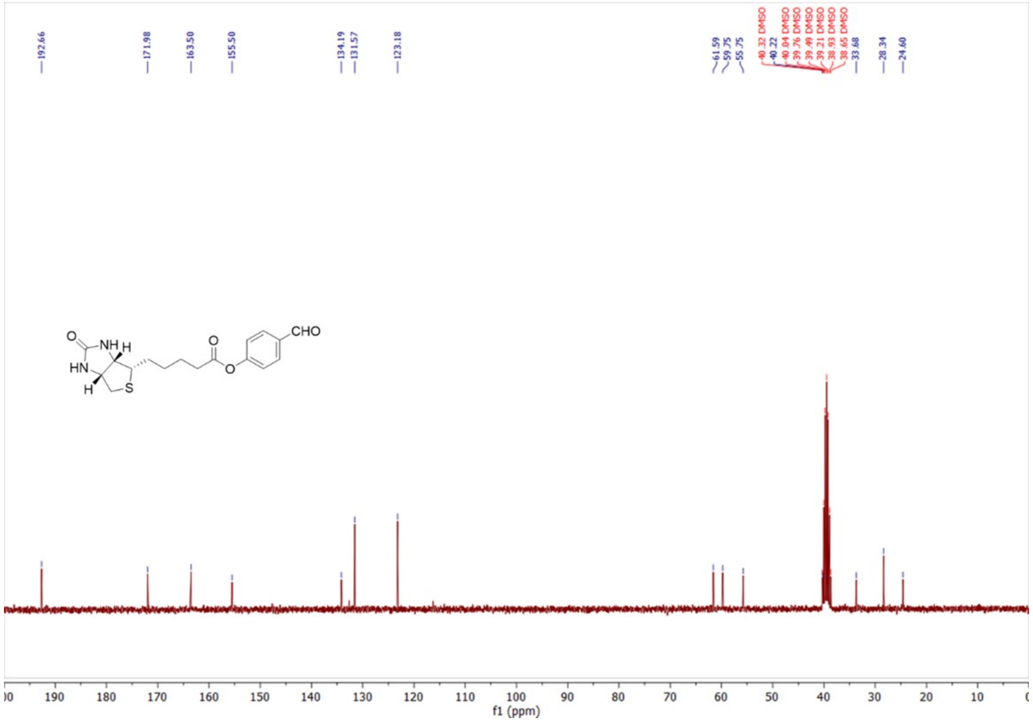

^{13}C NMR spectrum of **S10**

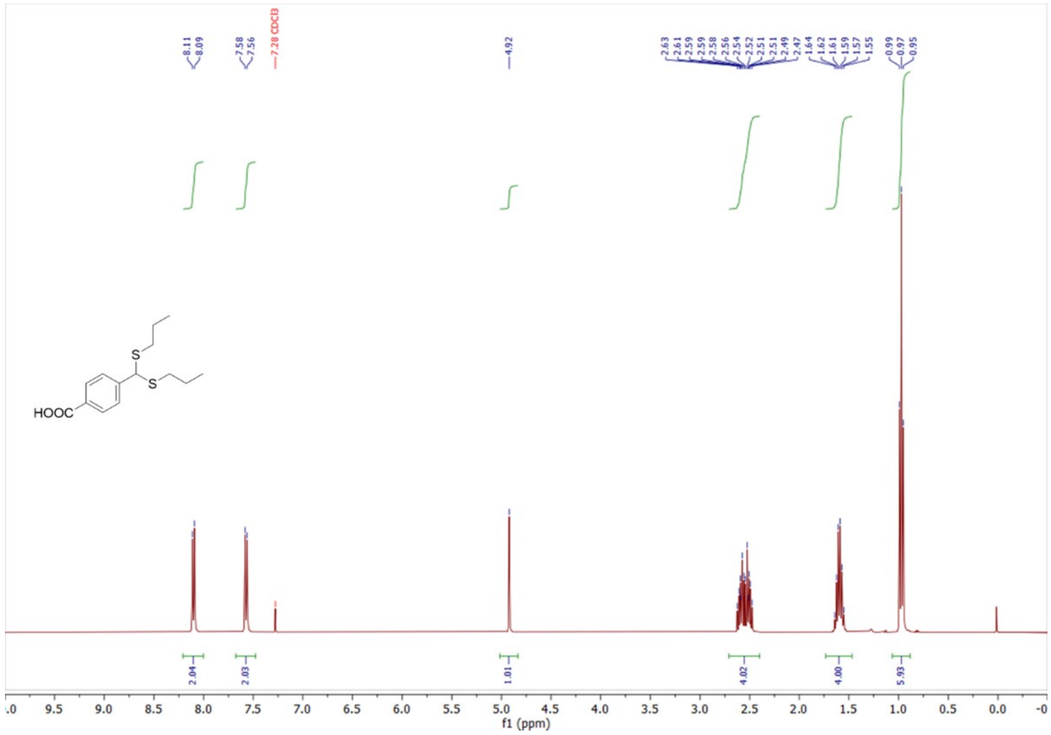

^{1}H NMR spectrum of **S11**

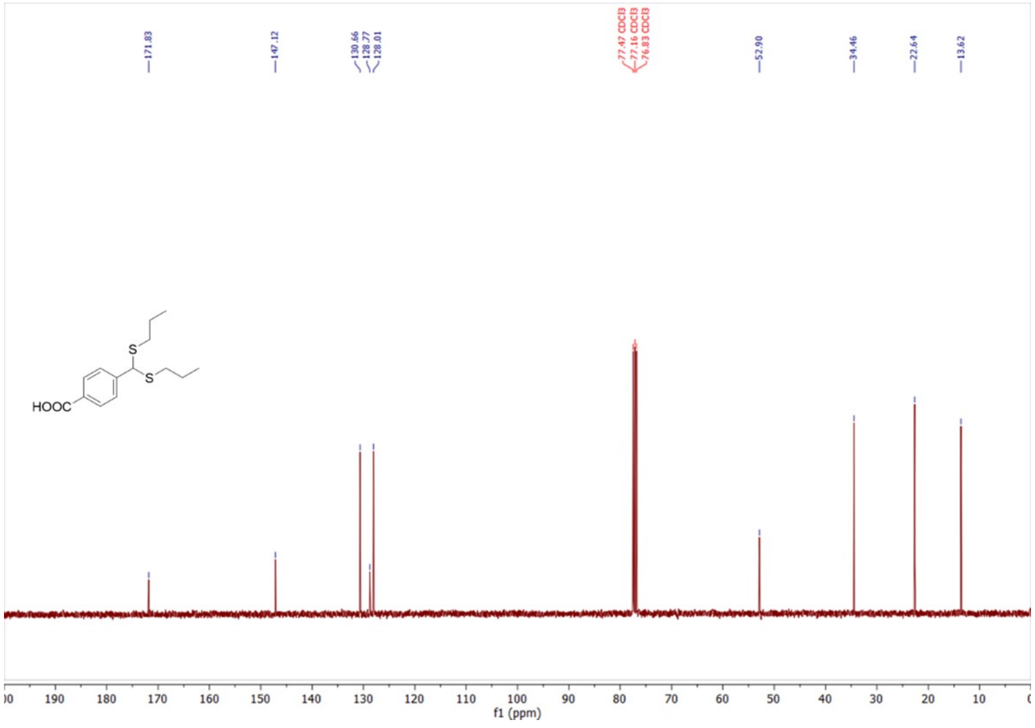

^{13}C NMR spectrum of **S11**

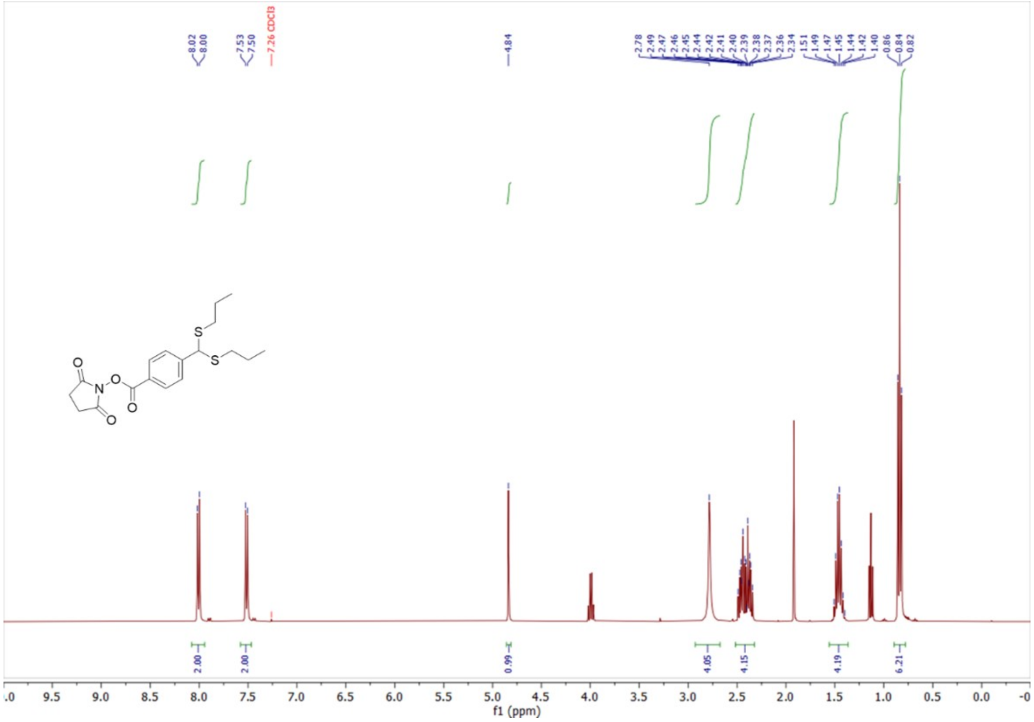

^{1}H NMR spectrum of **S12**

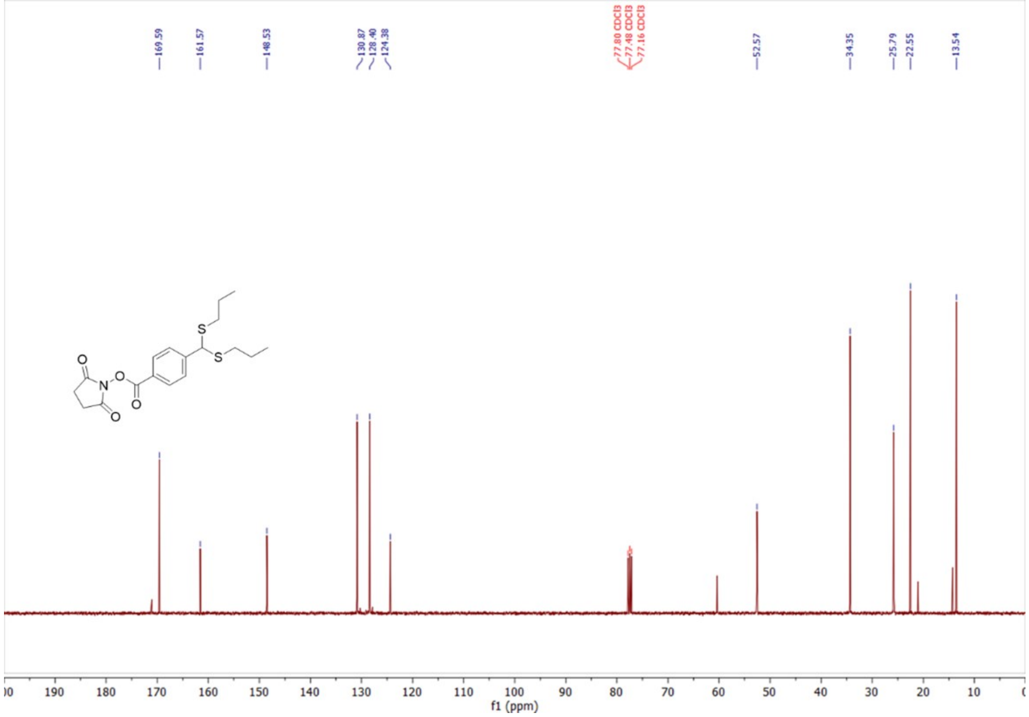

^{13}C NMR spectrum of **S12**

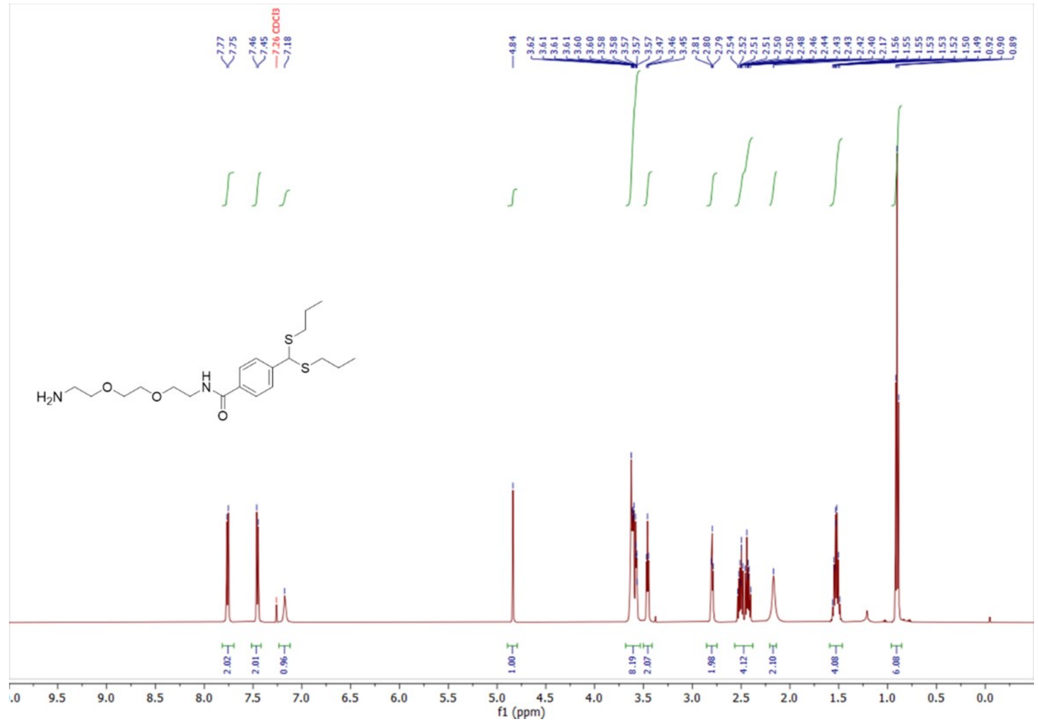

^{1}H NMR spectrum of **S13**

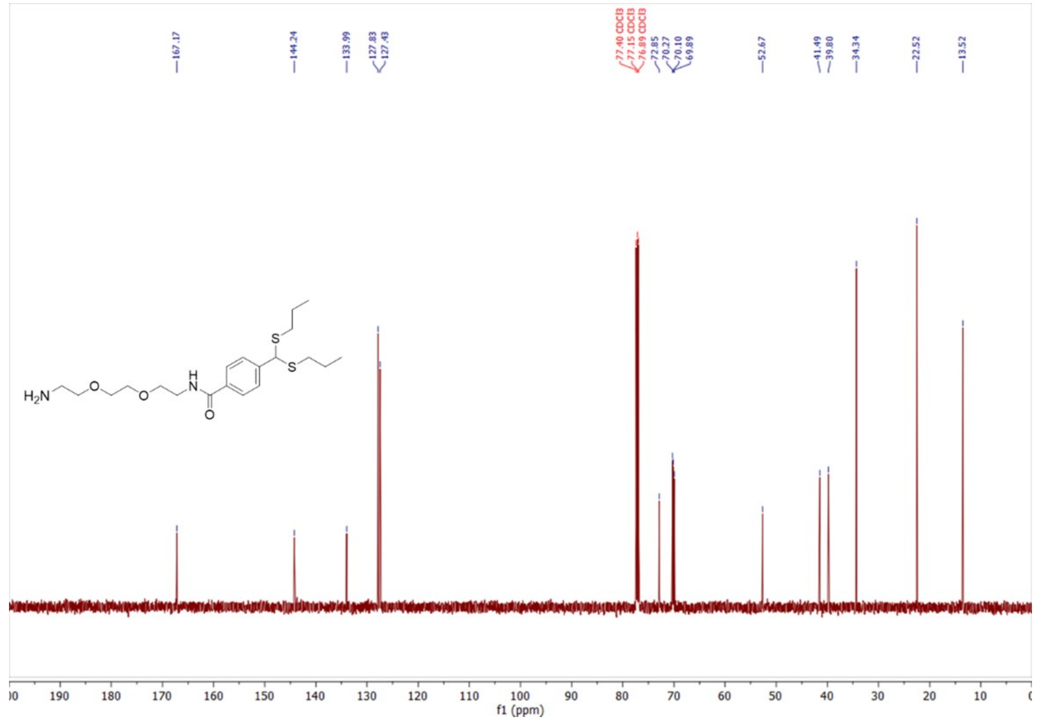

^{13}C NMR spectrum of **S13**

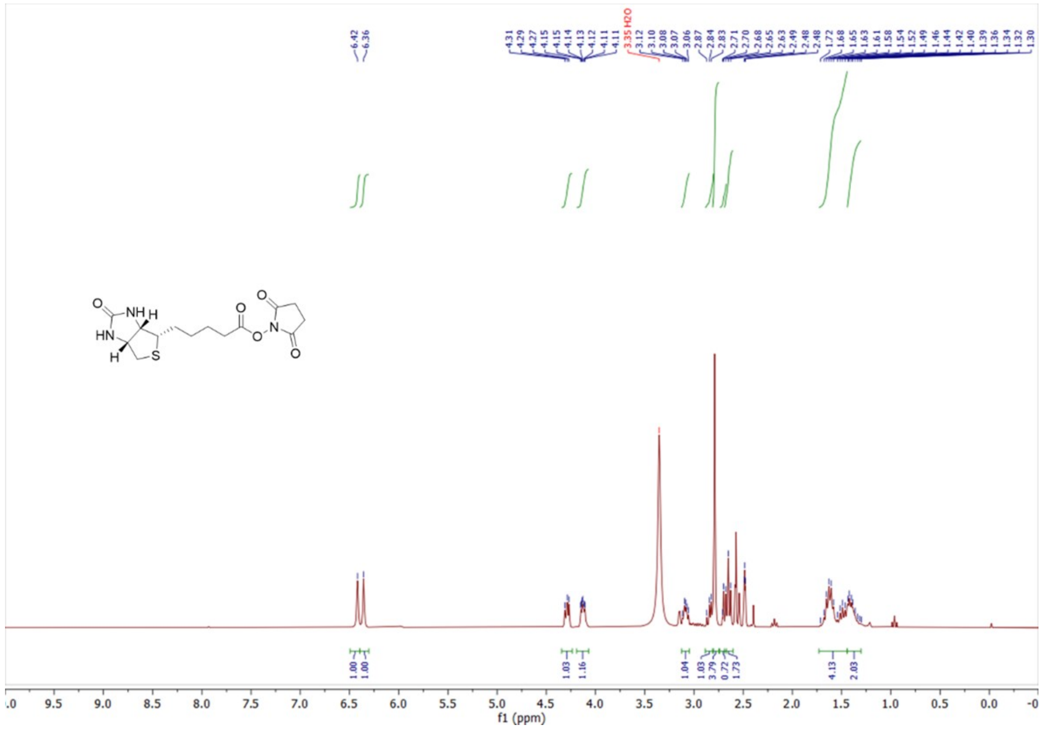

^{1}H NMR spectrum of **S14**

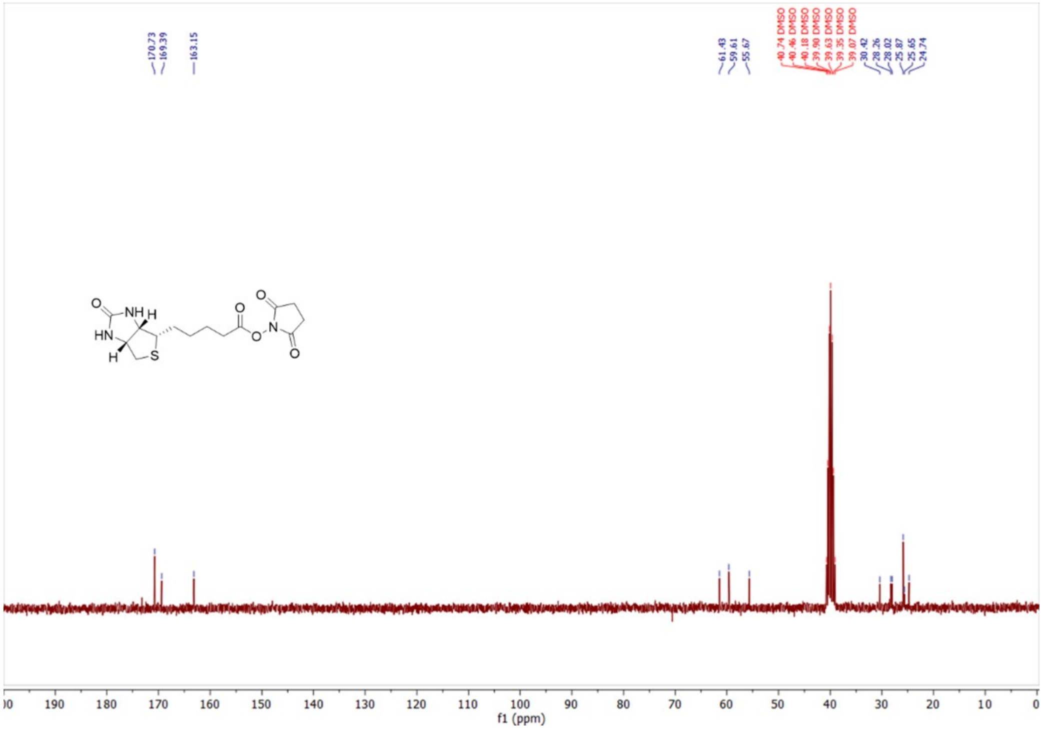

^{13}C NMR spectrum of **S14**

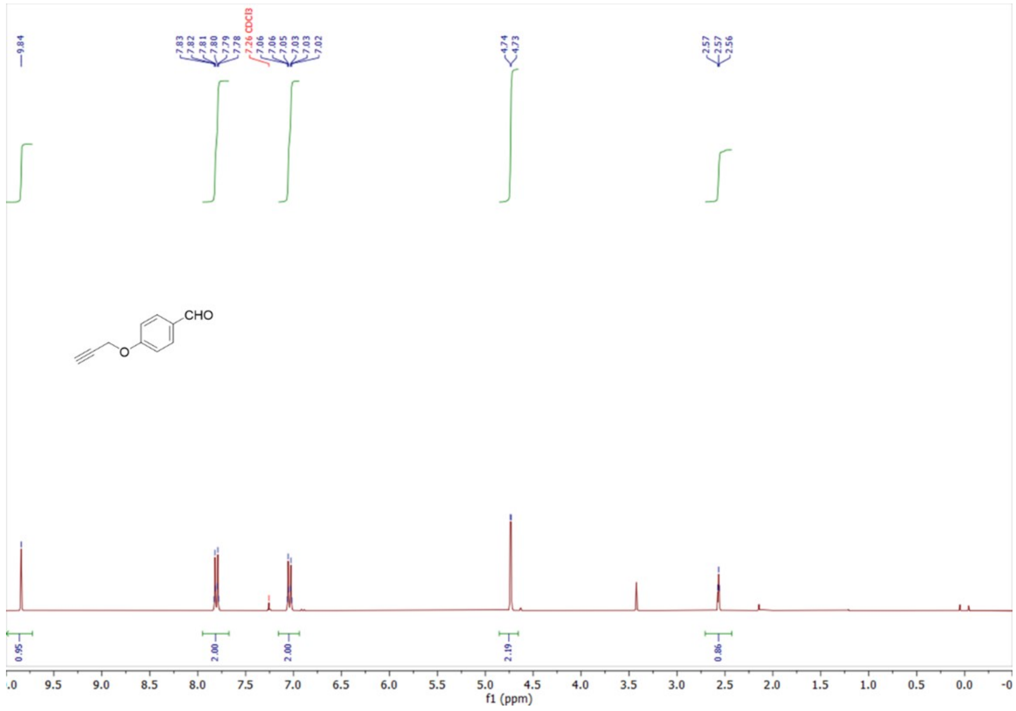

^{1}H NMR spectrum of **S15**

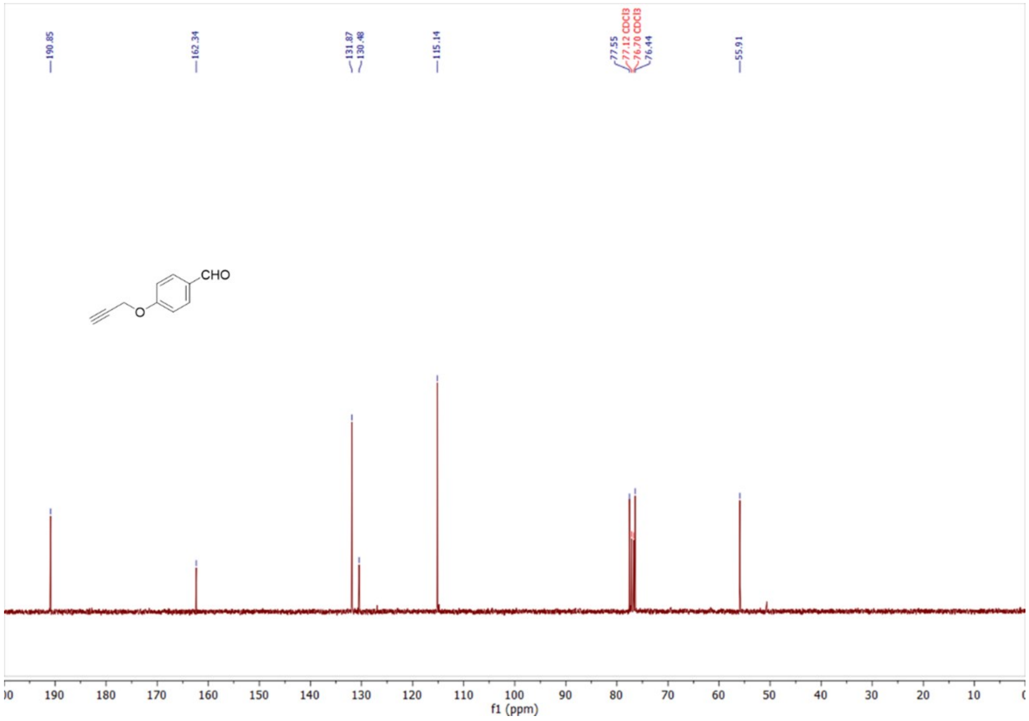

^{13}C NMR spectrum of **S15**

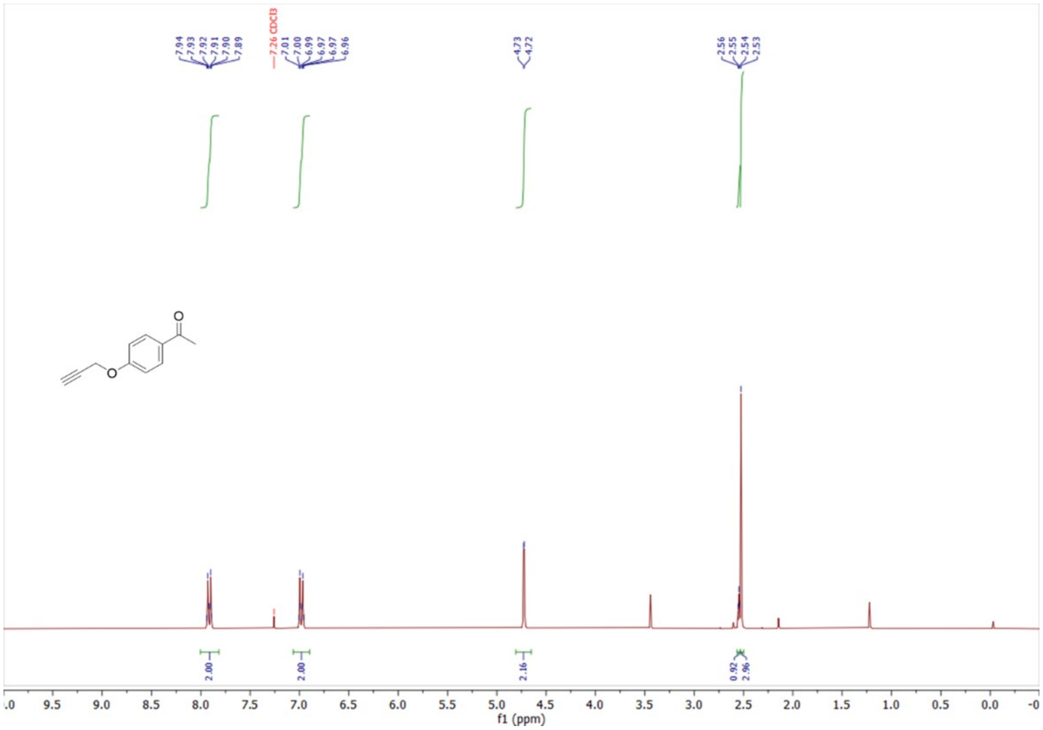

^{1}H NMR spectrum of **S16**

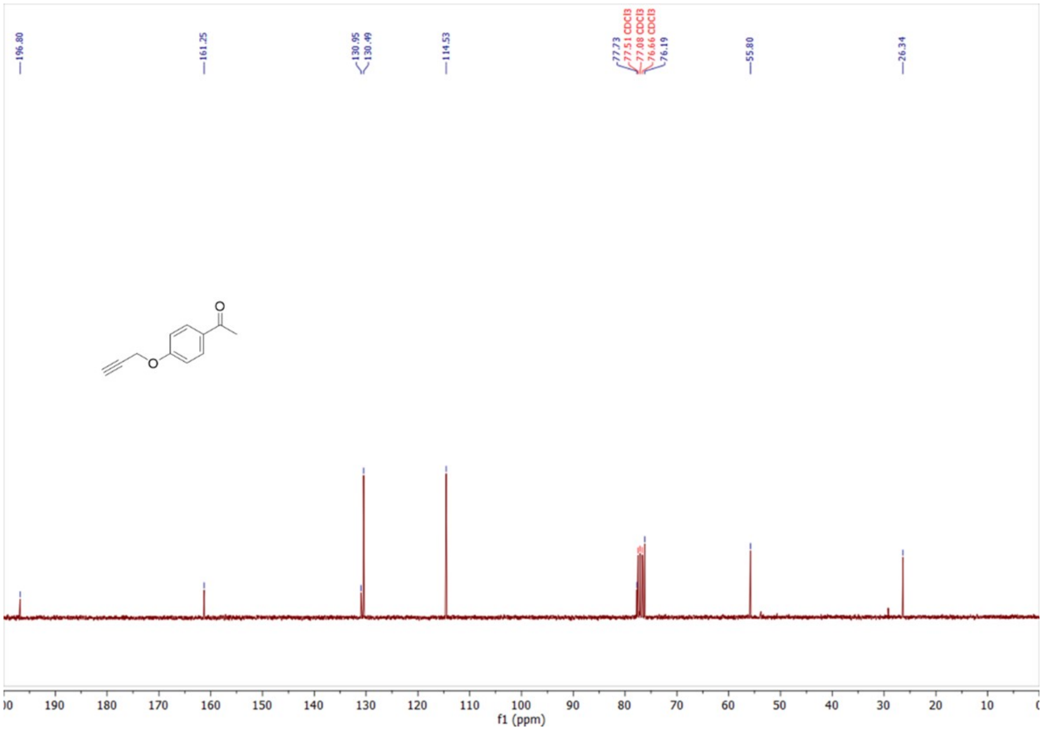

^{13}C NMR spectrum of **S16**

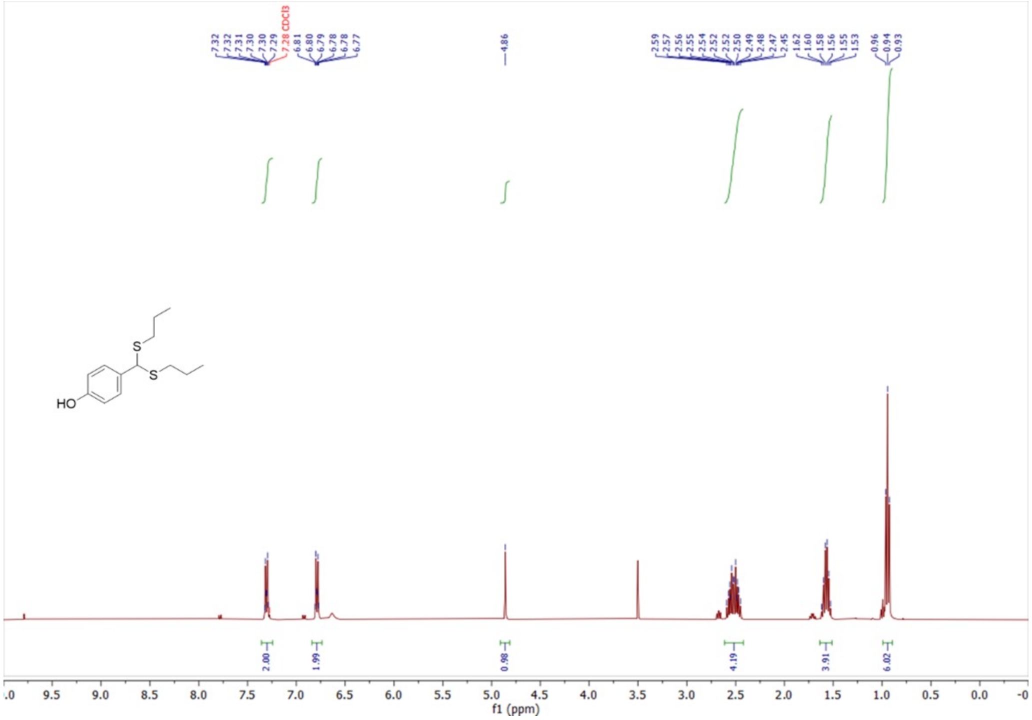

^{1}H NMR spectrum of **S17**

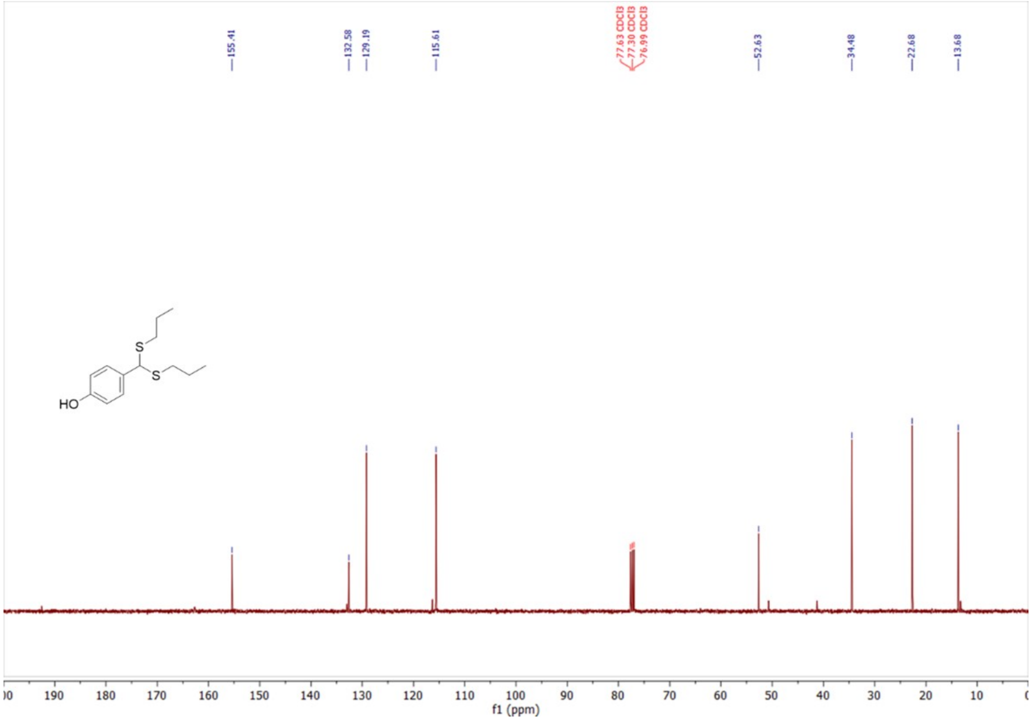

^{13}C NMR spectrum of **S17**

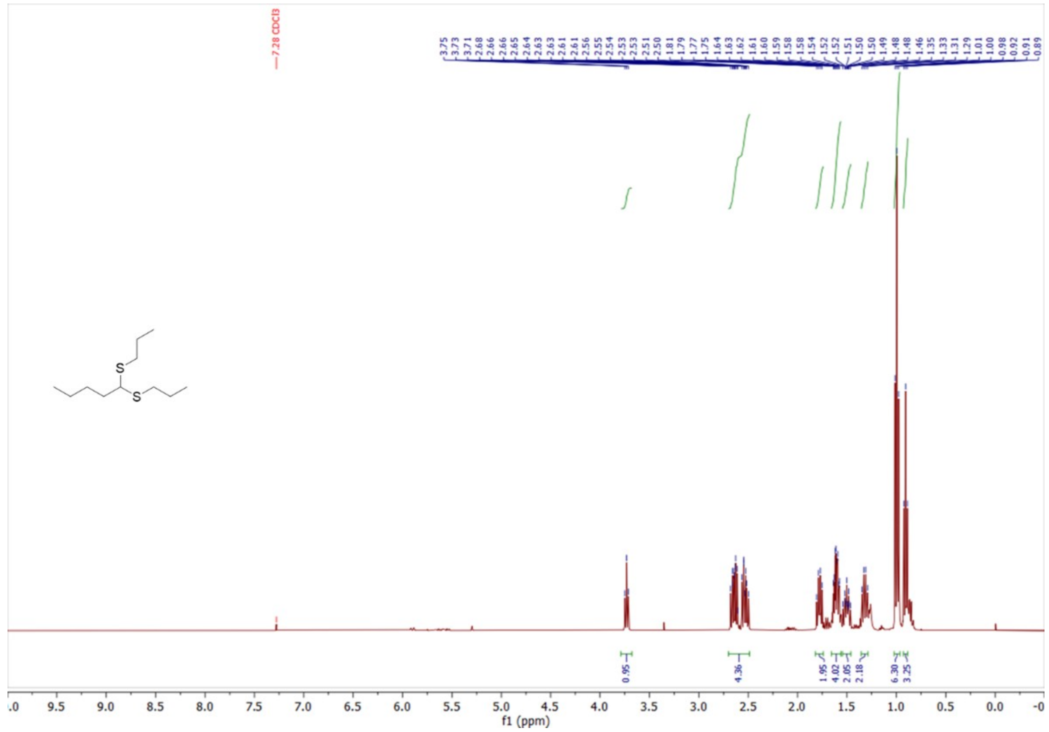

^{1}H NMR spectrum of **S18**

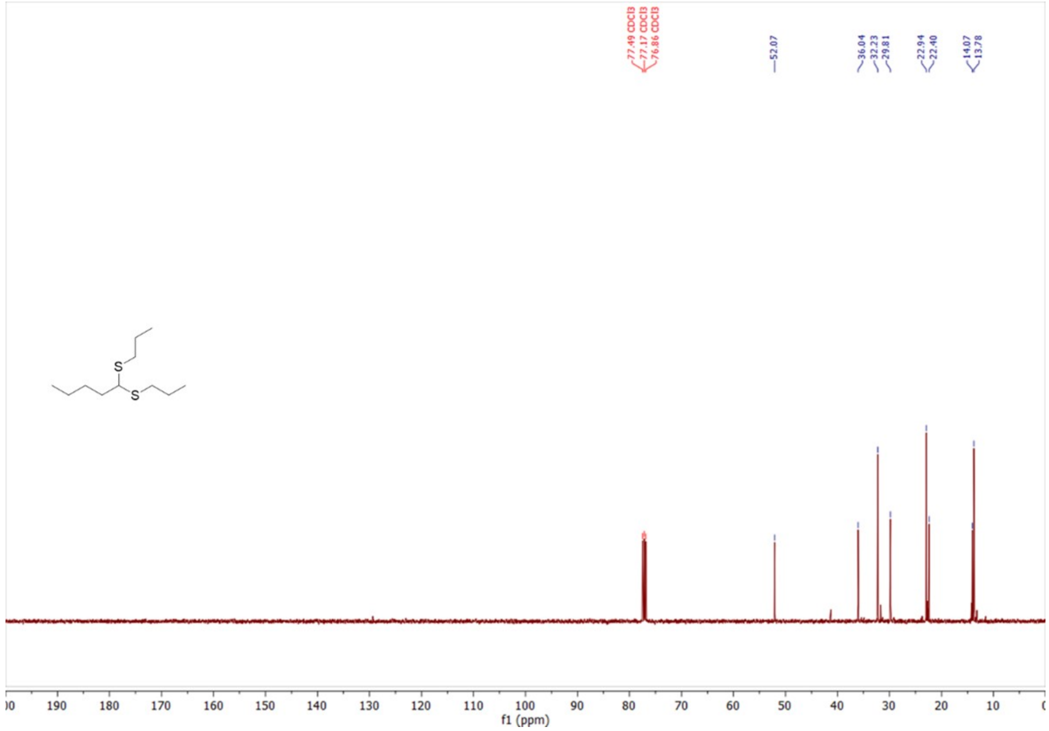

^{13}C NMR spectrum of **S18**
